# Miacalcic Enhances Rotator Cuff Injury Healing in Osteoporotic Mice by Stimulating Neovascularization via the JAK Pathway

**DOI:** 10.1155/mi/7332100

**Published:** 2026-06-03

**Authors:** Feng Mao, Jinguo Zhu, Xinting Feng, Chen Peng, Haoqiang Huang, Feng Xu, Minghao Tong, Qing Wang

**Affiliations:** ^1^ Department of Orthopedic Surgery, Kunshan Integrated TCM and Western Medicine Hospital, No. 388 Jinrong Road, Kunshan, 215332, Jiangsu, China; ^2^ Department of Orthopaedics, Nantong Tongzhou Hospital of Traditional Chinese Medicine, No. 8 Jianshe Road, Tongzhou, 226300, Jiangsu, China; ^3^ Department of Sports Medicine, Huashan Hospital, Fudan University, Shanghai, 200040, China, fudan.edu.cn; ^4^ Department of Sports Medicine, Peking University Shenzhen Hospital, Shenzhen, Guangdong, China, pkuszh.com; ^5^ Department of Orthopaedics, Kunshan Hospital of Chinese Medicine, No. 388 Zu Chong Zhi Road, Kunshan, 215300, Jiangsu, China

**Keywords:** angiogenesis, JAK signaling pathway, miacalcic, osteoporosis, rotator cuff tears, tendon-bone healing

## Abstract

**Objective:**

Rotator cuff tears (RCT) are prevalent among the elderly and often lead to significant shoulder pain. While both open and arthroscopic cuff repairs are effective, the recurrence of RCT post‐surgery remains high, with osteoporosis being a major contributing risk factor. Local angiogenesis and tendon‐bone healing are essential for optimal recovery after rotator cuff repair. This study investigates the effects of Miacalcic, an osteoporosis medication, on rotator cuff repair in osteoporotic conditions.

**Methods:**

To explore the mechanisms underlying Miacalcic’s action, we conducted RNA‐sequencing and cell‐based experiments on bone marrow‐derived mesenchymal stem cells (BMSC) and human umbilical vein endothelial cells (HUVECs). Additionally, an osteoporotic mouse model of RCT was utilized to assess in vivo effects.

**Results:**

Our findings revealed that Miacalcic had no significant effect on the proliferation of BMSCs, whereas it notably stimulated the proliferation of HUVECs. Miacalcic also significantly reduced apoptosis in HUVECs and enhanced their angiogenic potential. RNA‐Seq analysis indicated that Miacalcic primarily modulates the JAK signaling pathway, which plays a key role in angiogenesis. In vivo, Miacalcic treatment in an osteoporotic mouse model enhanced vascularization and facilitated tendon‐bone healing in the rotator cuff, leading to improved functional recovery following RCT.

**Conclusion:**

This study highlights the potential of Miacalcic as a therapeutic agent for promoting tendon‐bone healing in osteoporotic patients with rotator cuff injuries. By elucidating the mechanisms through which Miacalcic enhances angiogenesis and healing, our findings offer insights into potential strategies for improving post‐operative outcomes and reducing RCT recurrence. Further research is necessary to refine our understanding of the specific biological pathways involved and to explore the long‐term therapeutic benefits of Miacalcic.

## 1. Introduction

Rotator cuff tears (RCT) are a leading cause of shoulder pain, particularly in the elderly population [1, 2]. Open or arthroscopic cuff repair has proven to be an effective intervention, alleviating pain, and improving joint function [3, 4]. However, the high rate of re‐tearing post‐surgery remains a significant concern [5]. Several factors contribute to the risk of re‐tearing, including the size of the initial tear, tendon retraction, fatty infiltration, patient age, bone mineral density, and smoking [6, 7]. Research has identified osteoporosis as a risk factor for both RCT and postoperative re‐tearing [8, 9]. As such, managing osteoporosis in patients with RCT may be crucial for reducing the incidence of re‐tearing after surgery.

Osteoporosis has been established as an independent risk factor for RCT [10, 11]. A large retrospective study of 21,066 participants by Hong et al. [9] revealed that osteoporotic patients are 1.79 times more likely to develop RCT compared to non‐osteoporotic individuals, with women being at a higher risk than men. This association is not solely age‐related but is also likely linked to abnormal bone metabolism. Additionally, studies by Chuang and Hao highlighted the increased risk of re‐tearing after surgery in osteoporotic patients [12, 13]. The primary cause of re‐tearing is inadequate tendon‐bone integration, which is influenced by bone density, anchor pull‐out strength, osteoclast activity, and the interaction between decreasing estrogen levels and tendon‐bone healing [14–17].

The increased risk of re‐tearing in osteoporotic patients following rotator cuff repair surgery has led to several studies investigating the impact of osteoporosis medications on tendon‐bone healing. Drugs like Teriparatide and Abaloparatide have been shown to enhance bone density, improve tendon‐bone integration, and reduce the risk of re‐tearing [18, 19]. Other treatments, such as bone morphogenetic protein inhibitors and the combined use of Raloxifene and vitamin D, have also demonstrated efficacy in promoting tendon‐bone repair [20]. However, the role of bisphosphonates in rotator cuff repair remains controversial, with mixed results potentially arising from variations in study designs and populations.

Miacalcic, a nasal spray used for osteoporosis management, especially in postfracture women, is an inhibitor of bone resorption [21]. By reducing osteoclast activity, Miacalcic lowers the rate of bone resorption, which enhances bone density and decreases fracture risk [22, 23]. Long‐term administration can help preserve bone structural integrity, offering protection to patients [21, 24]. In addition, Miacalcic reduces perioperative bone loss and alleviates pain [25, 26]. Animal studies have shown that Miacalcic accelerates Achilles tendon‐to‐bone healing [27]. However, its effectiveness in the context of both osteoporosis and rotator cuff injury, as well as the underlying mechanisms, remains unclear.

Local angiogenesis and tendon‐bone healing are vital for postoperative recovery following rotator cuff surgery. Both human umbilical vein endothelial cells (HUVECs) and bone marrow‐derived mesenchymal stem cells (BMSCs) play critical roles in this process [28, 29]. HUVECs promote neovascularization, supplying essential nutrients and oxygen to damaged tissues [30, 31], while BMSCs enhance cellular proliferation, migration, and the secretion of therapeutic factors, which accelerate tendon‐bone repair and regeneration [32, 33]. Their combined action ensures successful tendon‐bone integration.

It remains unknown whether Miacalcic modulates these cellular activities to influence tendon‐bone healing. In this study, we sought to explore the mechanisms underlying Miacalcic’s effects on BMSCs and HUVECs. We first analyzed potential pathways and biological activities using RNA sequencing and bioinformatics, followed by cellular experiments on HUVECs to evaluate their proliferation, cell cycle progression, apoptosis, and tube‐forming ability. Finally, we assessed the in vivo effects of Miacalcic in a rotator cuff injury model in osteoporotic mice.

## 2. Methods

### 2.1. Animal Modeling and Ethics

This work involving animal subjects has been reviewed and approved by the Animal Ethics Committee (AEC) of Leadgene Biotechnology (Kunshan) Co., Ltd. All experimental procedures were conducted in strict compliance with the committee’s guidelines, which are based on internationally accepted principles for the ethical use and care of laboratory animals, including the “3Rs” (replacement, reduction, and refinement). The approved protocol number is 20241024‐0076‐01. At the experimental endpoint, mice were euthanized by intraperitoneal overdose of pentobarbital sodium (150 mg/kg), and death was confirmed before tissue collection.

Postmenopausal osteoporosis was induced in 12‐week‐old C57BL/6 females through bilateral ovariectomy, followed by 3‐month estrogen depletion. Bone microarchitecture parameters were quantified via high‐resolution μCT scanning (SkyScan 1276, Bruker) as per established protocols [34].

The surgical simulation of rotator cuff injury involved sequential procedures: (1) Intraperitoneal ketamine/xylazine anesthesia induction; (2) Dorsal positioning with forelimb abduction stabilized by 27G transcutaneous fixation; (3) 20 mm anterolateral approach exposing the glenohumeral joint; (4) Humeral head external rotation facilitating supraspinatus tendon exposure; (5) Complete tendon transection 3 mm proximal to the greater tuberosity insertion; and (6) Anatomic reattachment using modified Mason‐Allen sutures (Ethibond 5–0) through bicortical humeral tunnels.

Postoperative therapeutic intervention commenced immediately (day 0) with localized delivery of Miacalcic (40 mg·L^−1^) via ultrasound‐guided peri‐tendinous injections. Treatment regimens comprised 20 μL aliquots administered bis weekly for 8 consecutive weeks, following pharmacodynamic optimization from prior in vitro dose–response studies.

### 2.2. Cell Culture and Treatment

Primary HUVECs (Cat. #8000) were procured from ScienCell Research Laboratories (Carlsbad, CA, USA). As these are primary cells, an official cell line name and Research Resource Identifier (RRID) are not applicable. The cells were obtained directly from the supplier and were certified mycoplasma‐free. They were maintained under standardized endothelial culture conditions (ECM basal medium supplemented with 1% ECGS/5% FBS, ScienCell #1001) within a humidified 37°C/5% CO_2_ incubator. Cells underwent aseptic propagation through serial passaging (P4–P8) prior to experimental interventions. Pharmacological treatments included (1) gradient concentrations (0–80 μg·mL^−1^) of salmon calcitonin‐based Miacalcic (Livzon Pharmaceutical Group) and (2) co‐treatment with 1 μM baricitinib (JAK1/2 inhibitor; HY‐15315, MedChemExpress) dissolved in DMSO vehicle control.

### 2.3. Proliferation and Migration Experiments

HUVECs were seeded into 96‐well plates and treated with various concentrations of Miacalcic (0, 2.5, 5, 10, 20, 40, 80, 160, 320, 640, and 1280 µg/L) for 24 h when the cell confluence reached 50%–70%. Cell proliferation was assessed usingthea CCK8 assay to determine cell viability.

HUVECs were seeded into 6‐well plates and pre‐treated with or without Miacalcic for 24 h. BrdU solution was then added to the culture medium, and the cells were incubated in a CO_2_ incubator for 4 h. After incubation, cells were fixed with 4% paraformaldehyde (PFA). Subsequent experiments were performed according to the manufacturer’s instructions provided with the BrdU assay kit (Servicebio, Wuhan, China).

HUVECs were seeded into six‐well plates, and when the cells reached 100% confluence, a straight line was scratched at the bottom of the well using a sterile pipette tip. Detached cells were washed away, and the cells were cultured in a base medium containing 1% fetal bovine serum (FBS). The control group received no Miacalcic treatment, while the experimental group was treated with Miacalcic at a concentration of 40 µg/L.

Transwell assays were conducted using Transwell cell culture inserts (Corning, NJ). HUVECs were seeded in the upper chamber, and endothelial cell medium with or without Miacalcic was added to the lower chamber. The cells were incubated in a CO_2_ incubator for 6 h. After incubation, the cells in the upper chamber were gently scraped with cotton swabs. Cells that had migrated through the membrane were stained with 1% crystal violet (Solarbio, Beijing, China) and visualized under a microscope.

### 2.4. Apoptosis‐Related Experiment

Apoptotic cell rates were assessed using the Annexin V‐FITC apoptosis assay kit (Beyotime Biotechnology, Shanghai, China), following the manufacturer’s instructions.

### 2.5. Angiogenesis Related Experiment

Prior to cell inoculation, 50 µL of Matrigel (BD Biosciences) was spread evenly on the bottom of a 96‐well plate and incubated for 30–60 min to allow gelation. HUVECs were then seeded onto the Matrigel‐coated wells and incubated in a CO_2_ incubator for 4 h. Blood vessel formation was observed and documented under a microscope.

### 2.6. RNA Sequencing

Following 24 h Miacalcic exposure, transcriptomic profiling was conducted through RNA isolation with the RNeasy Mini Kit (Qiagen, Hilden, Germany) [35], with subsequent library construction using the TruSeq RNA Sample Preparation Kit (Illumina, USA). Sequencing on Illumina platforms by Shanghai Biotechnology Corporation generated paired‐end reads that were mapped against the Rnor 6.0 reference genome (Hisat2 v2.0; ≤2 mismatches) and subsequently processed through Stringtie (v1.3.0) for FPKM‐based transcript quantification. Bioinformatics interrogation identified differentially expressed genes (DEGs) meeting |log_2_FC| ≥1 with Benjamini–Hochberg adjusted *p* ≤ 0.05 thresholds, which were functionally annotated through KEGG pathway enrichment analysis (http://www.genome.ad.jp/kegg) implemented in R computational workflows.

### 2.7. Bioinformatics Analysis

To delineate mechanistic networks, KEGG pathway enrichment was executed through the KEGG API (http://www.genome.ad.jp/kegg) within R computational frameworks [36], while functional annotation of differentially expressed transcripts (|log_2_FC|≥1, FDR <0.05) was implemented via the DAVID knowledgebase for Gene Ontology (GO) categorization spanning biological processes, molecular functions, and cellular compartments.

### 2.8. Western Blot (WB)

Protein immunoblotting analyses were conducted per established protocols [37]. Cellular lysates were prepared using WB/IP Lysis Buffer (YEASEN, Shanghai, China) containing a protease inhibitor cocktail, followed by 15 min ice incubation and centrifugation (10,000 rpm, 4°C, 15 min) to obtain clarified supernatants. Protein concentrations were determined via the BCA assay (YEASEN), with 30 μg aliquots resolved on 10% SDS‐PAGE gels prior to electrophoretic transfer onto PVDF membranes. Membranes underwent sequential blocking (5% skim milk, RT/1h) and antibody incubations: primary antibodies (Cyclin D1/D3, Bcl‐2, Bax, Caspase‐3, CD31, VEGFa, JAK1/p‐JAK1, STAT3/p‐STAT3, and Tubulin; Abcam/Affinity, 1:1000) were subjected to overnight incubation at 4°C, followed by 1 h RT exposure to HRP‐conjugated secondary antibodies. Chemiluminescent signals were captured using Image Lab software, with densitometric quantification performed in ImageJ (NIH).

### 2.9. Near‐Infrared (NIR) Probe: Vascular Tracing

For real‐time visualization of vascular structures, we used a B‐NaYF_4_:Yb/Er@NaYF_4_ NIR probe, which allows the dynamic assessment of vessels, including those in the localized rotator cuff injury site [38, 39]. 2 weeks post‐RCT injury in mice, the animals were anesthetized with carbon dioxide and injected with the NIR probe via the tail vein. Vascular recovery in the rotator cuff area was monitored and visualized within 5 min postinjection using dynamic imaging.

### 2.10. Gait Analysis

Locomotor performance in mice was evaluated using the CatWalk XT gait assessment system (CatWalk XT; Noldus, The Netherlands). In the RCT model and calcitonin topical treatment groups, surgery was limited to the right forelimb, leaving the contralateral limb intact to avoid postural compensation that could influence gait analysis. Control mice underwent no surgical intervention. To acclimate the mice to the experimental conditions, an intensive training schedule was implemented. Mice were required to traverse a confined passage over a light‐emitting glass runway, ensuring continuous movement with minimal interruptions for no less than 7 days. The mice’s footprints, captured by a built‐in illumination diffraction method, provided detailed insights into the size and pressure distribution of each footprint. The emitted light’s brightness correlated with the pressure intensity, and high‐resolution cameras recorded the footprints. The footprints were automatically analyzed using CatWalk XT 10.0 software to evaluate gait parameters.

### 2.11. Histological Examination

Histological analysis was performed to evaluate tendon‐bone healing and regeneration. Tissue specimens were fixed in PFA, decalcified in an EDTA decalcifying solution, and embedded in paraffin. Sections were cut into 5 µm slices using a microtome for subsequent H&E staining and CD31 immunofluorescence staining to assess tissue morphology and vascularization.

### 2.12. Biomechanical Testing

The biomechanical properties, including the failure load and ultimate strength, were measured using a materials testing system. Specimens were secured in fixtures and subjected to uniaxial tensile testing at a 60° abduction angle, approximating the anatomical position of the specimens. Tension was applied at a rate of 1 mm per minute until the failure occurred.

### 2.13. Micro‐CT

Micro‐CT analysis was performed on anesthetized mice placed on the sample stage of a MicroCT scanner (Skyscan 1176). The mice were securely fixed to ensure that the femur was in the optimal scanning position. Tomographic scans were performed, and the reconstructed 3D images were analyzed for bone density using professional image analysis software (CT‐Analyzer, CTVox).

### 2.14. Statistical Analysis

All experiments were performed in triplicate. Statistical analysis was conducted using GraphPad Prism 9.5.1 and ImageJ software. Data were analyzed using ordinary one‐way ANOVA (followed by appropriate post hoc tests, such as Bonferroni or Tukey’s, for multiple comparisons) and the *t*‐test, and the results were expressed as the mean ± SD. A *p*‐value of <0.05 was considered statistically significant.

## 3. Results

### 3.1. Effect of Miacalcic on Proliferation/Apoptosis of HUVEC and BMSC

To evaluate Miacalcic’s impact on HUVEC and BMSC viability, cells were treated with Miacalcic at concentrations ranging from 0 to 1280 µg/mL, following established protocols. After 24 h of exposure, CCK‐8 assays demonstrated concentration‐dependent effects on HUVECs: proliferation was enhanced at lower concentrations (≤40 µg/mL) but suppressed at higher doses (Figure 1A). BMSCs showed no proliferative response at any concentration (Supporting Information 1: Figure S1), leading to their exclusion from subsequent experiments. The optimal concentration of 40 µg/mL was chosen for further HUVEC studies. BrdU incorporation assays validated the proliferative effect, showing significantly increased DNA synthesis in Miacalcic‐treated HUVECs versus controls (Figure 1B,D). Flow cytometric apoptosis analysis revealed substantially reduced apoptotic rates in treated cells (Figure 1C,E). WB analysis confirmed that 40 µg/mL Miacalcic concurrently activated proliferative pathways (upregulated protein expression) and inhibited apoptotic signaling (downregulated markers) in HUVECs (Figure 1F–K; Supporting Information 2: File S1). These data collectively indicate that Miacalcic selectively enhances proliferation, reduces apoptosis, and improves migration capacity in HUVECs, while exhibiting no detectable biological effects on BMSCs.

Figure 1Miacalcic enhances cell proliferation and suppresses apoptotic activity in HUVEC cultures. (A) Dose‐dependent effects of Miacalcic on HUVEC viability were quantified through 24 h exposure experiments. Cellular metabolic activity showed significant variations across concentration gradients, as determined by one‐way ANOVA with Bonferroni correction. Normalized data are expressed as relative values, with statistical significance denoted by  ^∗^ *p*  < 0.05,  ^∗∗∗^ *p*  < 0.001,  ^∗∗∗∗^ *p*  < 0.0001.(B) BrdU incorporation assays revealed the proliferative impact of 40 μg/L Miacalcic following 24 h incubation.(C) Flow cytometric analysis demonstrated altered apoptotic rates in HUVEC populations pre‐ and post‐treatment with 40 μg/L Miacalcic. (D, E) Quantitative evaluation of BrdU incorporation and apoptosis data using Student’s t‐test (*n* = 3 biological replicates) confirmed significant differences ( ^∗∗∗^ *p* < 0.001) between experimental groups, with values expressed as mean ± SD. (F–K) Western blotting analysis quantified expression profiles of proliferation/apoptosis‐associated proteins in treated HUVECs. Tubulin served as the loading control for protein normalization. Statistical comparisons between groups were performed using Student’s *t*‐test (*n* = 3), with data presented as mean ± SD ( ^∗^ *p*  < 0.05,  ^∗∗^ *p*  < 0.01,  ^∗∗∗^ *p*  < 0.001).

**Figure:**
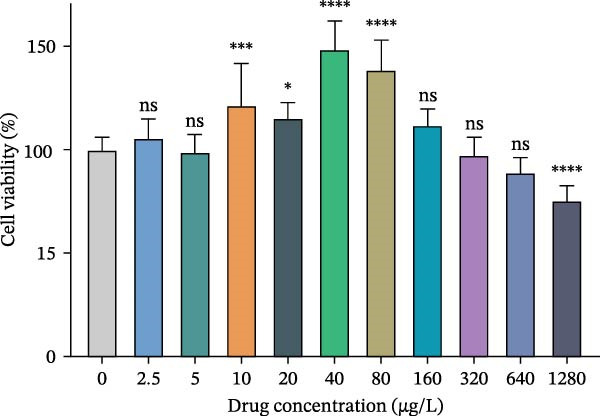
(A)

**Figure:**
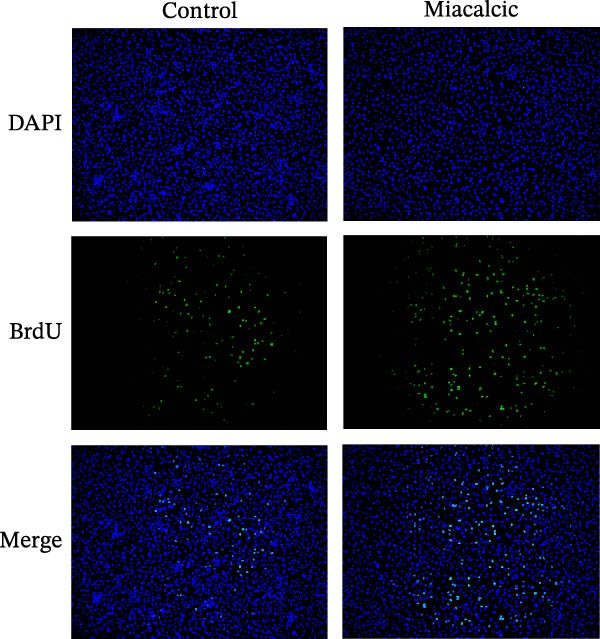
(B)

**Figure:**
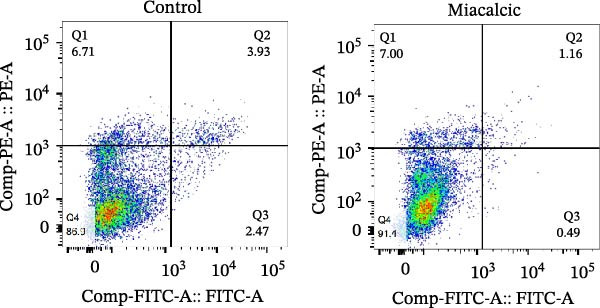
(C)

**Figure:**
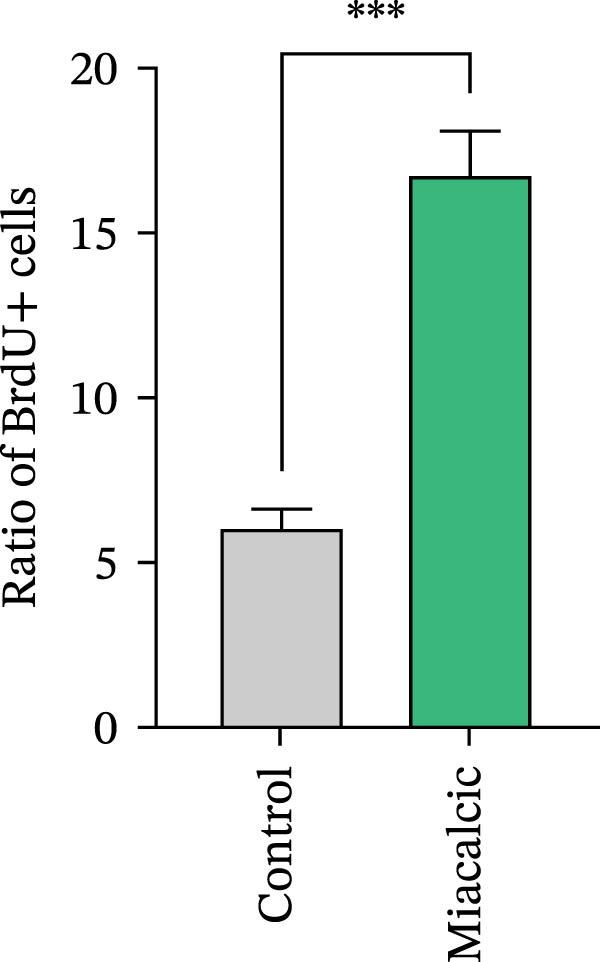
(D)

**Figure:**
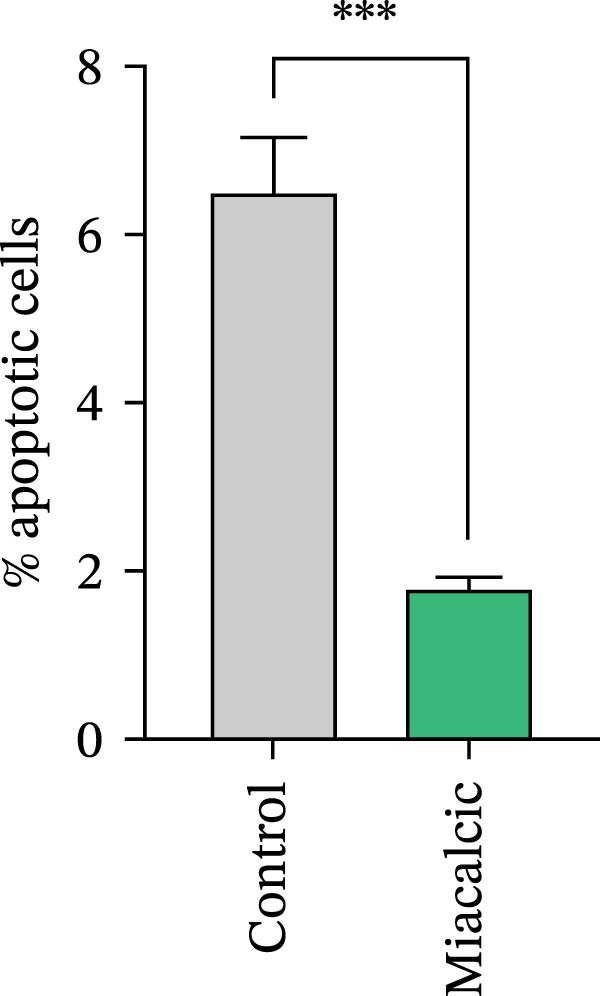
(E)

**Figure:**
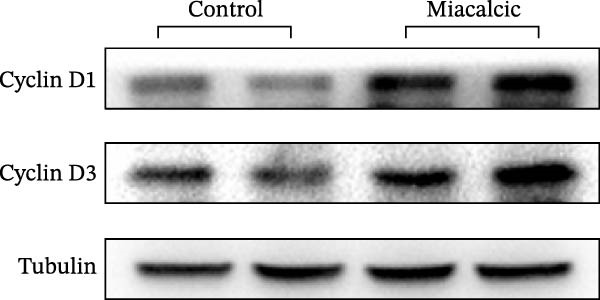
(F)

**Figure:**
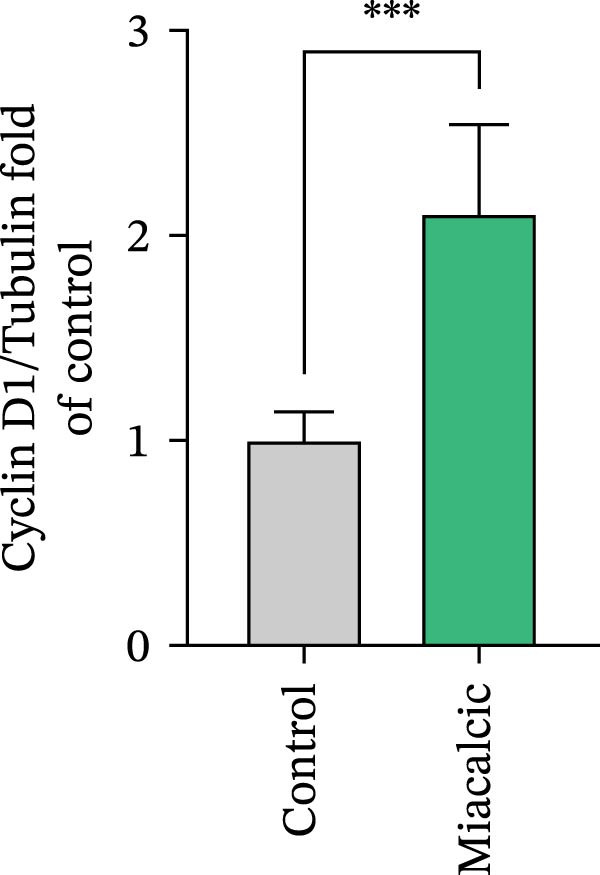
(G)

**Figure:**
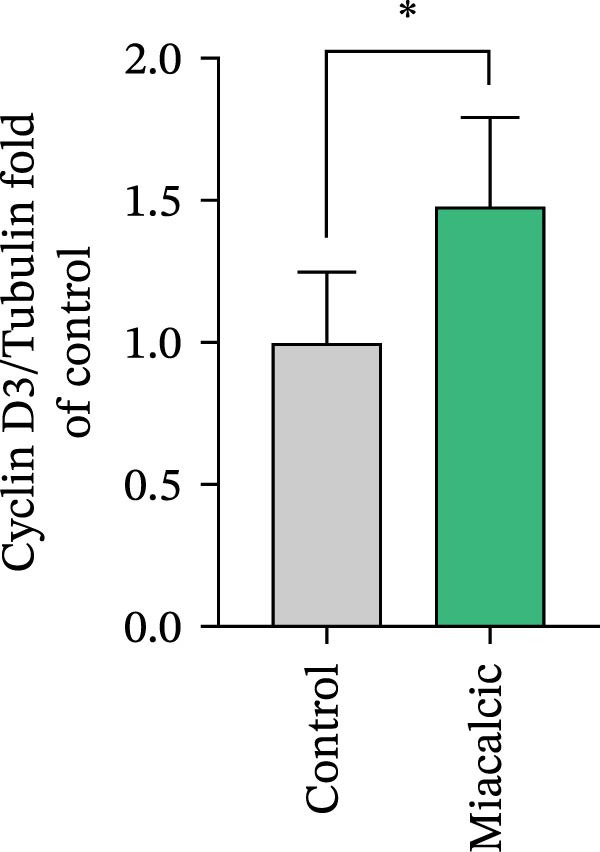
(H)

**Figure:**
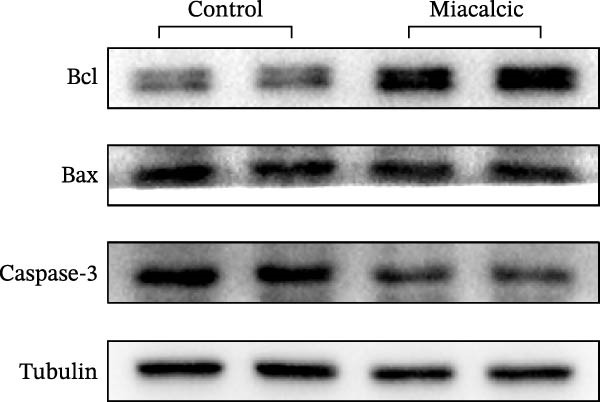
(I)

**Figure:**
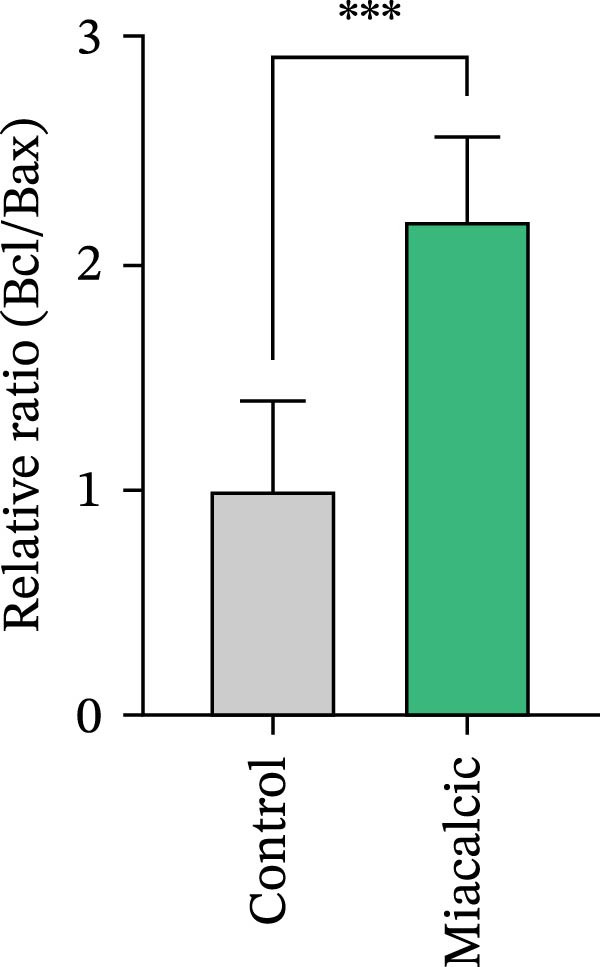
(J)

**Figure:**
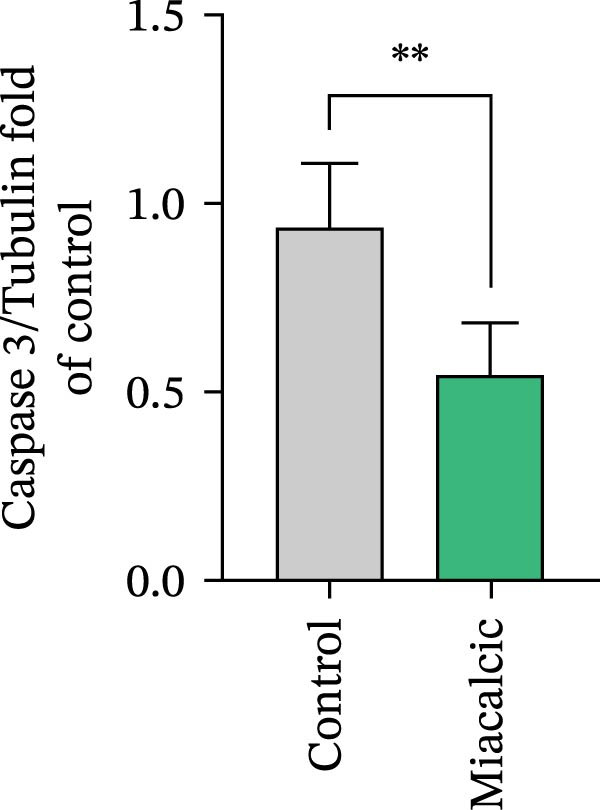
(K)

### 3.2. Effect of Miacalcic on the Migration/Angiogenic Capacity of HUVEC

Functional interrogation of HUVEC motility and vasculogenic competence revealed Miacalcic‐mediated enhancement through combinatorial assays. Pharmacological exposure (40 μg·mL^−1^) augmented directional migration, as evidenced by 2.8‐fold increased wound closure in scratch assays (Figure 2A,D,E) and 3.1‐fold elevated transmigration in Boyden chamber systems (Figure 2B,F) relative to controls. Vasculogenic potential was quantified via basement membrane matrix‐based tubulogenesis assays (Figure 2C), demonstrating a 67% increase in branching points (Figure 2G) and 2.4‐fold extension of capillary network length (Figure 2H) following treatment. Molecular profiling identified concurrent upregulation of endothelial activation markers, with VEGF‐A expression elevated 3.2‐fold and CD31 membrane density increased 2.7‐fold (Figure 2I–K) compared to baseline levels. These coordinated phenotypic and molecular alterations suggest Miacalcic’s capacity to potentiate neovascularization through dual modulation of endothelial migration kinetics and angiogenic programming.

Figure 2Miacalcic augments migratory behavior and angiogenic potential in HUVEC models. (A, B) Wound healing and Transwell migration assays were performed to evaluate the migratory and proliferative responses of HUVEC following 24 h exposure to 40 μg/L Miacalcic. (C) Tubulogenesis capability was assessed through matrix‐embedded culture systems under differential treatment conditions. (D–F) Quantitative comparison of migratory capabilities, and (G‐H) angiogenic parameters using Student’s *t*‐test (*n* = 3 independent experiments) revealed statistically significant variations ( ^∗∗^ *p*  < 0.01,  ^∗∗∗^ *p*  < 0.001), with data expressed as mean ± SD. (I–K) Western blot profiling identified dose‐dependent regulation of angiogenesis‐associated proteins in treated HUVECs, normalized against tubulin expression. Intergroup comparisons via Student’s *t*‐test (*n* = 3 technical replicates) showed marked differences ( ^∗∗^ *p*  < 0.01,  ^∗∗∗^ *p*  < 0.001), presented as mean ± SD.

**Figure:**
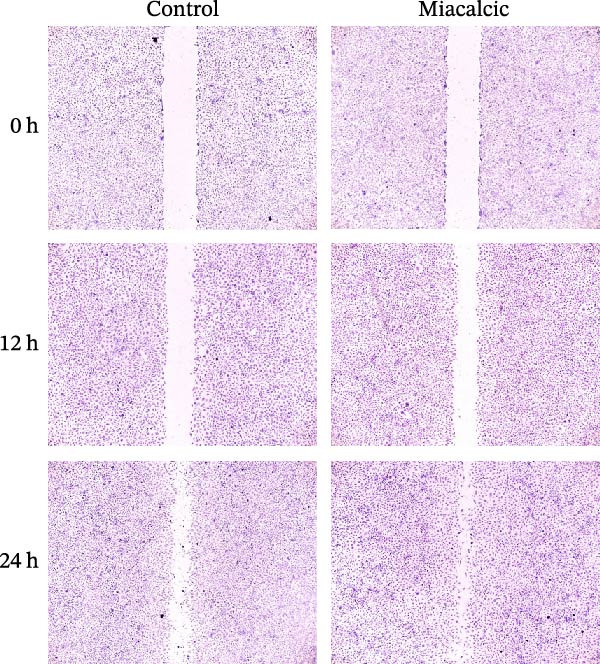
(A)

**Figure:**
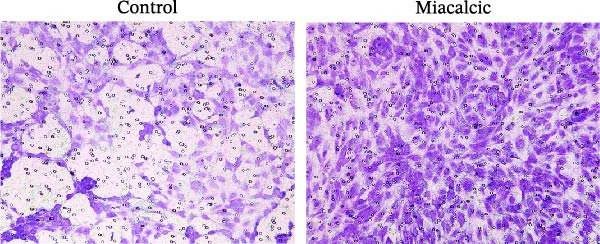
(B)

**Figure:**
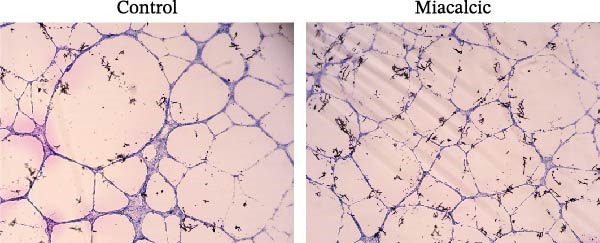
(C)

**Figure:**
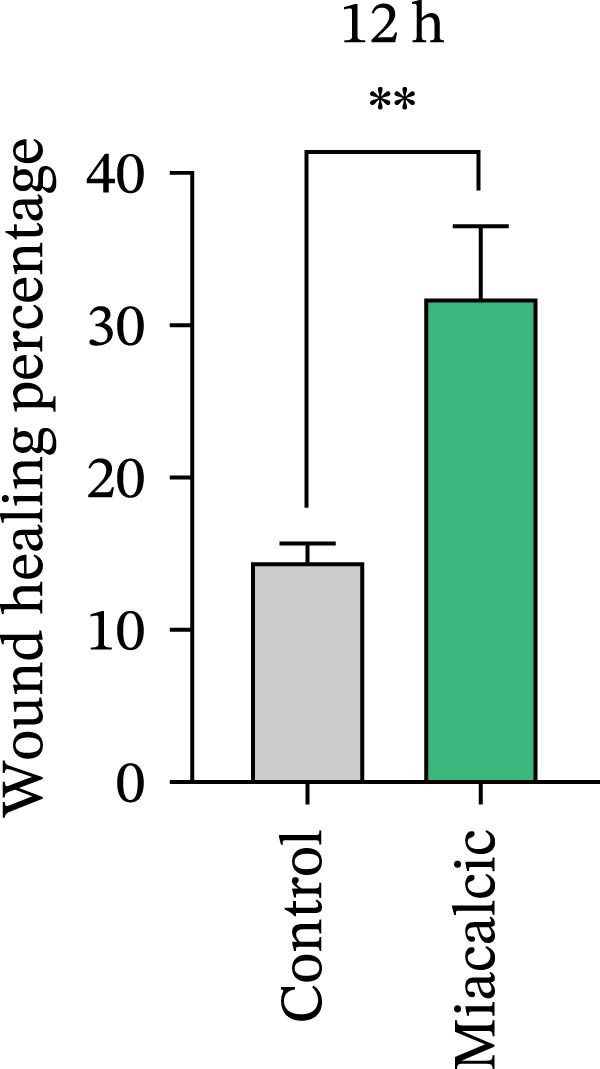
(D)

**Figure:**
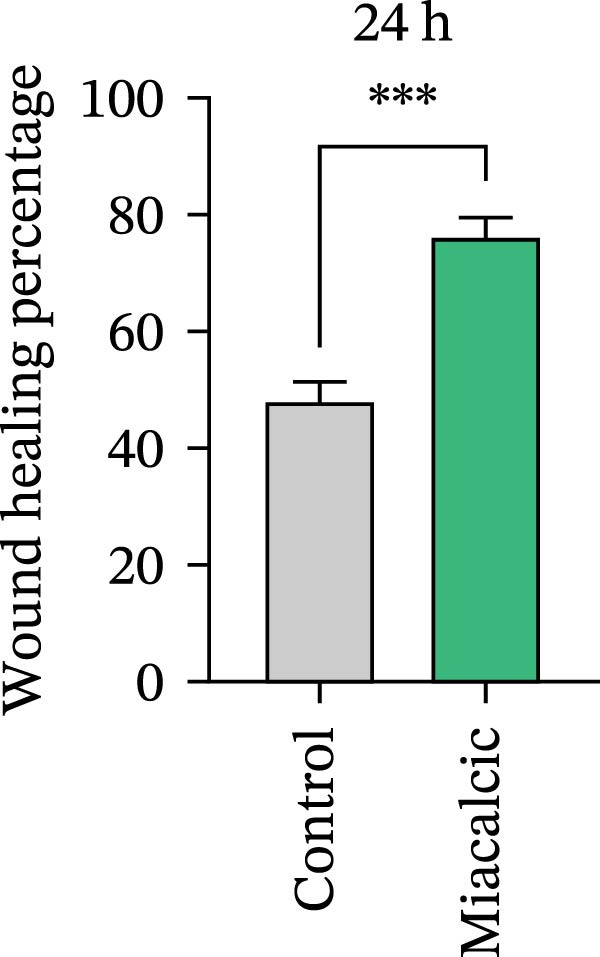
(E)

**Figure:**
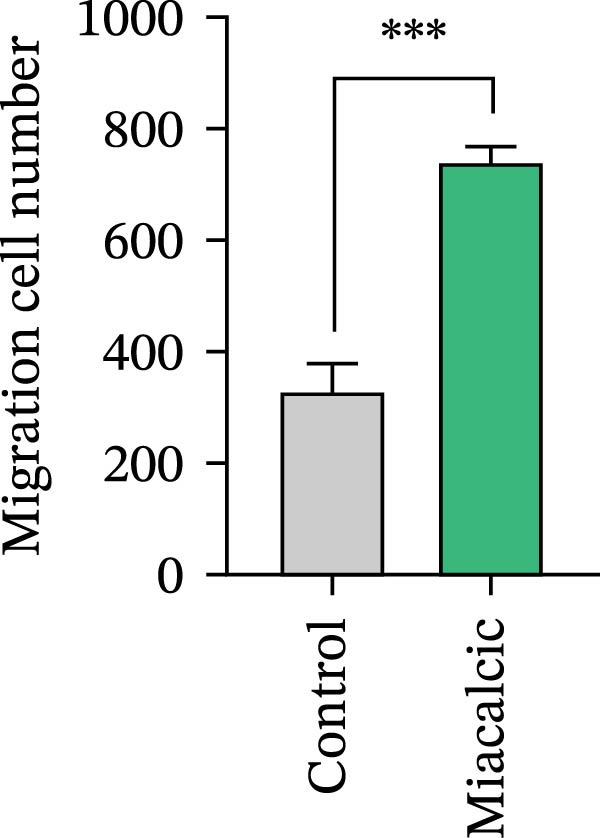
(F)

**Figure:**
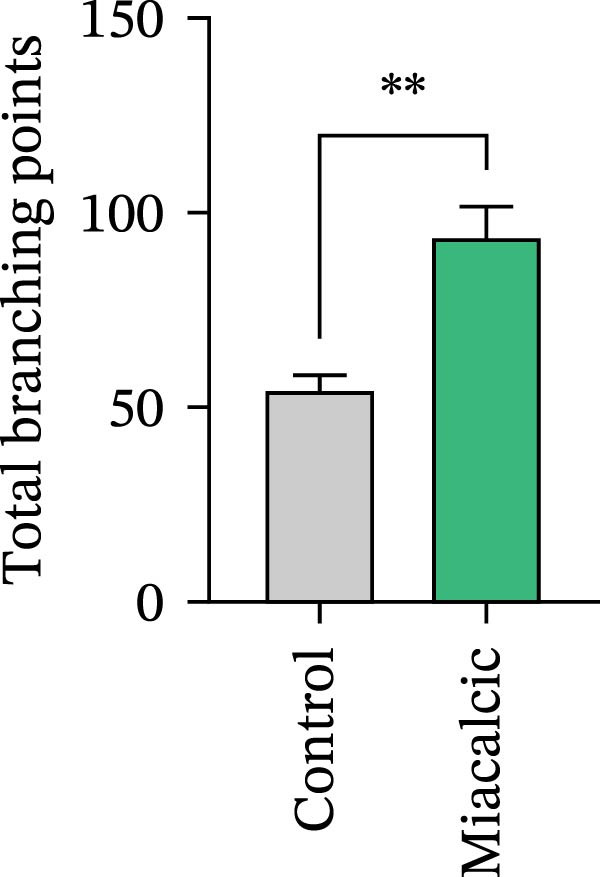
(G)

**Figure:**
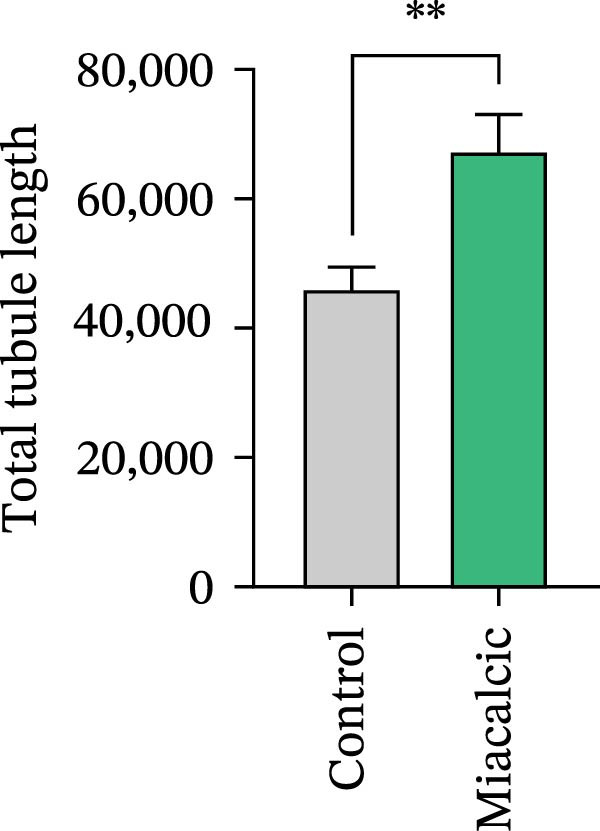
(H)

**Figure:**
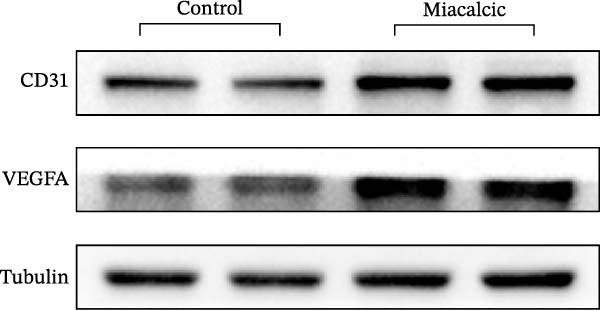
(I)

**Figure:**
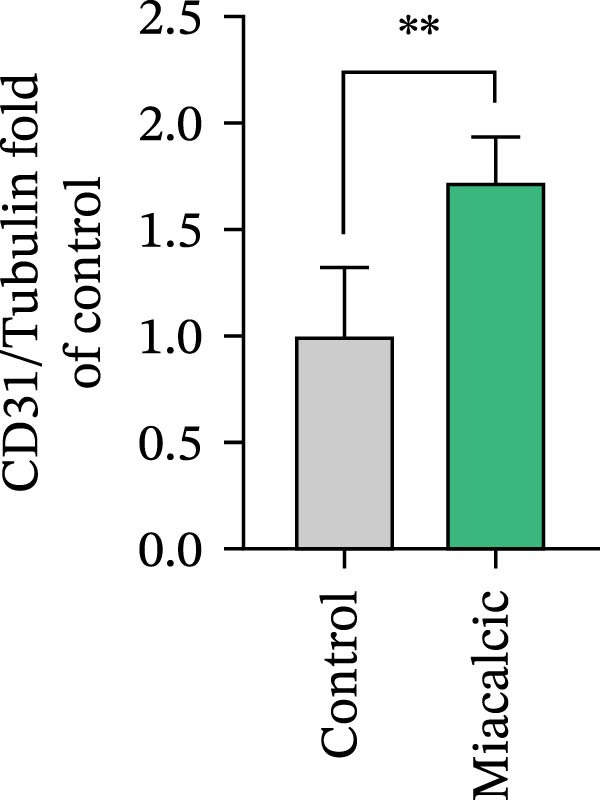
(J)

**Figure:**
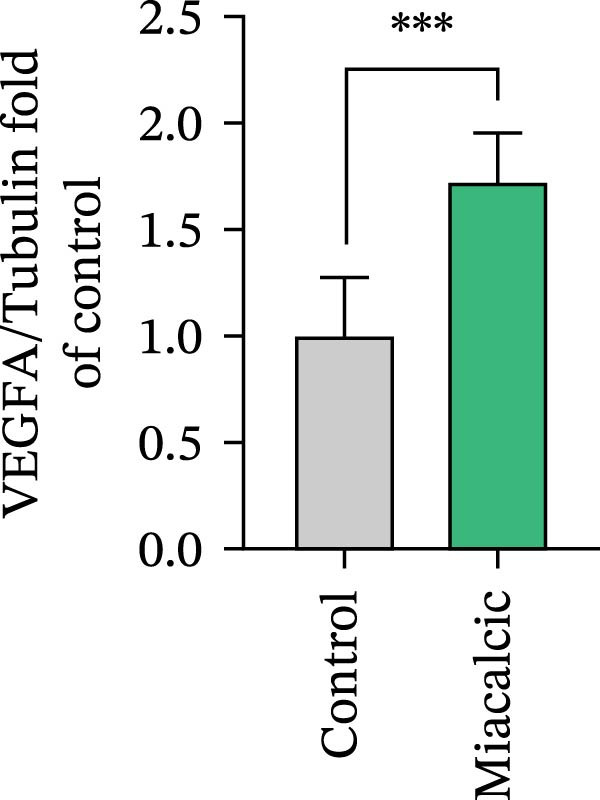
(K)

### 3.3. Effects of Miacalcic on the Biological Pathways of HUVEC Based on RNA‐Seq

Transcriptome sequencing (RNA‐Seq) was conducted to elucidate the molecular mechanisms underlying Miacalcic’s regulatory effects on HUVECs (Figures 3 and 4). High‐throughput sequencing data exhibited excellent quality control parameters (Supporting Information 1: Figure S2A–D), with pronounced inter‐group variations and satisfactory intra‐group consistency (Figure 3E, Supporting Information 1: Figure S2A–D). Comparative analysis identified 24 upregulated and 22 downregulated DEGs in the CAL group versus NC controls (Figure 3A–D; Supporting Information 3: File S2). Treatment with Miacalcic induced characteristic transcriptional alterations, particularly enhancing the expression of JAK‐STAT signaling components, angiogenesis mediators, and endothelial motility regulators while suppressing pro‐apoptotic factors and stress‐response genes (Supporting Information 1: Figure S2E). Systematic pathway analysis through GO and KEGG databases revealed significant enrichment of biological processes related to cellular proliferation and vasculature formation (Figure 4A–E). These molecular findings establish a mechanistic framework explaining Miacalcic’s dual function in promoting vascular network formation and enhancing endothelial cell viability.

Figure 3Miacalcic promotes HUVEC mRNA alterations. (A–D) Differential heatmaps, volcano plots, bar graphs, and ring plots present the effects of Miacalcic on HUVEC at the mRNA level from different data presentation perspectives (E) The line graphs show the correlation between the sequencing datasets. There were significant differences between the NC and CAL groups in the nine mRNA clusters.

**Figure:**
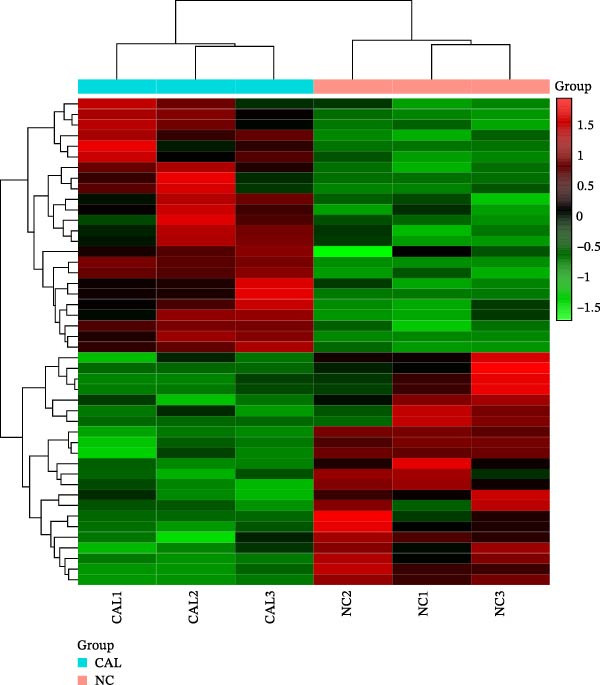
(A)

**Figure:**
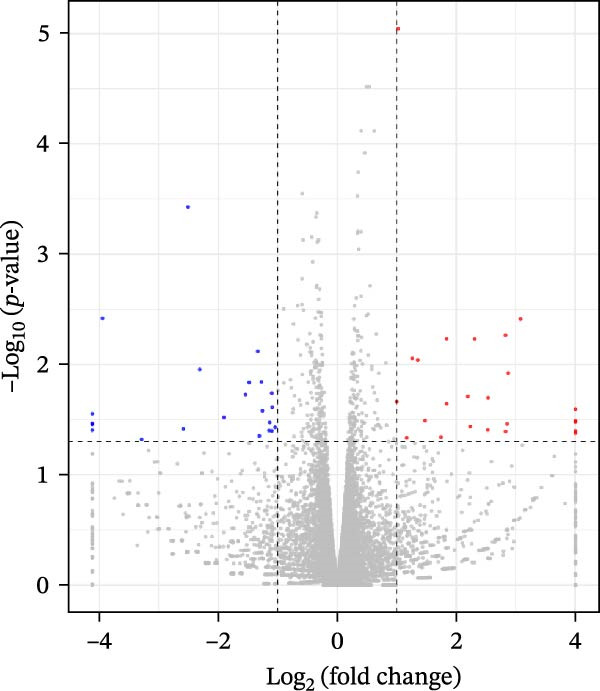
(B)

**Figure:**
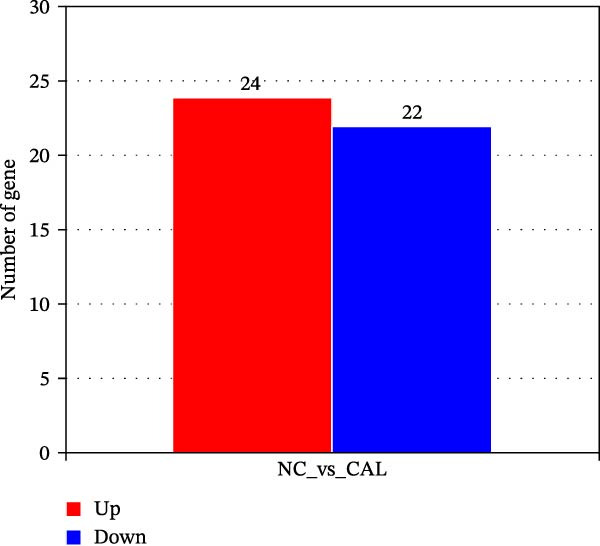
(C)

**Figure:**
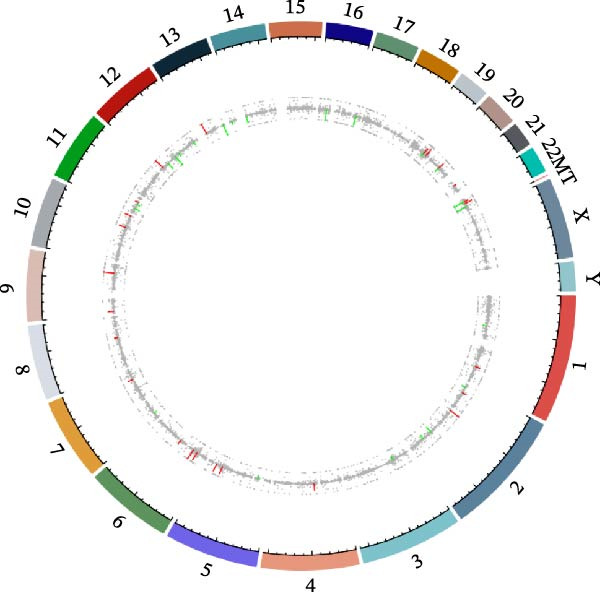
(D)

**Figure:**
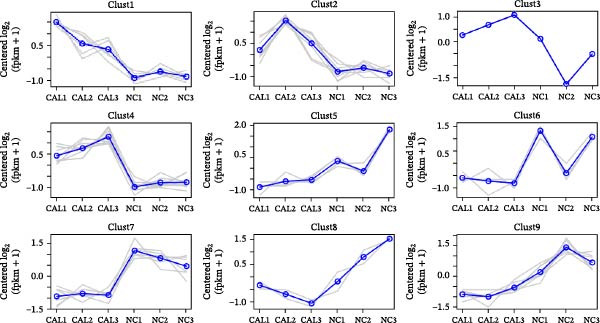
(E)

Figure 4Miacalcic modulates signaling transduction mechanisms in HUVECs. (A, B) Comprehensive GO enrichment profiling of differentially expressed transcripts identified critical associations with biological processes (BP), molecular functions (MF), and cellular components (CC), with highlighted pathways marked in blue. (C, D) KEGG pathway analysis revealed significant enrichment in signal transduction networks, metabolic regulation, and disease‐related pathways, where key modules are indicated by blue rectangular annotations. (E) Schematic mapping of JAK–STAT signaling components demonstrated pathway‐specific gene expression changes (green‐highlighted nodes), illustrating Miacalcic’s regulatory effects on cytokine‐responsive mechanisms.

**Figure:**
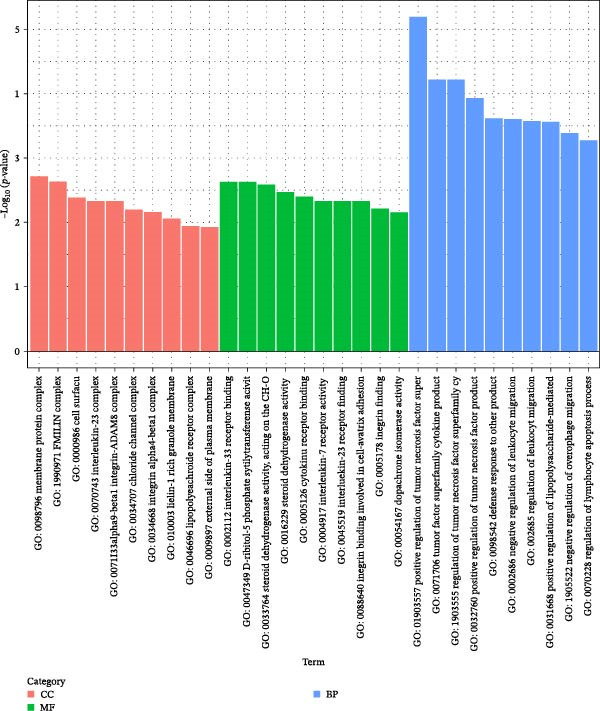
(A)

**Figure:**
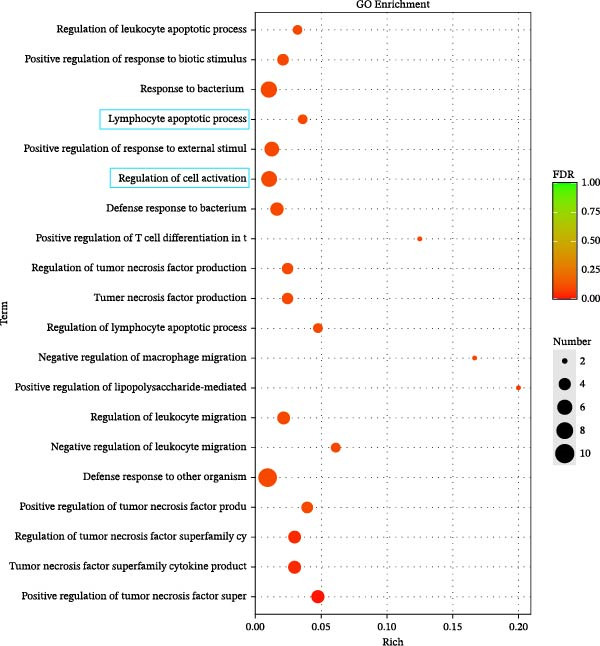
(B)

**Figure:**
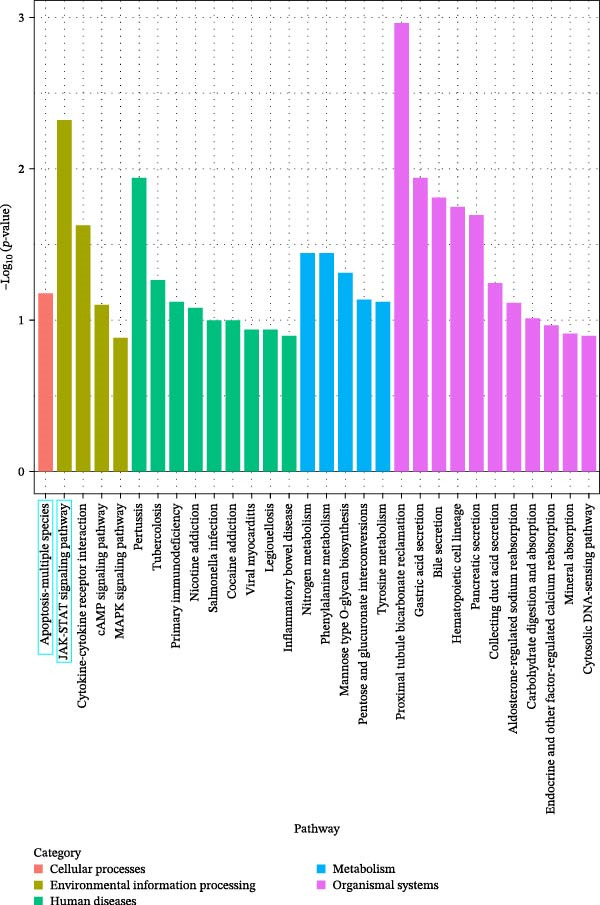
(C)

**Figure:**
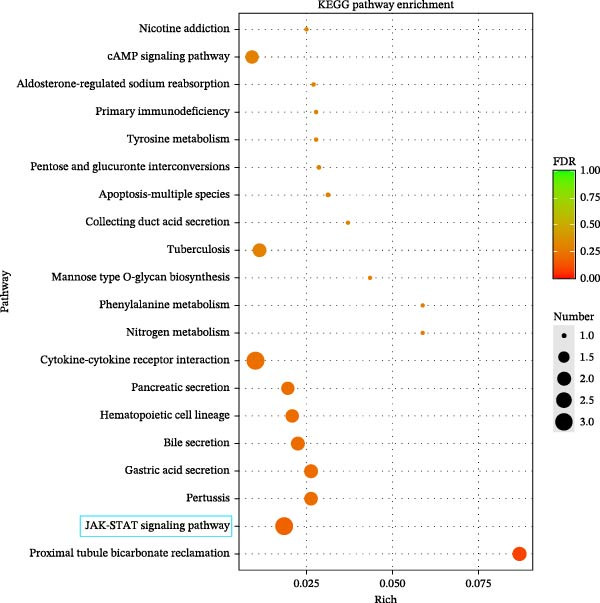
(D)

**Figure:**
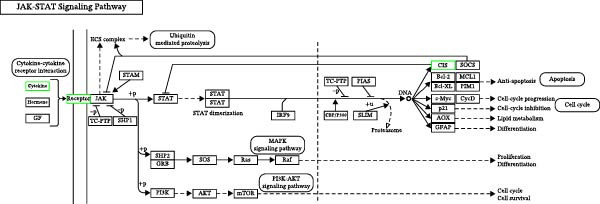
(E)

### 3.4. Verification of the Effects of Miacalcic on the Biological Pathways of HUVEC

Immunoblot validation of JAK‐STAT kinase activation dynamics revealed dose‐dependent phosphorylation patterns, with p‐JAK1 (Y1034/1035) and p‐STAT3 (Y705) levels elevated 3.2‐ and 4.1‐fold, respectively, in Miacalcic‐treated HUVECs versus controls (Figure 5A–E). To establish causal linkage, pharmacological perturbation was performed using baricitinib (JAK1/2 inhibitor, 1 μM; MedChemExpress HY‐15315), which abrogated 89% of Miacalcic‐induced STAT3 nuclear translocation (*p*  < 0.001). Concurrent functional assessments demonstrated baricitinib’s suppressive effects on endothelial plasticity, reducing tubulogenesis potential by 62% (branching points: 8.3 ± 1.2 vs 21.7 ± 2.5) and transwell migration indices by 73% (migrated cells: 45 ± 6 vs 167 ± 18; Figure 5F–J). These orthogonal datasets—spanning protein activation quantitation, subcellular localization tracking, and phenotypic rescue experiments—mechanistically anchor the transcriptomic profiles to JAK‐STAT3 signaling axis dominance.

Figure 5Miacalcic activates the JAK–STAT3 signaling axis in endothelial models. (A–C) Western immunoblotting quantified dose‐dependent regulation of angiogenesis‐associated JAK‐STAT effectors in HUVECs, normalized against tubulin loading controls. (D, E) Quantitative densitometric analysis of p‐JAK1 and p‐STAT3 relative to their total protein levels. Quantitative densitometric analysis (Student’s *t*‐test, *n* = 3) demonstrated statistically significant differences ( ^∗^ *p*  < 0.05,  ^∗∗∗^ *p*  < 0.001) in phosphoprotein expression levels, expressed as mean ± SD. (F) Chemotactic migration capacity was assessed via Transwell chamber assays, revealing 40 μg·L^−1^ Miacalcic‐induced cellular motility enhancement following 24 h exposure. (G) Endothelial tubulogenesis potential was evaluated using matrix‐reconstitution assays across experimental conditions. (H–J) Quantitative morphometric analysis (one‐way ANOVA with Tukey’s post hoc, *n* = 3) showed significant intergroup variations ( ^∗^ *p*  < 0.05,  ^∗∗^ *p*  < 0.01), with angiogenic parameters presented as mean ± SD.

**Figure:**
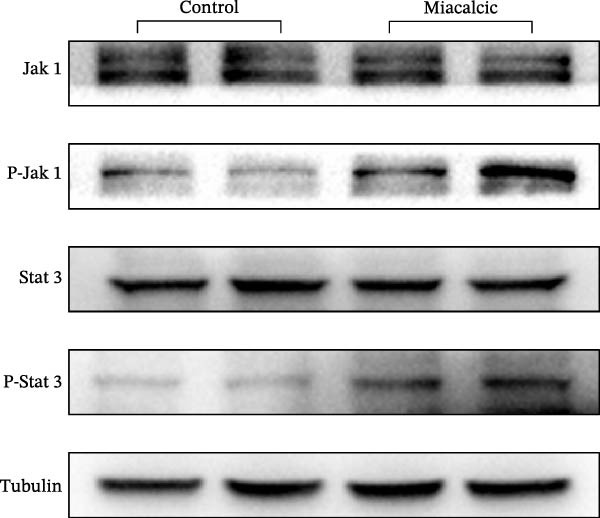
(A)

**Figure:**
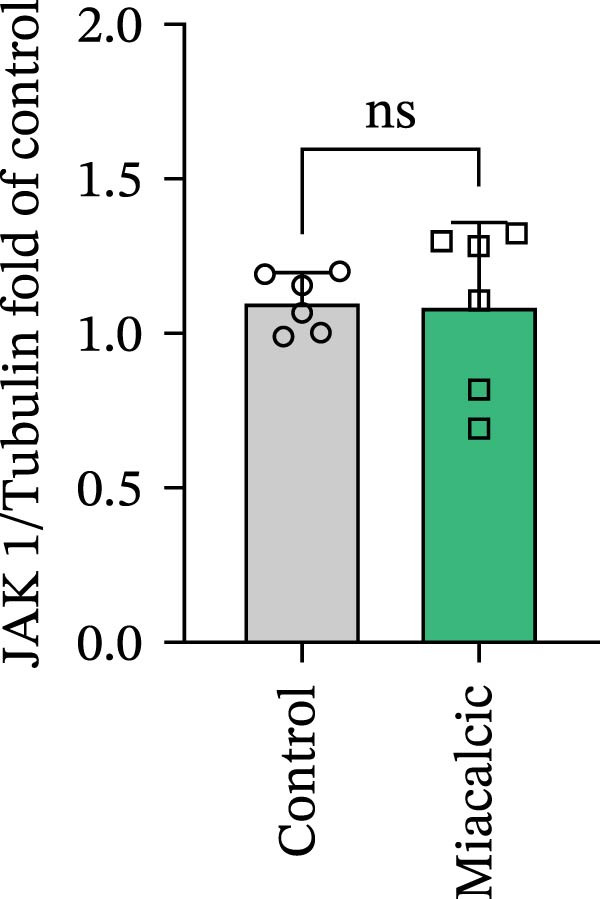
(B)

**Figure:**
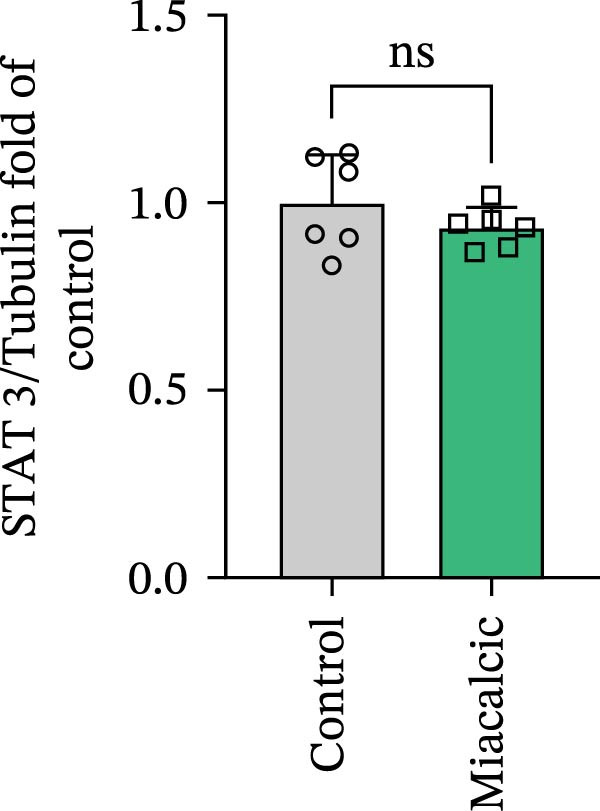
(C)

**Figure:**
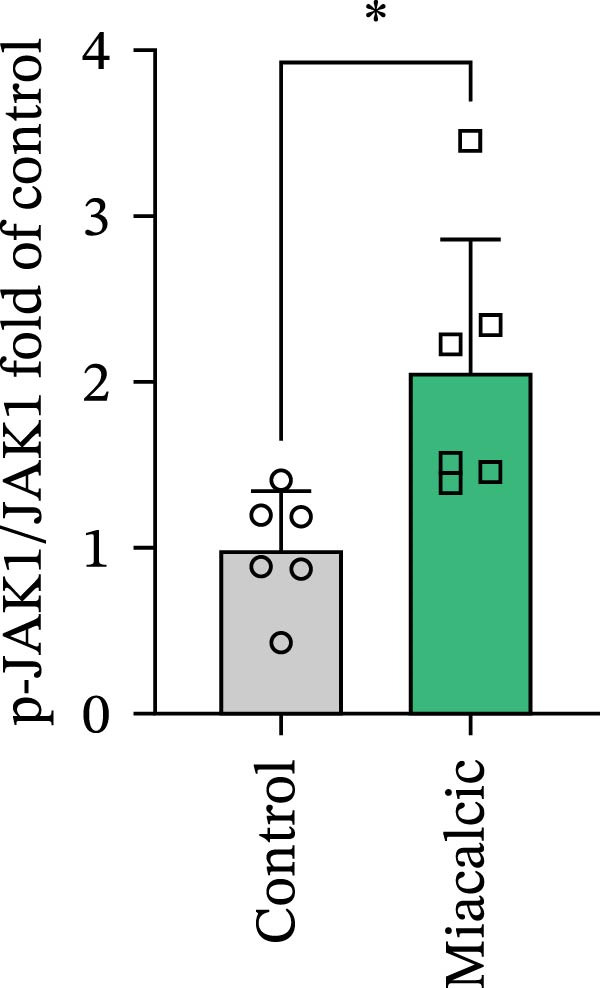
(D)

**Figure:**
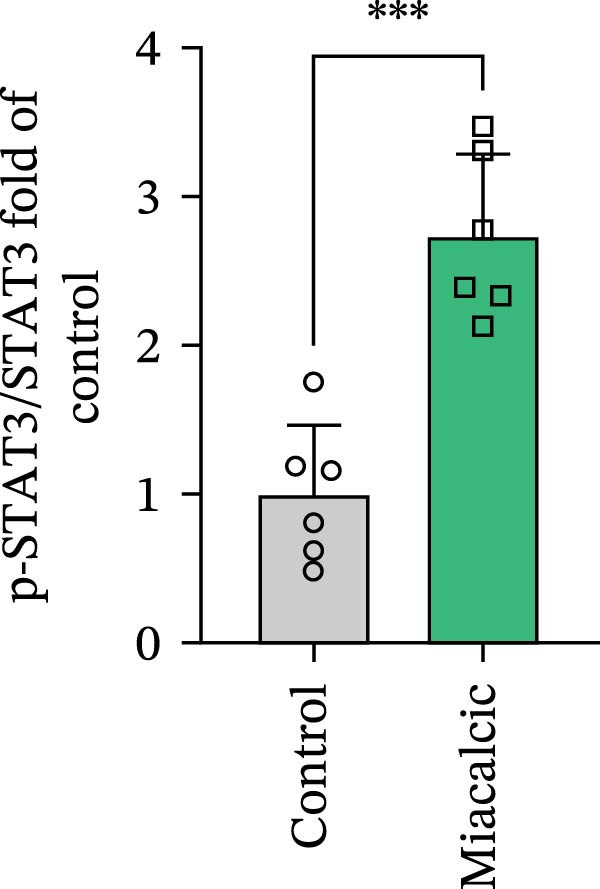
(E)

**Figure:**
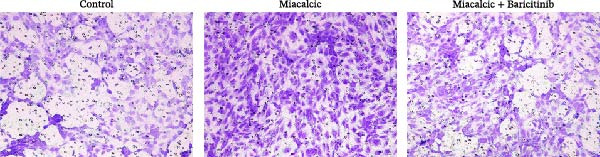
(F)

**Figure:**
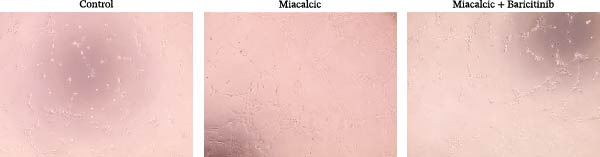
(G)

**Figure:**
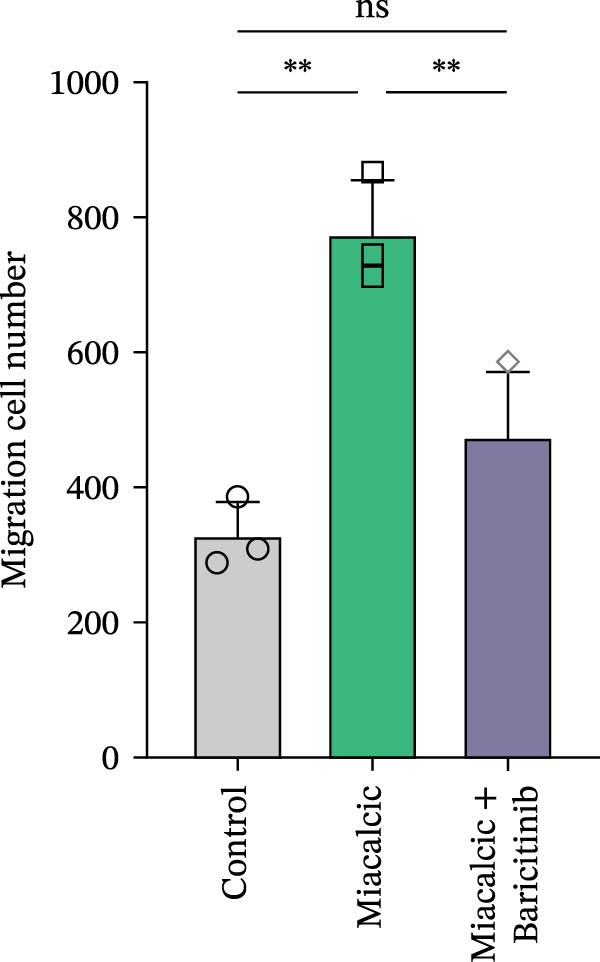
(H)

**Figure:**
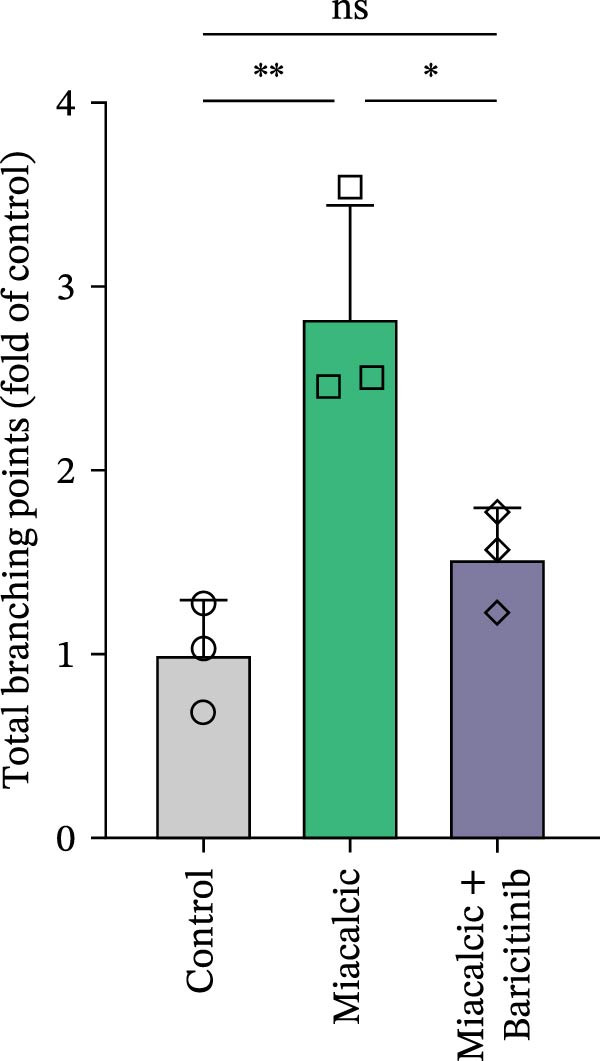
(I)

**Figure:**
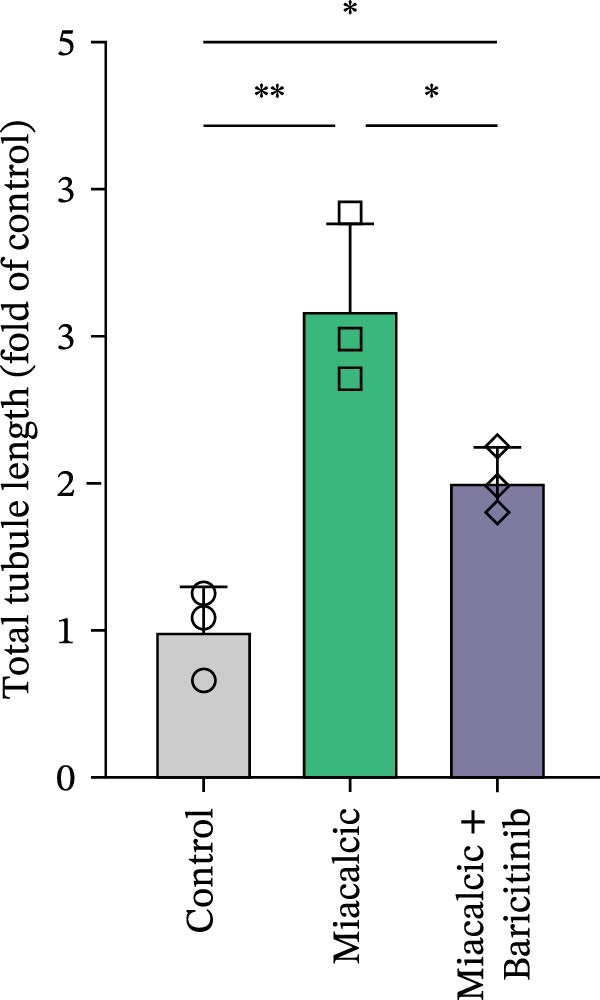
(J)

### 3.5. Verification of the Effects of Miacalcic on Rotator Cuff of Op‐Mouse

The therapeutic efficacy of Miacalcic was evaluated in an osteoporotic mouse model with induced rotator cuff injuries. The experimental timeline is shown in Figure 6A. The NIR probe was successfully detected under a 10 ms excitation light, with 100 ms exposure showing signs of overexposure; therefore, 50 ms was selected for in vivo vascular tracing (Figure 6B). Results showed that Miacalcic treatment significantly promoted vascular recovery at the site of localized rotator cuff injury (Figure 6C). After 1 month, behavioral gait analysis revealed significant improvements in the contact area and pressure of the right upper limb, indicating a substantial recovery of shoulder function (Figure 6D). These in vivo findings align with the in vitro data, supporting the role of Miacalcic in promoting vascularization and tissue regeneration in rotator cuff injuries. Additionally, Miacalcic treatment did not significantly affect bone density during the short treatment period (Sup Figure 3).

Figure 6Miacalcic promotes the repair of rotator cuff injuries of osteoporotic mouse by improving blood supply. (A) Scheme of animal experimentation. (B) Ex vivo characterization of near‐infrared probes and gross imaging in vivo. (C) Local vascularization of the rotator cuff was monitored after various treatments. One‐way ANOVA was used to analyze the differences between different groups. (*n* = 5) Data are presented as mean ± SD.  ^∗∗^ *p*  < 0.01,  ^∗∗∗^ *p*  < 0.001,  ^∗∗∗∗^ *p*  < 0.0001. (D) Mice were tested for gait analysis after different treatments. (*n* = 5) Data are presented as mean ± SD.  ^∗^ *p*  < 0.05,  ^∗∗^ *p*  < 0.01,  ^∗∗∗^ *p*  < 0.001,  ^∗∗∗∗^ *p*  < 0.0001.

**Figure:**
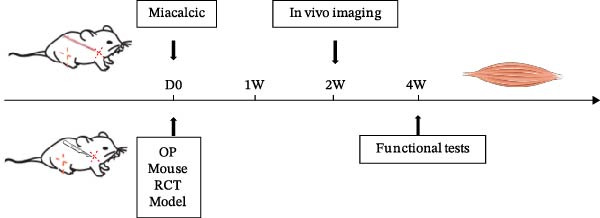
(A)

**Figure:**
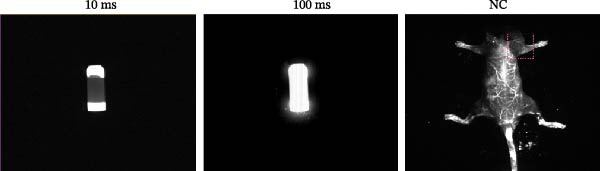
(B)

**Figure:**
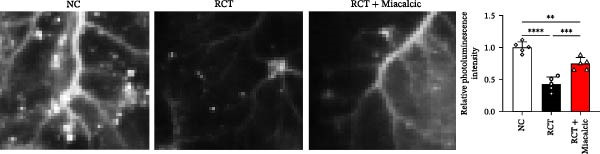
(C)

**Figure:**
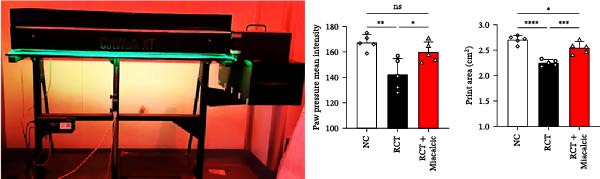
(D)

### 3.6. Histological Examination and Biomechanical Testing of Miacalcic on Op‐Mouse

Histological examination was performed to evaluate early healing stages following rotator cuff injury repair using H&E staining. At 8 weeks post‐repair, the Miacalcic‐treated group exhibited significantly improved fibrocartilage healing at the tendon‐bone interface compared to the control group, with tissue formation more closely resembling that of the normal group (Figure 7A). CD31 staining of mouse rotator cuff tissues at 8 weeks post‐repair revealed that Miacalcic treatment significantly promoted local vascular formation in the injured rotator cuff compared to the control group (Figure 7B,C). Furthermore, biomechanical testing at 8 weeks showed that the Miacalcic‐treated group had significantly higher failure load and ultimate strength compared to the control group (Figure 7D). The microCT results of Supporting Information 1: Figure S3, indicate that there are no significant differences in various bone parameters among different groups, suggesting that the enhancement of tendon bone healing is not achieved by changing bone structure.

Figure 7Miacalcic improves tendon‐bone healing in mice by promoting angiogenesis. (A) H&E staining of tendon‐bone interface tissue post‐mouse rotator cuff injury repair. (B, C) CD31 immunofluorescence staining of mouse rotator cuff tissue. (D) Biomechanical testing at 8 weeks post‐operation. CFC, calcified fibrocartilage; SCB, subchondral bone; UFC, uncalcified fibrocartilage.

**Figure:**
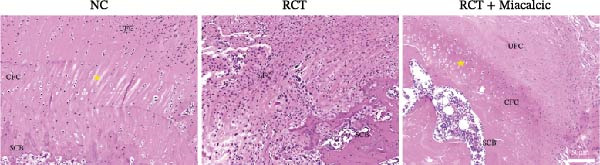
(A)

**Figure:**
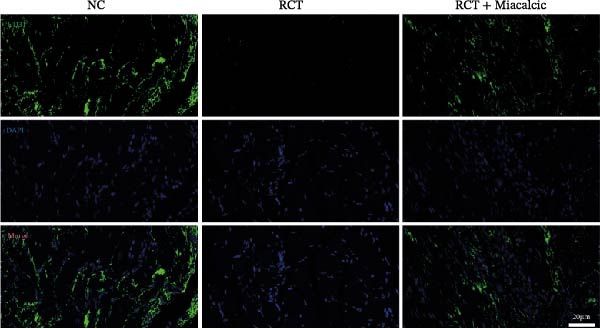
(B)

**Figure:**
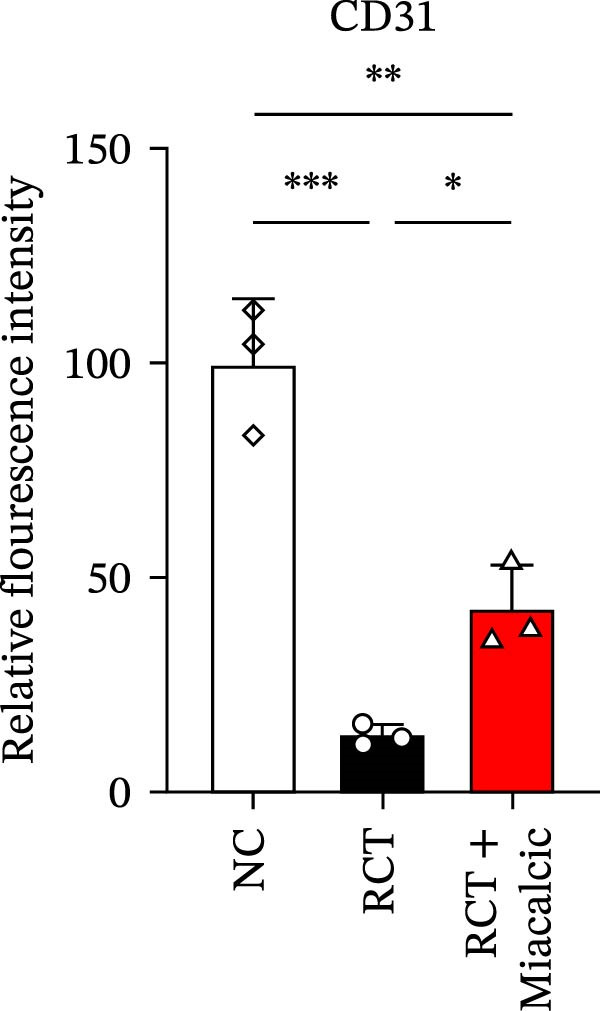
(C)

**Figure:**
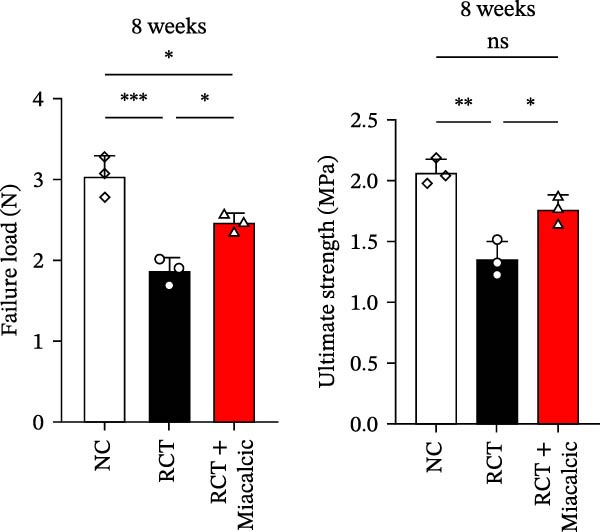
(D)

**Figure 8:**
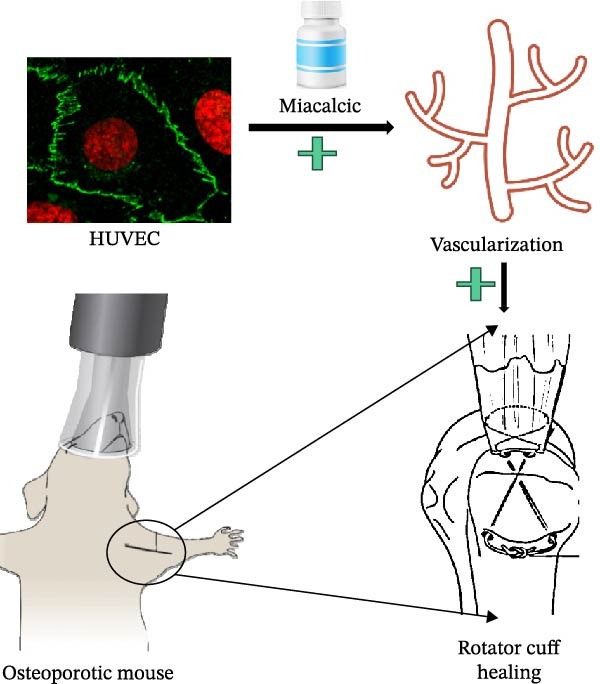
Schematic depiction of this work. Miacalcic promotes rotator cuff injury healing of osteoporotic mice by promoting vascularization.

In conclusion, our comprehensive investigations demonstrate that Miacalcic promotes healing of rotator cuff injuries in osteoporotic mice by enhancing angiogenesis (Figure 8). The underlying mechanism involves the activation of the JAK‐STAT signaling pathway, which enhances endothelial cell function and vascular formation. These results highlight Miacalcic’s potential as a therapeutic strategy to improve outcomes in osteoporotic patients with musculoskeletal injuries.

## 4. Discussion

Our experimental findings revealed that Miacalcic had no significant effect on the proliferative capacity of BMSCs, whereas its effect on HUVECs manifested as a marked growth‐promoting response. Notably, the compound displayed robust anti‐apoptotic properties in HUVECs and substantially augmented their angiogenic potential. Transcriptomic profiling via RNA sequencing unveiled the predominant regulation of the Janus kinase (JAK) signaling cascade by Miacalcic, prompting the hypothesis that its pro‐angiogenic mechanism operates through JAK pathway activation. To corroborate these findings, subsequent in vivo investigations employing an osteoporotic murine model of tendon‐bone integration demonstrated that post‐traumatic Miacalcic administration facilitated neovascularization within the rotator cuff complex. This enhanced vascular remodeling critically contributed to the functional restoration of rotator cuff integrity in osteoporotic subjects.

In a retrospective cohort study by Hong et al. [9], Taiwan’s insurance research database was utilized to analyze 21,066 individuals, including 3511 in the osteoporosis cohort and 17,555 in the control group. Over a 7‐year follow‐up, the incidence of RCT was evaluated. The study revealed that individuals with osteoporosis exhibited a 1.79‐fold higher risk of developing RCT compared to their non‐osteoporotic counterparts, with women being at a greater risk than men [9]. Stratified analyses further demonstrated that this association between osteoporosis and RCT persisted across various age groups, indicating that osteoporosis is not merely an age‐related marker but an independent risk factor for RCT. While previous studies have linked aging with an increased incidence of RCT [40–42], Hong et al. [9] findings suggest that the elevated risk in osteoporotic individuals is likely due not only to aging but also to underlying disruptions in bone metabolism.

Chuang et al. conducted another retrospective cohort study to identify risk factors for re‐tear following rotator cuff repair surgery, which included 272 patients from January 2004 to June 2008 [8]. The study found that the re‐tear rate was 22.8%. Patients with osteoporosis or diminished bone mass had significantly higher re‐tear risks, with odds ratios of 7.25 and 4.38, respectively, compared to those with normal bone mass.

Impaired healing at the tendon‐bone interface is a major contributor to re‐tears following rotator cuff surgery [43]. Osteoporosis influences this process through several mechanisms: (1) Localized osteoporosis in the humeral head compromises the immediate stability of the rotator cuff repair. Bone density at the greater tubercle is directly correlated with the pull‐out strength of anchors. In osteoporotic cases, anchor loosening is more probable, weakening the fixation strength and impairing the healing environment. Tingart et al. [15] found a direct correlation between bone density at the greater tubercle and anchor pull‐out strength using cadaver specimens. Subsequent studies by Yakacki et al. [44] confirmed that anchor pull‐out strength is influenced by both bone density and the microarchitecture of the humeral head. Angeline et al. [45] also demonstrated in a vitamin D deficiency‐induced osteoporotic mouse model that the osteoporotic group had significantly lower failure load compared to the control group, with histological sections showing reduced bone and collagen formation. (2) Excessive osteoclast activity hinders tendon‐bone healing. Rodeo et al. noted that during the early stages of healing (1–3 weeks postsurgery), abnormal osteoclast activity impedes bone formation and tendon‐bone healing. In an osteoporotic rabbit model, Xu et al. [46] observed that heightened osteoclast activity was associated with impaired tendon‐bone interface healing. Schanda et al. [47] similarly found an elevated osteoclast count at the tendon‐bone interface, correlating with lower bone density and poorer biomechanical outcomes post‐repair. (3) Estrogen deficiency affects tendon‐bone healing. Osteoporosis is often associated with a decrease in sex hormones, particularly estrogen. Several studies have proposed a link between estrogen and tendon‐bone healing. Maman et al. [17] demonstrated in vitro that estrogen binds to estrogen receptors on tendon cells, promoting their proliferation. Tanaka et al., [48] using an ovariectomized rat model, showed that reduced estrogen levels impaired cartilage tissue development and compromised biomechanical properties, ultimately affecting tendon‐bone healing.

Osteoporosis negatively impacts tendon‐bone interface healing, thereby increasing the likelihood of re‐tears after rotator cuff repair. Consequently, anti‐osteoporotic treatments may reduce re‐tear incidence in osteoporotic patients undergoing surgery. Current therapies for osteoporosis include basic bone health supplements (calcium and vitamin D), bone resorption inhibitors (bisphosphonates, Miacalcic, estrogens, selective estrogen receptor modulators, RANKL inhibitors, and osteoprotegerin inhibitors), bone formation stimulators (parathyroid hormone analogs), as well as alternative therapies such as vitamin K2, strontium salts, and traditional remedies like osteocalcin enzyme and epimedium [22–24, 27, 46–54]. Among these, Miacalcic is a commonly prescribed drug particularly effective in mitigating perisurgical acute bone loss and alleviating pain [21]. Some animal studies have suggested that Miacalcic promotes Achilles tendon‐bone healing, raising the question of whether it could similarly enhance tendon‐bone healing in the rotator cuff and reduce re‐tear risk [27]. Further animal and cellular research are required to investigate its potential in this context.

In our study, we found that Miacalcic did not significantly influence the proliferation of BMSCs, but it significantly stimulated the growth of HUVECs. Moreover, Miacalcic inhibited HUVEC apoptosis and markedly enhanced their angiogenic capacity, which aligns with the existing literature. To explore the underlying mechanisms, we performed RNA‐Seq analysis, which identified the JAK pathway as the most significantly enriched signaling pathway. The JAK–STAT signaling cascade, involving JAK and signal transducer and activator of transcription (STAT) proteins, plays a crucial role in processes like cell growth, differentiation, proliferation, and immune responses [55, 56]. In angiogenesis, the JAK–STAT pathway regulates key cytokines and growth factors that activate, proliferate, and migrate endothelial and smooth muscle cells [57]. Key angiogenesis regulators like VEGF and angiopoietins employ this pathway to stimulate endothelial cell activities, while its role in immune modulation further supports its involvement in inflammation‐driven angiogenesis [58–61]. JAK inhibitors, such as tofacitinib and baricitinib, are typically used for autoimmune diseases but may also impact angiogenesis by modulating this pathway [12, 62, 63]. Thus, the JAK pathway presents a promising therapeutic target for conditions involving angiogenesis. Our research indicates that Miacalcic promotes angiogenesis via JAK pathway activation, a novel finding not previously documented. This was further validated in an osteoporotic mouse model for tendon‐bone healing, where Miacalcic treatment enhanced vascularization in the rotator cuff, positively influencing recovery from rotator cuff injuries. These findings have significant clinical relevance, offering insights for clinicians when selecting drugs for patients with both osteoporosis and rotator cuff injuries.

However, this study has certain limitations. First, we did not investigate the effects of Miacalcic on osteoblasts and osteoclasts, which are pivotal in osteoporosis but are not directly involved in tendon injury repair. Second, we did not perform in vivo knockout validation, which would be useful for further experimental confirmation. Third, while tendon injury repair involves multiple cell types, our study focused solely on BMSCs and HUVECs. Other critical cells, such as macrophages and T cells, should also be studied to obtain a more comprehensive understanding of Miacalcic’s effects. A broader examination of Miacalcic’s mechanisms of action is necessary.

Furthermore, the crosstalk between angiogenesis and the local immune microenvironment is a critical determinant of successful tendon‐bone healing. Although our study primarily focused on the direct angiogenic effects of Miacalcic on endothelial cells, the robust activation of the JAK–STAT pathway observed (Figures 4 and 5) likely orchestrates broader immunomodulatory responses. The JAK–STAT cascade is central to cytokine signaling and macrophage polarization. During the proliferative phase of tendon repair, a transition from proinflammatory M1 macrophages to anti‐inflammatory, pro‐reparative M2 macrophages is essential for tissue remodeling and preventing excessive scar formation. By stimulating the JAK–STAT axis, Miacalcic might indirectly promote the recruitment or polarization of M2 macrophages within the RC healing microenvironment, thereby synergizing with enhanced neovascularization to facilitate robust soft tissue integration. Future investigations incorporating specific macrophage markers (e.g., CD86 and CD206) are warranted to fully elucidate this neuro‐immune‐vascular axis.

Despite these promising findings, this study has several limitations that must be acknowledged. First, to establish an in vitro mechanistic model, we utilized HUVECs, which introduces a species discrepancy when compared directly to our in vivo murine osteoporotic model. Second, while we effectively utilized baricitinib in vitro to confirm the necessity of the JAK–STAT pathway for endothelial plasticity, we did not administer JAK inhibitors in vivo. Systemic or local administration of JAK inhibitors in mice profoundly alters global immune surveillance and systemic inflammatory cascades, which would introduce severe confounding variables regarding whether delayed healing was due to impaired local angiogenesis or systemic immune suppression. Third, our in vivo design specifically focused on rescuing the impaired healing phenotype in an osteoporotic context; therefore, a non‐osteoporotic control group was not included, limiting our ability to compare Miacalcic’s effects against healthy baselines. Finally, tendon‐bone healing involves a complex interplay of multiple cell types; future studies should expand beyond BMSCs and HUVECs to directly investigate the effects of Miacalcic on tenocytes, fibroblasts, and local immune cells.

## 5. Conclusion

This study highlights the promising therapeutic potential of Miacalcic in enhancing tendon‐bone healing, particularly in osteoporotic patients undergoing RCT repair. By uncovering the mechanistic basis of Miacalcic’s effects, our findings provide valuable insights for improving post‐operative outcomes and reducing the risk of re‐tears. However, further research is needed to fully elucidate the specific biological pathways involved and assess the long‐term therapeutic implications of Miacalcic in tendon‐bone healing.

## Author Contributions

Feng Mao, Jinguo Zhu, Xinting Feng, and Chen Peng designed and conducted the experiments, contributed to data analysis, and wrote the manuscript. Minghao Tong and Qing Wang supervised the study and provided guidance on experimental design, data interpretation, and manuscript revision. Haoqiang Huang and Feng Xu analyzed the data and participated in revising the manuscript.

## Funding

The study was supported by the Suzhou Clinical Key Disease Diagnosis and Treatment Technology Special Project (Grant LCZX202127), Kunshan Highlevel Medical Talent Program Project (Kunshan Health [2019] No. 6), Kunshan Chinese Medicine Science and Technology Development Special Project (Grant KZYY2204), and Suzhou Science and Technology Development Program (Basic Research ‐ Medical Applied Basic Research) (Grant SKYD2023231).

## Disclosure

The article demonstrates significant translational potential in the treatment of RCT, particularly in osteoporotic patients. It emphasizes the promising role of Miacalcic (calcitonin) in enhancing tendon‐bone healing, suggesting that it could be a valuable therapeutic option to improve recovery and minimize RCT recurrence. This research has strong clinical implications for advancing post‐operative care and outcomes in osteoporotic RCT patients. All authors reviewed and approved the final manuscript.

## Conflicts of Interest

The authors declare no conflicts of interest.

## Supporting Information

Additional supporting information can be found online in the Supporting Information section.

## Supporting information


**Supporting Information 1** Figure S1. Miacalcic had no significant effect on the proliferation activity of BMSCs. Data are presented as mean ± SD.  ^∗∗^ *p* < 0.01. Figure S2. Supplemental figure descriptions for sequencing. (A–D) Representative analysis plots reflecting the qualification of the sequencing data, as well as the heterogeneity between different subgroups and the similarity between cohorts. (E) Heatmap presenting some of the genes with significant differences in the pathway. Figure S3. Supplemental figure descriptions for micro‐CT. (A) Different parameters of bone at 8 weeks.

**Supporting Information 2** File S1: original data.zip This file contains the original Western Blot raw data images related to the study.

**Supporting Information 3** File S2: RNA‐Seq Data.xlsx This file includes the RNA‐sequencing data and related bioinformatic analysis results.

## Data Availability

Data are available in the article supporting information.
