# Supplementary material for: Miacalcic Enhances Rotator Cuff Injury Healing in Osteoporotic Mice by Stimulating Neovascularization via the JAK Pathway
Source: Mediators Inflamm. 2026 Jun 3;2026:7332100. doi: 10.1155/mi/7332100 (PMC13239275; doi:10.1155/mi/7332100)
Supplement: Supplementary file 1 — Supporting Information 1 Figure S1. Miacalcic had no significant effect on the proliferation activity of BMSCs. Data are presented as mean ± SD. ∗∗ p < 0.01. Figure S2. Supplemental figure descriptions for sequencing. (A–D) Representative analysis plots reflecting the qualification of the sequencing data, as well as the heterogeneity between different subgroups and the similarity between cohorts. (E) Heatmap presenting some of the genes with significant differences in the pathway. Figure S3. Supplemental figure descriptions for micro‐CT. (A) Different parameters of bone at 8 weeks. [file MI-2026-7332100-s001.docx]

**Supplemental files
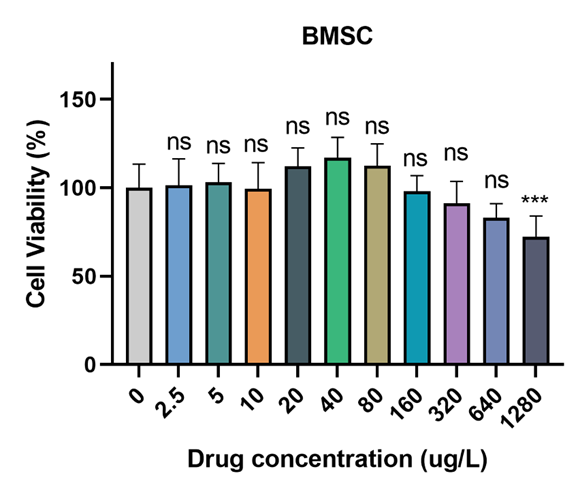
**

**Sup Fig 1.** Miacalcic had no significant effect on the proliferation activity of BMSCs. Data are presented as mean ± SD. **P < 0.01


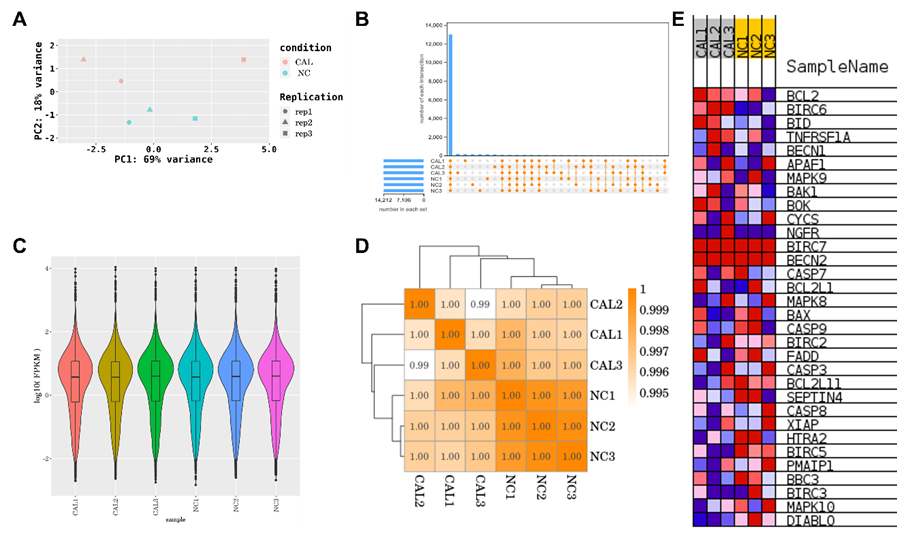


**Sup Fig 2.** Supplemental figure descriptions for sequencing. **(A-D)** Representative analysis plots reflecting the qualification of the sequencing data, as well as the heterogeneity between different subgroups and the similarity between cohorts. **(E)** Heatmap presenting some of the genes with significant differences in the pathway.


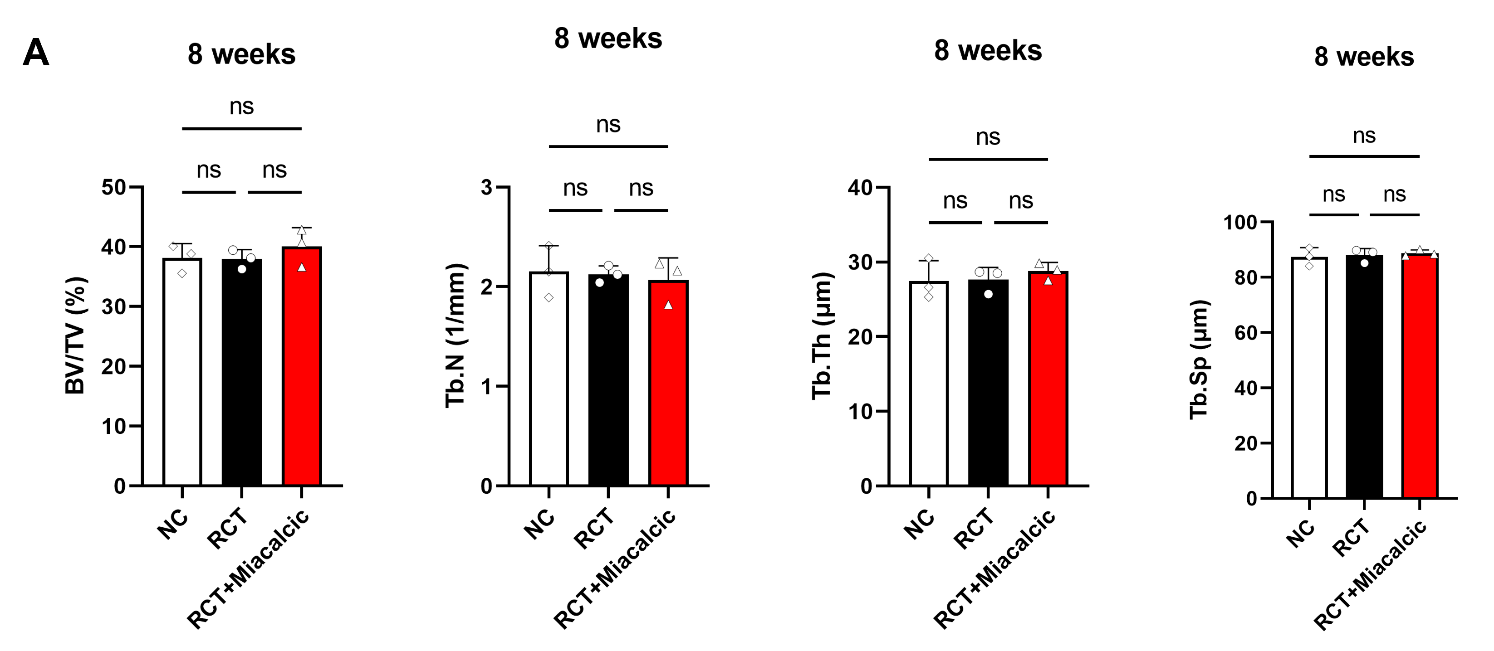


Sup Fig 3. Supplemental figure descriptions for micro-CT. (A) Different parameters of bone at 8 weeks.
