# Supplementary figures and images for: Miacalcic Enhances Rotator Cuff Injury Healing in Osteoporotic Mice by Stimulating Neovascularization via the JAK Pathway (part 2 of 2)
Source: Mediators Inflamm. 2026 Jun 3;2026:7332100. doi: 10.1155/mi/7332100 (PMC13239275; doi:10.1155/mi/7332100)

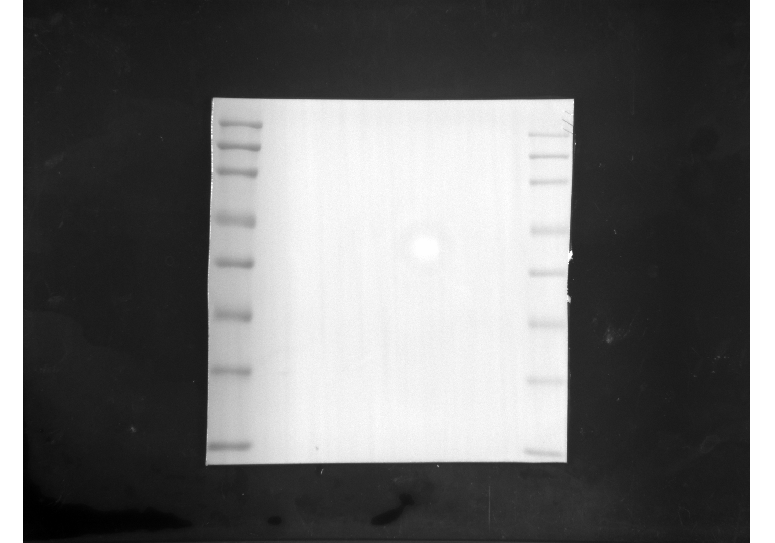

Supplement: Supplementary file 2 — Supporting Information 2 File S1: original data.zip This file contains the original Western Blot raw data images related to the study. [file MI-2026-7332100-s003.zip › 20240807/1/230706-HUVEC/mo3/1/paizhao3.tif]

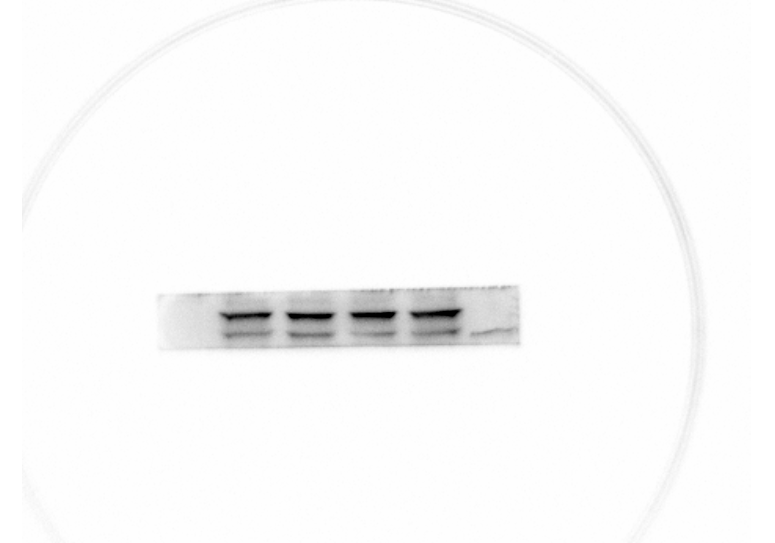

Supplement: Supplementary file 2 — Supporting Information 2 File S1: original data.zip This file contains the original Western Blot raw data images related to the study. [file MI-2026-7332100-s003.zip › 20240807/1/230706-HUVEC/mo3/1/stat3 1-1.tif]

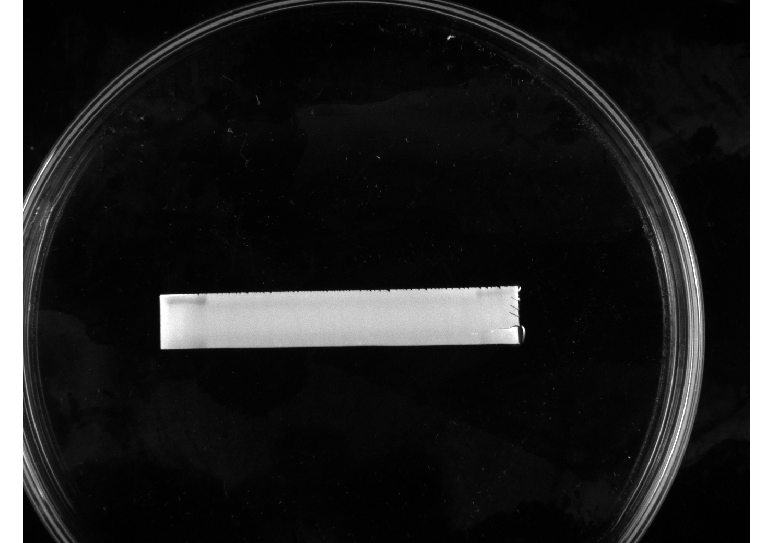

Supplement: Supplementary file 2 — Supporting Information 2 File S1: original data.zip This file contains the original Western Blot raw data images related to the study. [file MI-2026-7332100-s003.zip › 20240807/1/230706-HUVEC/mo3/1/stat3 1-3.tif]
