# Supplementary figures and images for: Miacalcic Enhances Rotator Cuff Injury Healing in Osteoporotic Mice by Stimulating Neovascularization via the JAK Pathway (part 1 of 2)
Source: Mediators Inflamm. 2026 Jun 3;2026:7332100. doi: 10.1155/mi/7332100 (PMC13239275; doi:10.1155/mi/7332100)

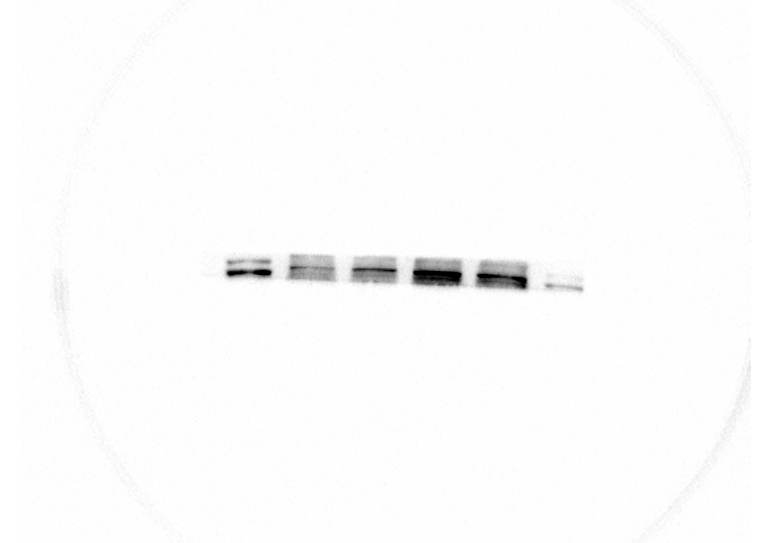

Supplement: Supplementary file 2 — Supporting Information 2 File S1: original data.zip This file contains the original Western Blot raw data images related to the study. [file MI-2026-7332100-s003.zip › 20240807/1/230630-HUVEC/mo1/1/jak1 1-1.tif]

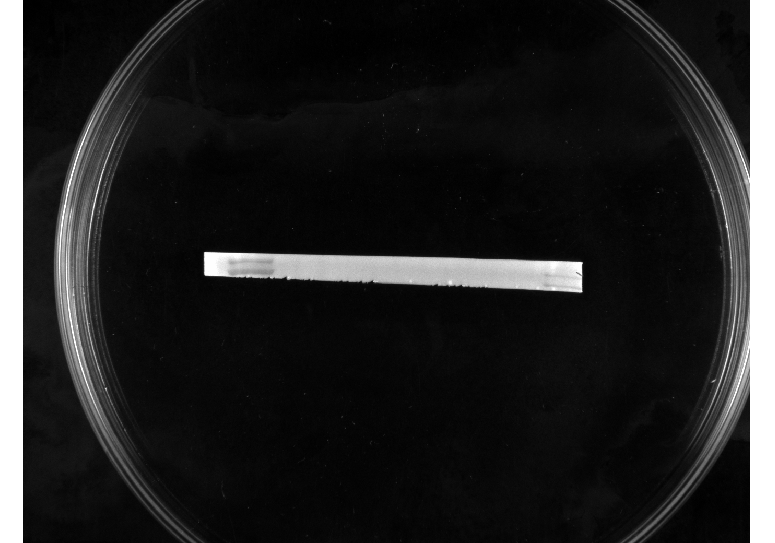

Supplement: Supplementary file 2 — Supporting Information 2 File S1: original data.zip This file contains the original Western Blot raw data images related to the study. [file MI-2026-7332100-s003.zip › 20240807/1/230630-HUVEC/mo1/1/jak1 1-3.tif]

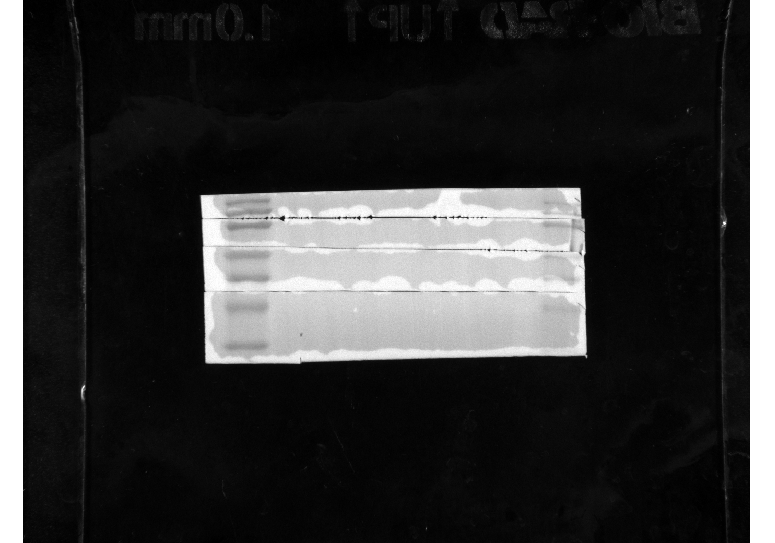

Supplement: Supplementary file 2 — Supporting Information 2 File S1: original data.zip This file contains the original Western Blot raw data images related to the study. [file MI-2026-7332100-s003.zip › 20240807/1/230630-HUVEC/mo1/1/pingmo mo1.tif]

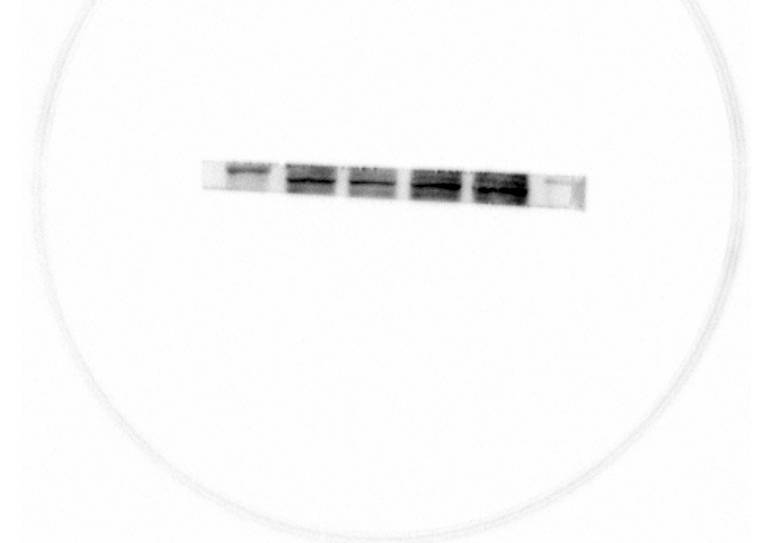

Supplement: Supplementary file 2 — Supporting Information 2 File S1: original data.zip This file contains the original Western Blot raw data images related to the study. [file MI-2026-7332100-s003.zip › 20240807/1/230630-HUVEC/mo1/1/stat3 1-1.tif]

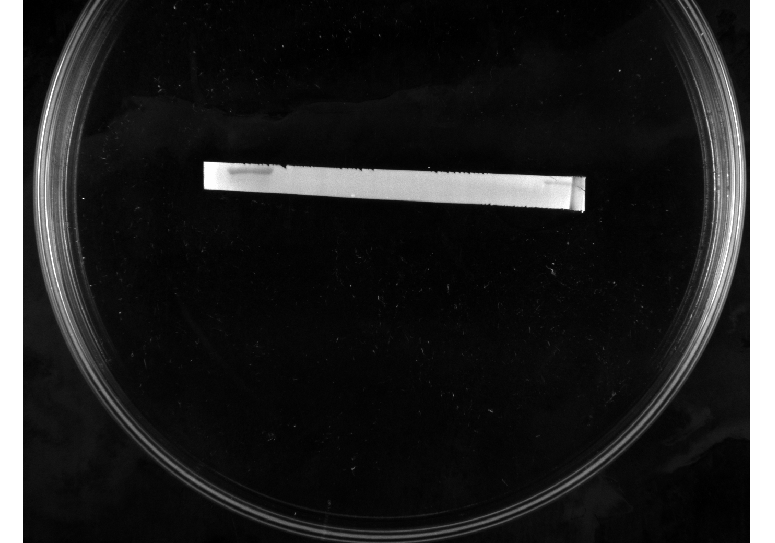

Supplement: Supplementary file 2 — Supporting Information 2 File S1: original data.zip This file contains the original Western Blot raw data images related to the study. [file MI-2026-7332100-s003.zip › 20240807/1/230630-HUVEC/mo1/1/stat3 1-3.tif]

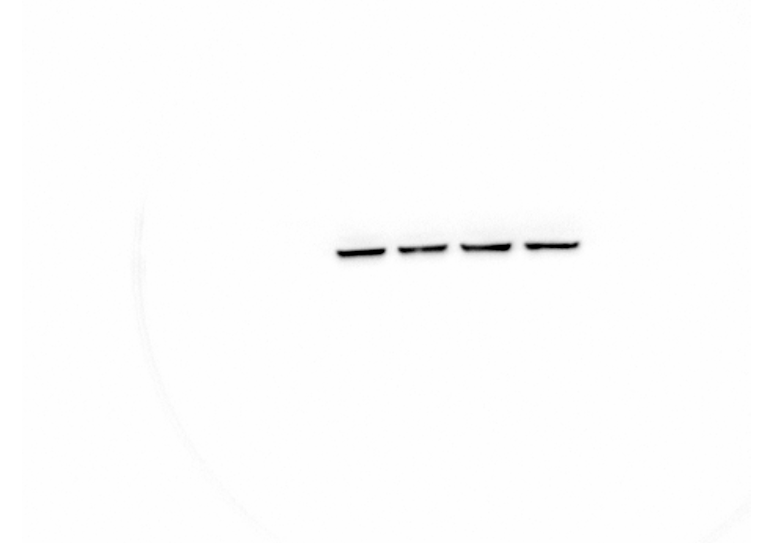

Supplement: Supplementary file 2 — Supporting Information 2 File S1: original data.zip This file contains the original Western Blot raw data images related to the study. [file MI-2026-7332100-s003.zip › 20240807/1/230630-HUVEC/mo1/1/tublin 1-1.tif]

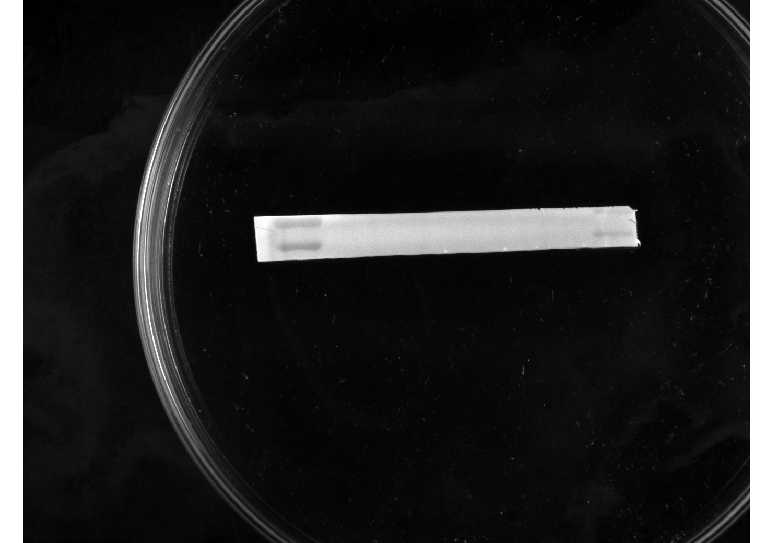

Supplement: Supplementary file 2 — Supporting Information 2 File S1: original data.zip This file contains the original Western Blot raw data images related to the study. [file MI-2026-7332100-s003.zip › 20240807/1/230630-HUVEC/mo1/1/tublin 1-3.tif]

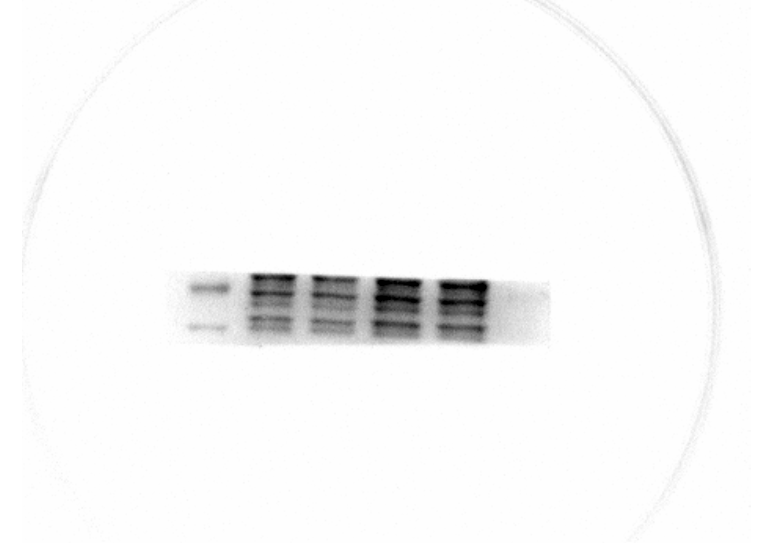

Supplement: Supplementary file 2 — Supporting Information 2 File S1: original data.zip This file contains the original Western Blot raw data images related to the study. [file MI-2026-7332100-s003.zip › 20240807/1/230630-HUVEC/mo1/1/vegf 1-1.tif]

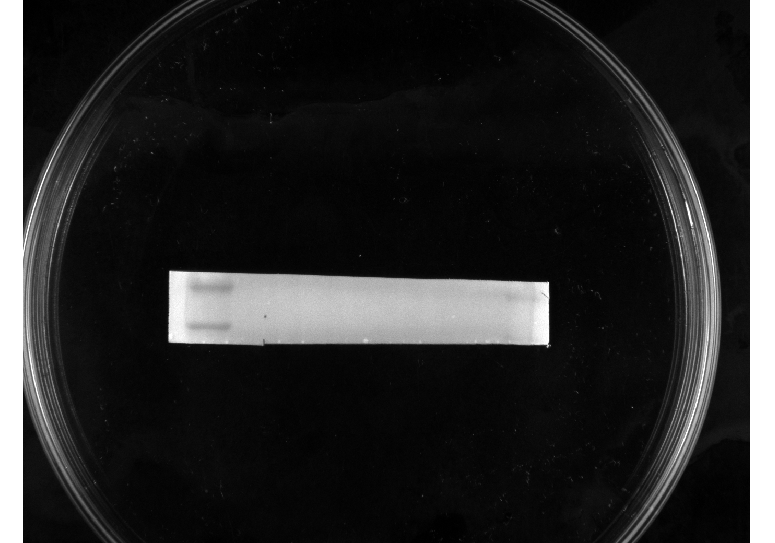

Supplement: Supplementary file 2 — Supporting Information 2 File S1: original data.zip This file contains the original Western Blot raw data images related to the study. [file MI-2026-7332100-s003.zip › 20240807/1/230630-HUVEC/mo1/1/vegf 1-3.tif]

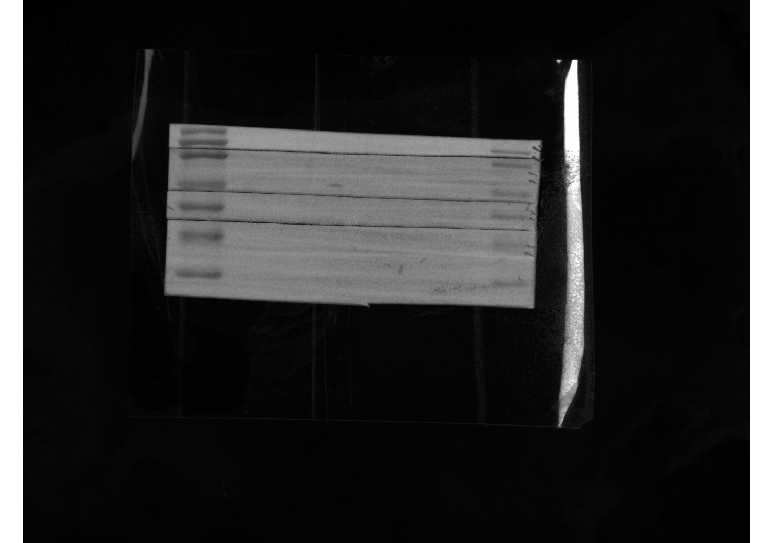

Supplement: Supplementary file 2 — Supporting Information 2 File S1: original data.zip This file contains the original Western Blot raw data images related to the study. [file MI-2026-7332100-s003.zip › 20240807/1/230630-HUVEC/mo2/1/H-n2.tif]

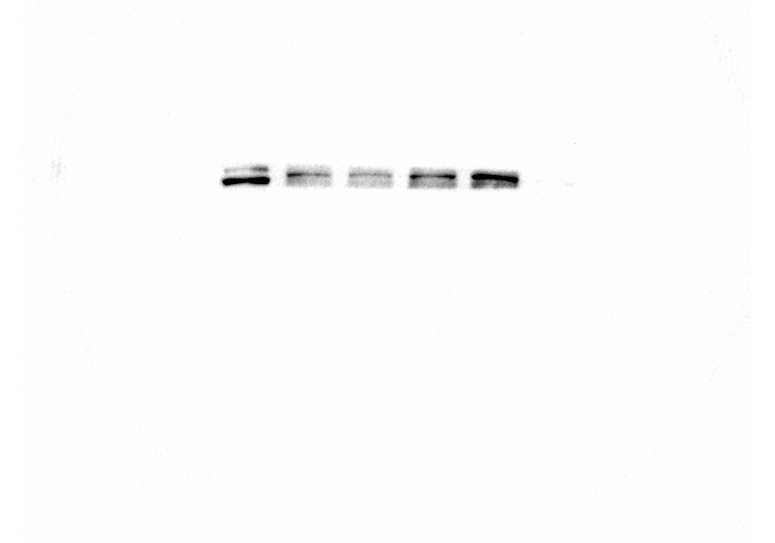

Supplement: Supplementary file 2 — Supporting Information 2 File S1: original data.zip This file contains the original Western Blot raw data images related to the study. [file MI-2026-7332100-s003.zip › 20240807/1/230630-HUVEC/mo2/1/jak1 1-1.tif]

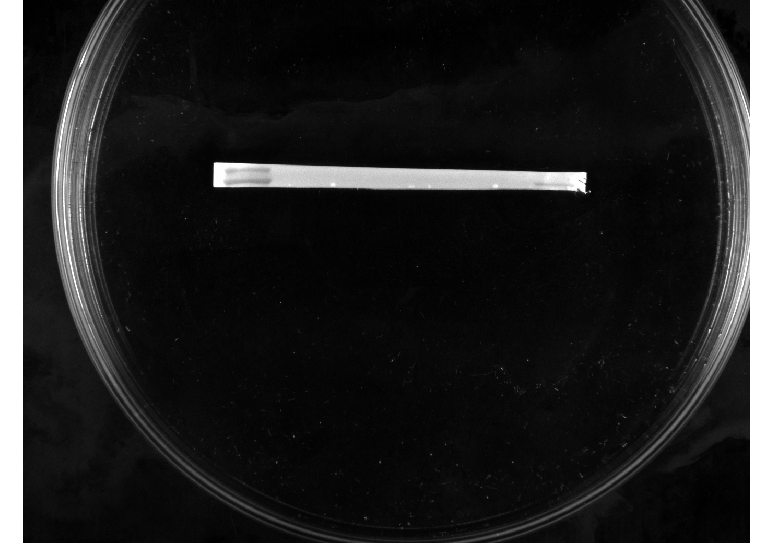

Supplement: Supplementary file 2 — Supporting Information 2 File S1: original data.zip This file contains the original Western Blot raw data images related to the study. [file MI-2026-7332100-s003.zip › 20240807/1/230630-HUVEC/mo2/1/jak1 1-3.tif]

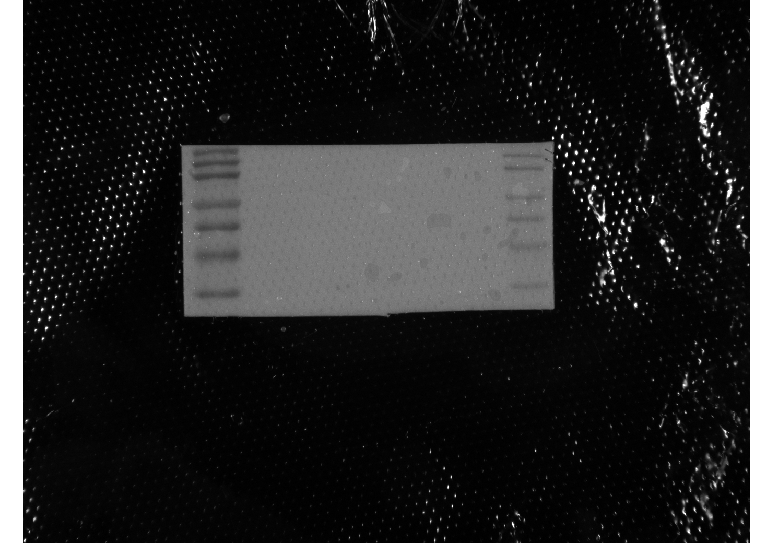

Supplement: Supplementary file 2 — Supporting Information 2 File S1: original data.zip This file contains the original Western Blot raw data images related to the study. [file MI-2026-7332100-s003.zip › 20240807/1/230630-HUVEC/mo2/1/mo2.tif]

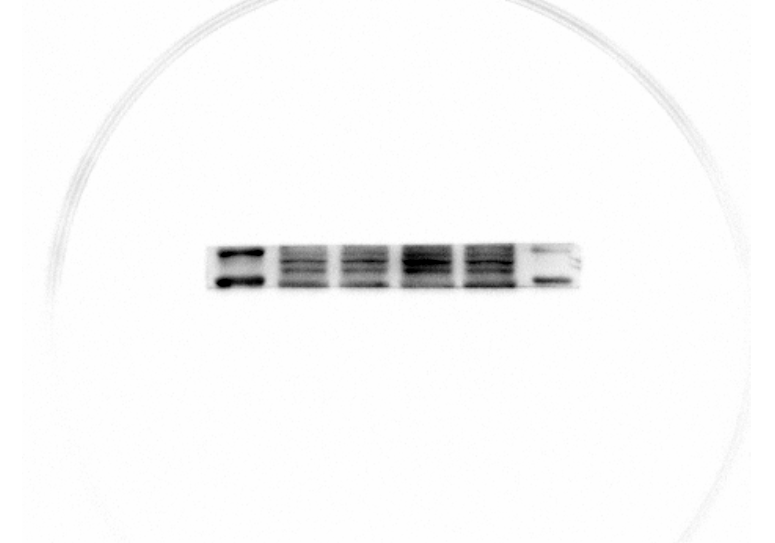

Supplement: Supplementary file 2 — Supporting Information 2 File S1: original data.zip This file contains the original Western Blot raw data images related to the study. [file MI-2026-7332100-s003.zip › 20240807/1/230630-HUVEC/mo2/1/stat3 1-1.tif]

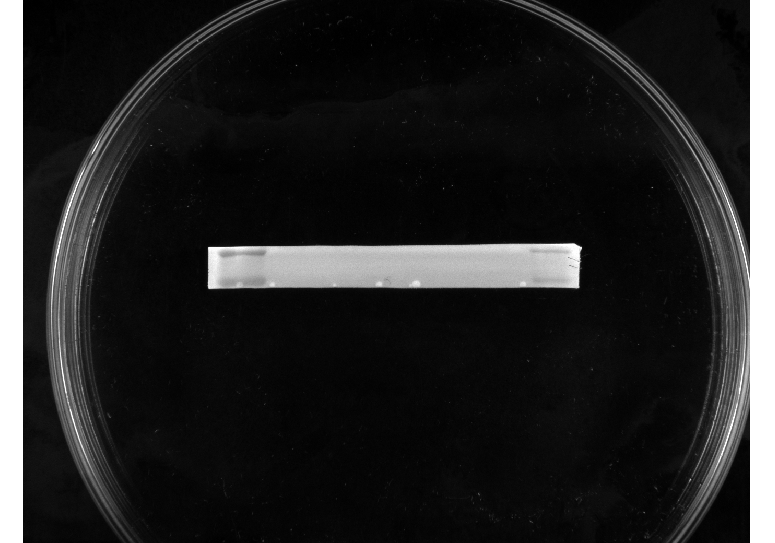

Supplement: Supplementary file 2 — Supporting Information 2 File S1: original data.zip This file contains the original Western Blot raw data images related to the study. [file MI-2026-7332100-s003.zip › 20240807/1/230630-HUVEC/mo2/1/stat3 1-3.tif]

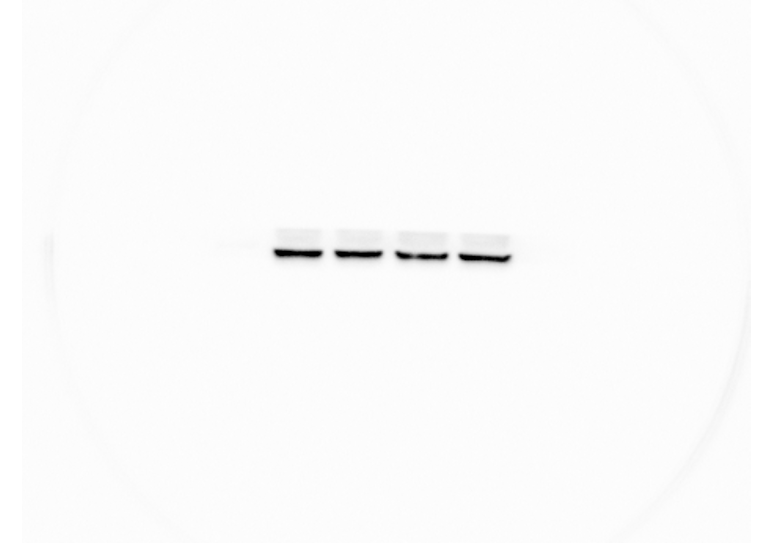

Supplement: Supplementary file 2 — Supporting Information 2 File S1: original data.zip This file contains the original Western Blot raw data images related to the study. [file MI-2026-7332100-s003.zip › 20240807/1/230630-HUVEC/mo2/1/tublin 1-1.tif]

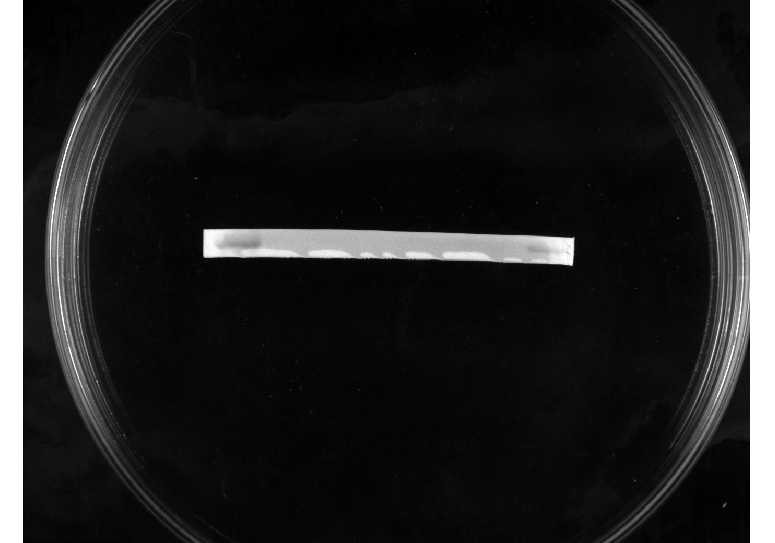

Supplement: Supplementary file 2 — Supporting Information 2 File S1: original data.zip This file contains the original Western Blot raw data images related to the study. [file MI-2026-7332100-s003.zip › 20240807/1/230630-HUVEC/mo2/1/tublin 1-3.tif]

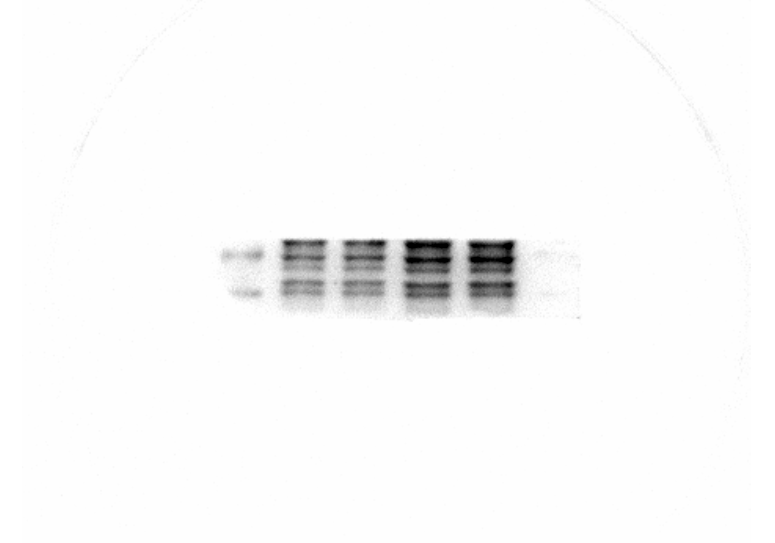

Supplement: Supplementary file 2 — Supporting Information 2 File S1: original data.zip This file contains the original Western Blot raw data images related to the study. [file MI-2026-7332100-s003.zip › 20240807/1/230630-HUVEC/mo2/1/vegf 1-1.tif]

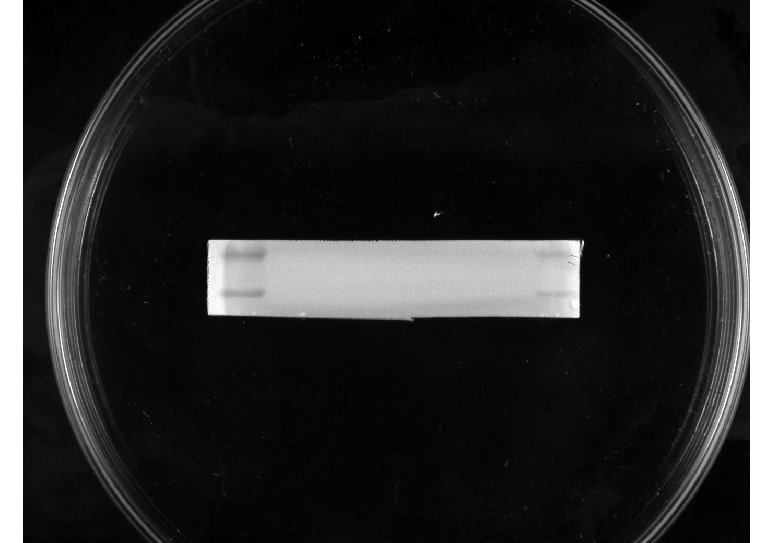

Supplement: Supplementary file 2 — Supporting Information 2 File S1: original data.zip This file contains the original Western Blot raw data images related to the study. [file MI-2026-7332100-s003.zip › 20240807/1/230630-HUVEC/mo2/1/vegf 1-3.tif]

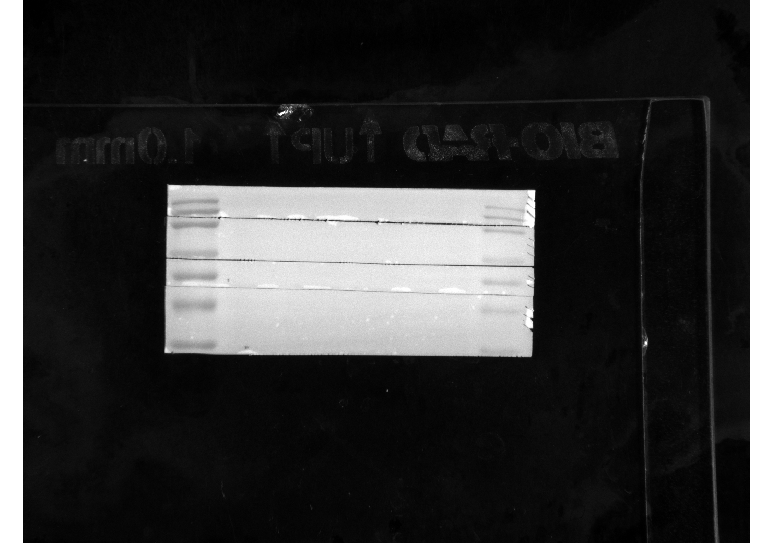

Supplement: Supplementary file 2 — Supporting Information 2 File S1: original data.zip This file contains the original Western Blot raw data images related to the study. [file MI-2026-7332100-s003.zip › 20240807/1/230630-HUVEC/mo3/1/H-n3.tif]

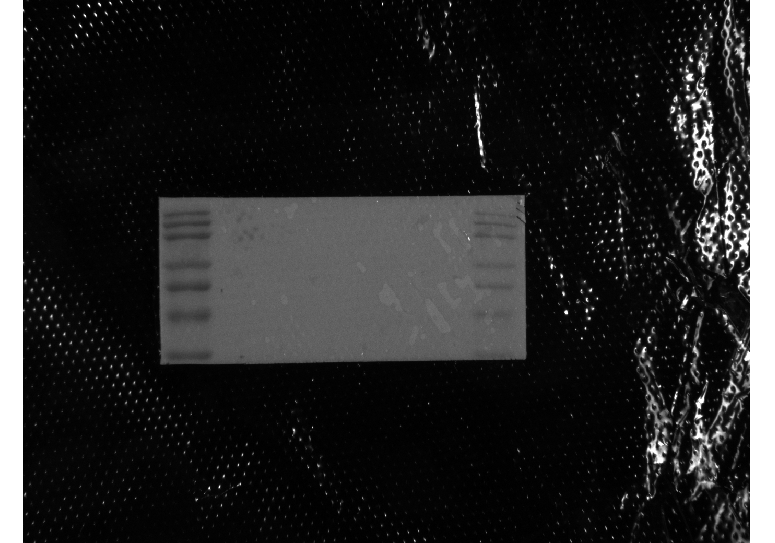

Supplement: Supplementary file 2 — Supporting Information 2 File S1: original data.zip This file contains the original Western Blot raw data images related to the study. [file MI-2026-7332100-s003.zip › 20240807/1/230630-HUVEC/mo3/1/mo3.tif]

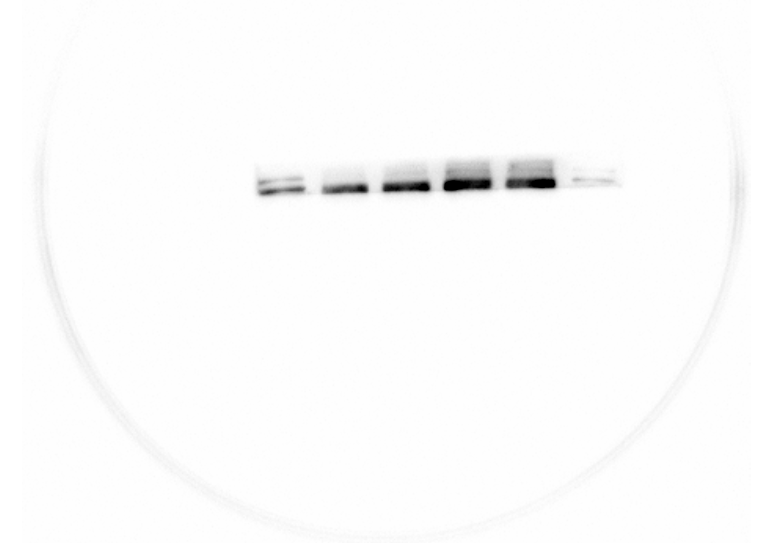

Supplement: Supplementary file 2 — Supporting Information 2 File S1: original data.zip This file contains the original Western Blot raw data images related to the study. [file MI-2026-7332100-s003.zip › 20240807/1/230630-HUVEC/mo3/1/p-jak1 1-1.tif]

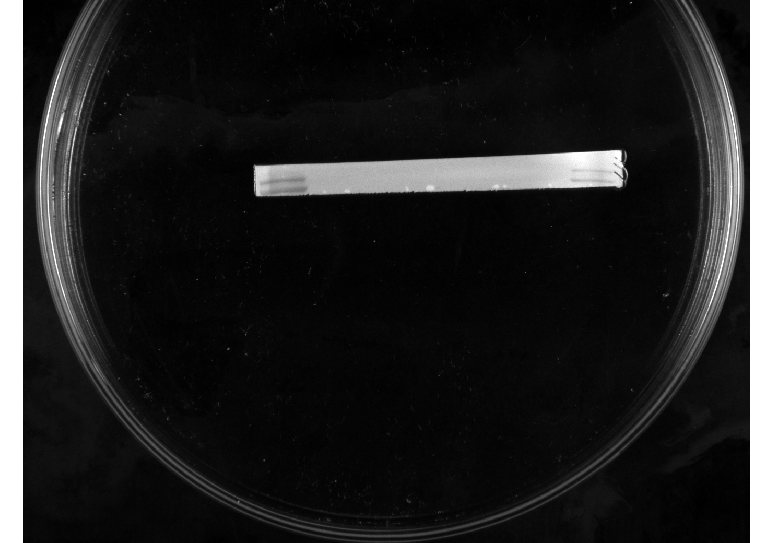

Supplement: Supplementary file 2 — Supporting Information 2 File S1: original data.zip This file contains the original Western Blot raw data images related to the study. [file MI-2026-7332100-s003.zip › 20240807/1/230630-HUVEC/mo3/1/p-jak1 1-3.tif]

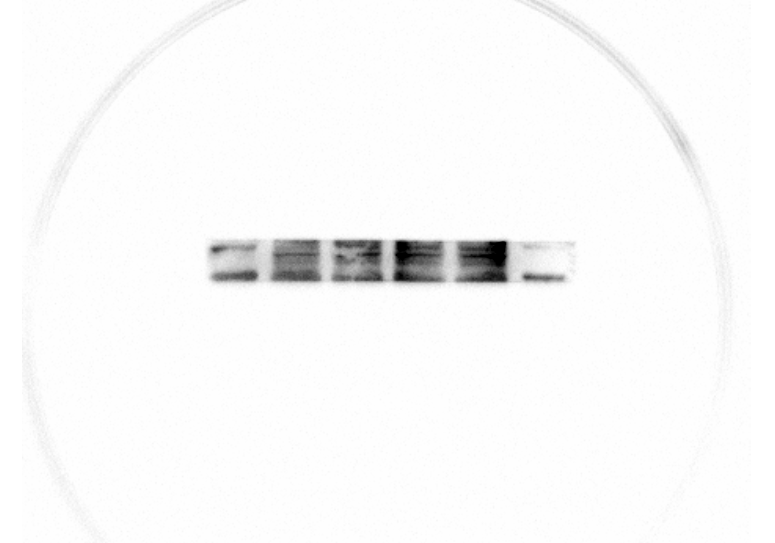

Supplement: Supplementary file 2 — Supporting Information 2 File S1: original data.zip This file contains the original Western Blot raw data images related to the study. [file MI-2026-7332100-s003.zip › 20240807/1/230630-HUVEC/mo3/1/p-stat3 1-1.tif]

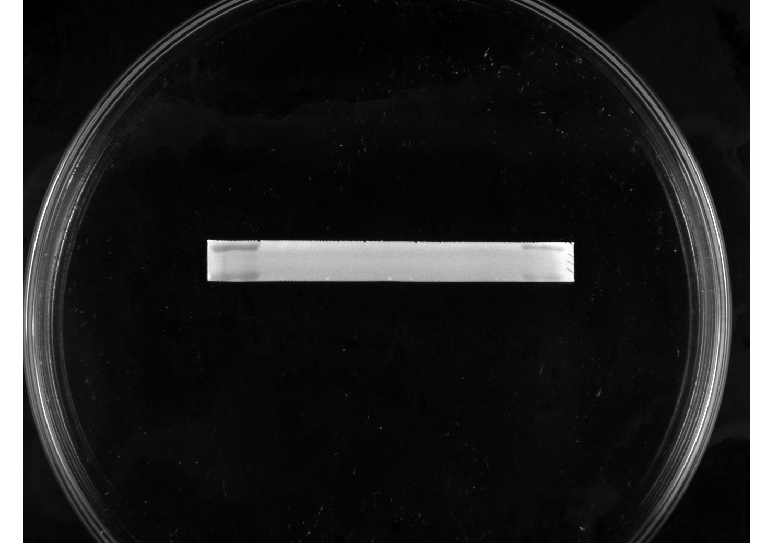

Supplement: Supplementary file 2 — Supporting Information 2 File S1: original data.zip This file contains the original Western Blot raw data images related to the study. [file MI-2026-7332100-s003.zip › 20240807/1/230630-HUVEC/mo3/1/p-stat3 1-3.tif]

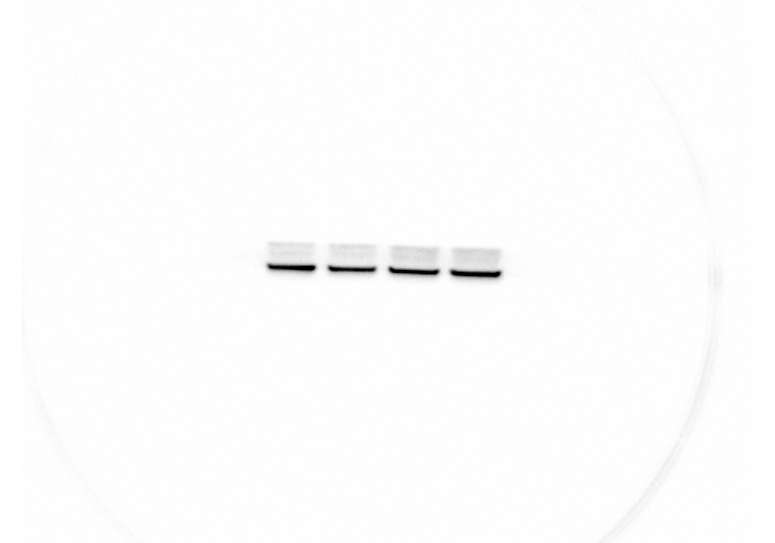

Supplement: Supplementary file 2 — Supporting Information 2 File S1: original data.zip This file contains the original Western Blot raw data images related to the study. [file MI-2026-7332100-s003.zip › 20240807/1/230630-HUVEC/mo3/1/tublin 1-1.tif]

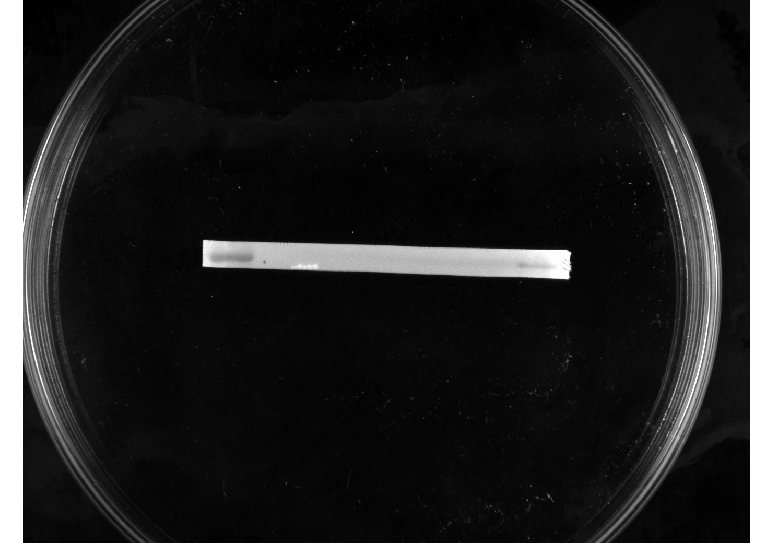

Supplement: Supplementary file 2 — Supporting Information 2 File S1: original data.zip This file contains the original Western Blot raw data images related to the study. [file MI-2026-7332100-s003.zip › 20240807/1/230630-HUVEC/mo3/1/tublin 1-3.tif]

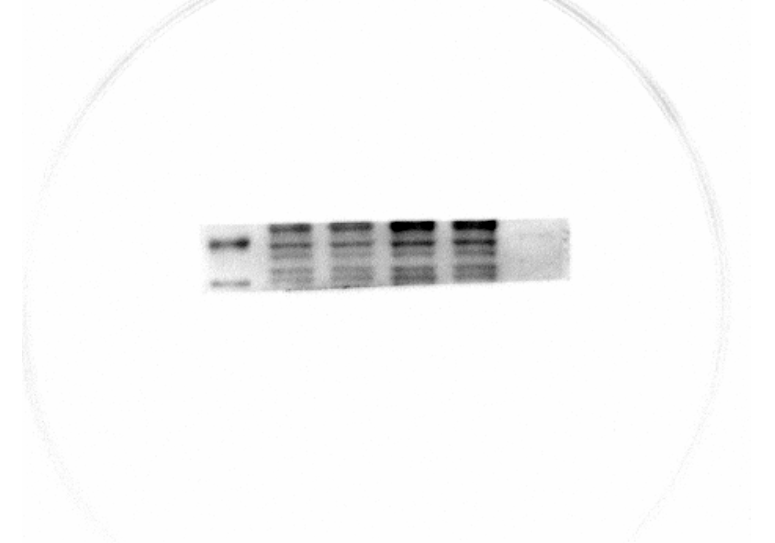

Supplement: Supplementary file 2 — Supporting Information 2 File S1: original data.zip This file contains the original Western Blot raw data images related to the study. [file MI-2026-7332100-s003.zip › 20240807/1/230630-HUVEC/mo3/1/vegf 1-1.tif]

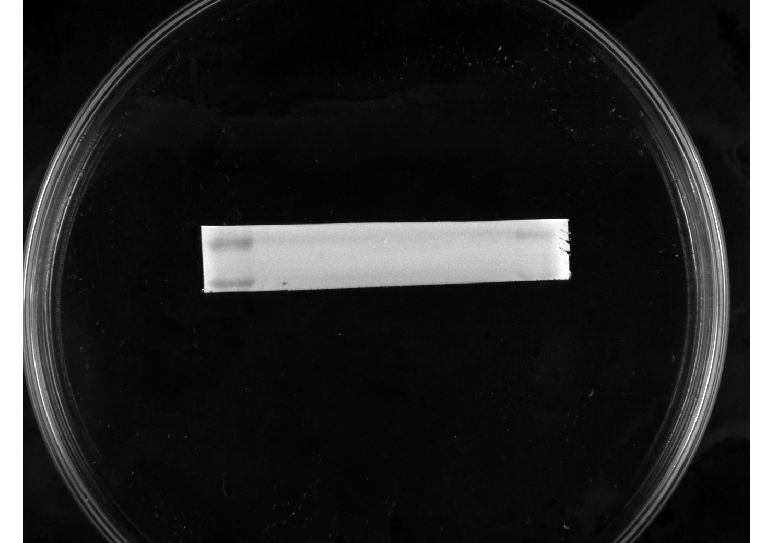

Supplement: Supplementary file 2 — Supporting Information 2 File S1: original data.zip This file contains the original Western Blot raw data images related to the study. [file MI-2026-7332100-s003.zip › 20240807/1/230630-HUVEC/mo3/1/vegf 1-3.tif]

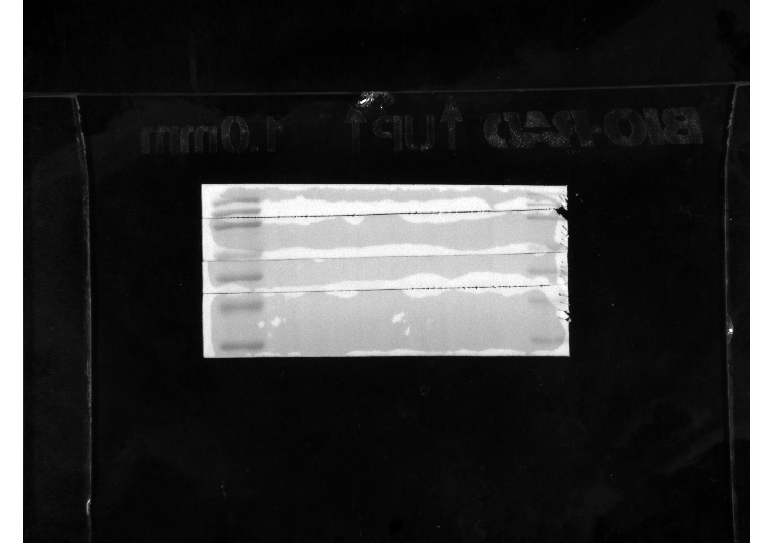

Supplement: Supplementary file 2 — Supporting Information 2 File S1: original data.zip This file contains the original Western Blot raw data images related to the study. [file MI-2026-7332100-s003.zip › 20240807/1/230630-HUVEC/mo4/1/H-n4.tif]

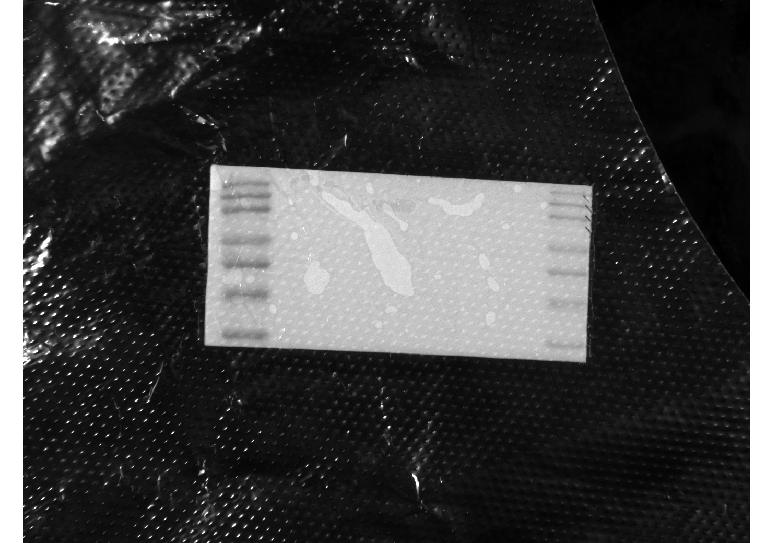

Supplement: Supplementary file 2 — Supporting Information 2 File S1: original data.zip This file contains the original Western Blot raw data images related to the study. [file MI-2026-7332100-s003.zip › 20240807/1/230630-HUVEC/mo4/1/mo4.tif]

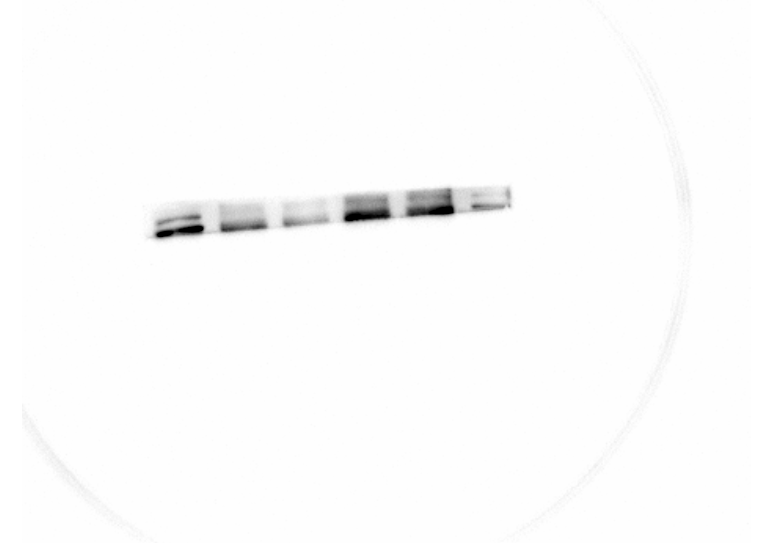

Supplement: Supplementary file 2 — Supporting Information 2 File S1: original data.zip This file contains the original Western Blot raw data images related to the study. [file MI-2026-7332100-s003.zip › 20240807/1/230630-HUVEC/mo4/1/p-jak1 1-1.tif]

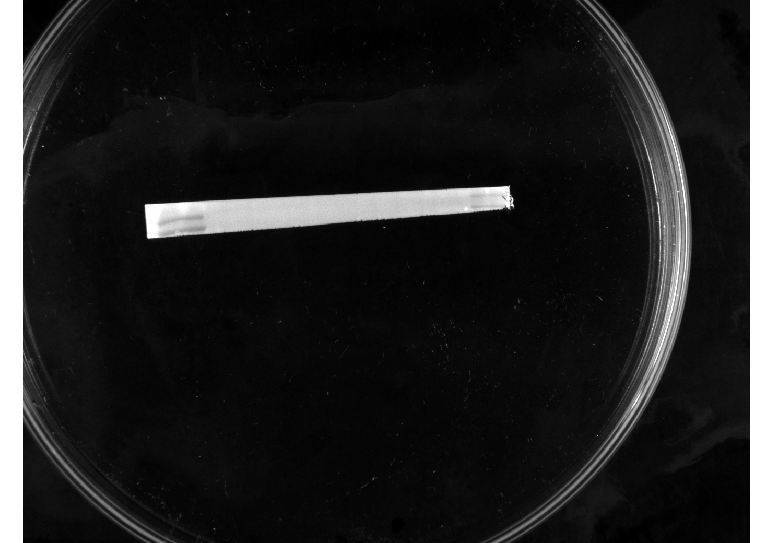

Supplement: Supplementary file 2 — Supporting Information 2 File S1: original data.zip This file contains the original Western Blot raw data images related to the study. [file MI-2026-7332100-s003.zip › 20240807/1/230630-HUVEC/mo4/1/p-jak1 1-3.tif]

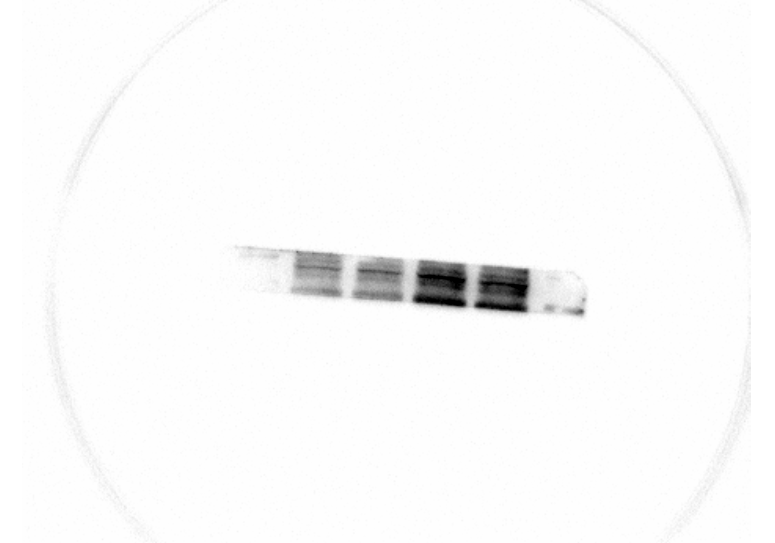

Supplement: Supplementary file 2 — Supporting Information 2 File S1: original data.zip This file contains the original Western Blot raw data images related to the study. [file MI-2026-7332100-s003.zip › 20240807/1/230630-HUVEC/mo4/1/p-stat3 1-1.tif]

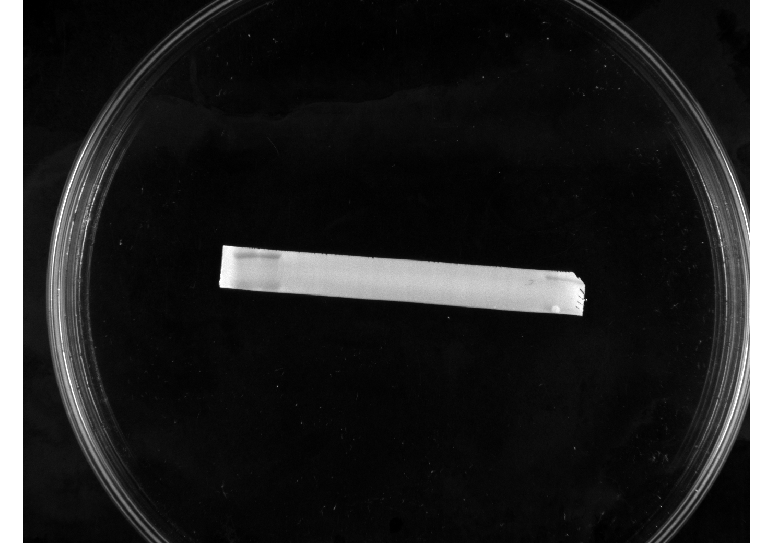

Supplement: Supplementary file 2 — Supporting Information 2 File S1: original data.zip This file contains the original Western Blot raw data images related to the study. [file MI-2026-7332100-s003.zip › 20240807/1/230630-HUVEC/mo4/1/p-stat3 1-3.tif]

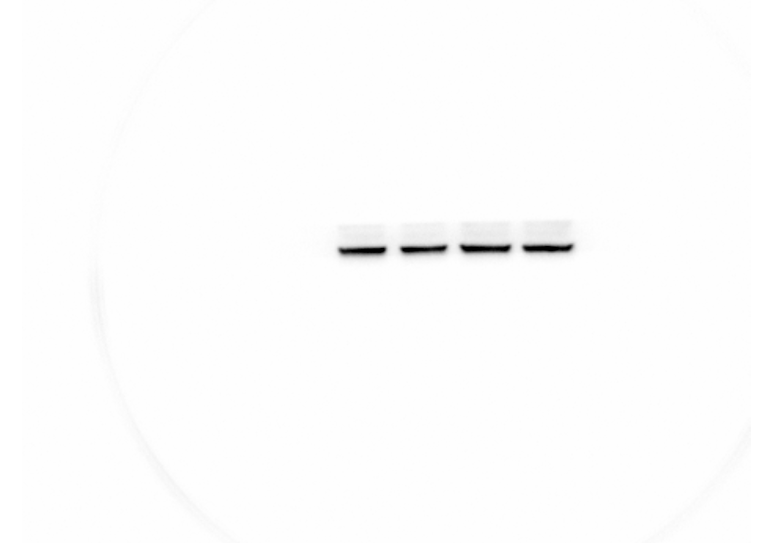

Supplement: Supplementary file 2 — Supporting Information 2 File S1: original data.zip This file contains the original Western Blot raw data images related to the study. [file MI-2026-7332100-s003.zip › 20240807/1/230630-HUVEC/mo4/1/tublin 1-1.tif]

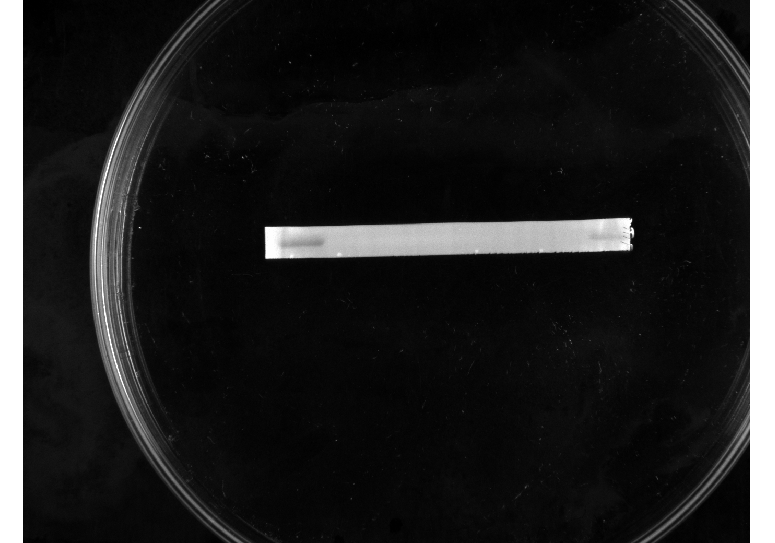

Supplement: Supplementary file 2 — Supporting Information 2 File S1: original data.zip This file contains the original Western Blot raw data images related to the study. [file MI-2026-7332100-s003.zip › 20240807/1/230630-HUVEC/mo4/1/tublin 1-3.tif]

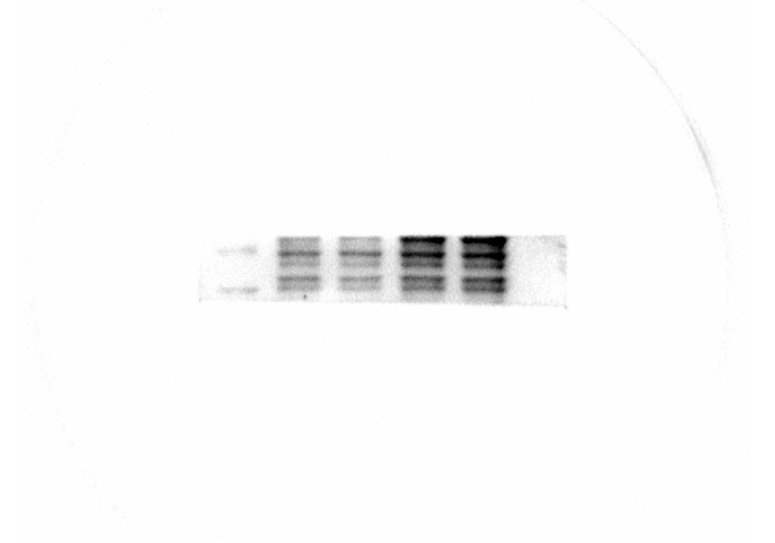

Supplement: Supplementary file 2 — Supporting Information 2 File S1: original data.zip This file contains the original Western Blot raw data images related to the study. [file MI-2026-7332100-s003.zip › 20240807/1/230630-HUVEC/mo4/1/vegf 1-1.tif]

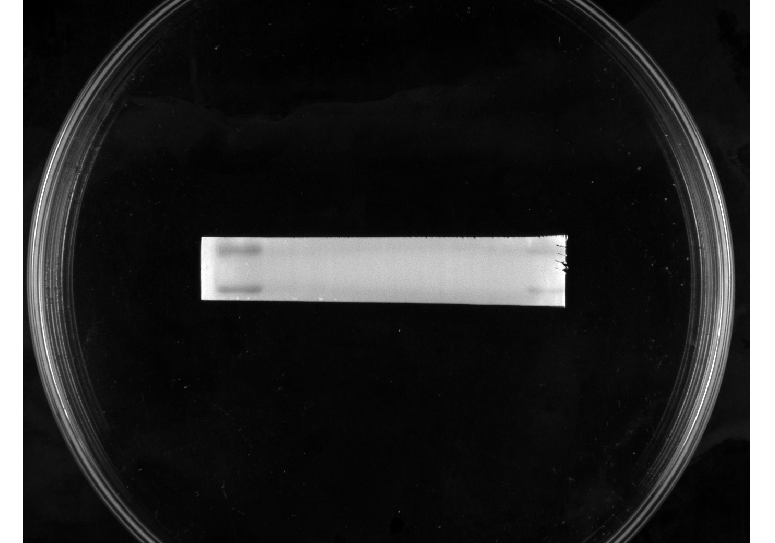

Supplement: Supplementary file 2 — Supporting Information 2 File S1: original data.zip This file contains the original Western Blot raw data images related to the study. [file MI-2026-7332100-s003.zip › 20240807/1/230630-HUVEC/mo4/1/vegf 1-3.tif]

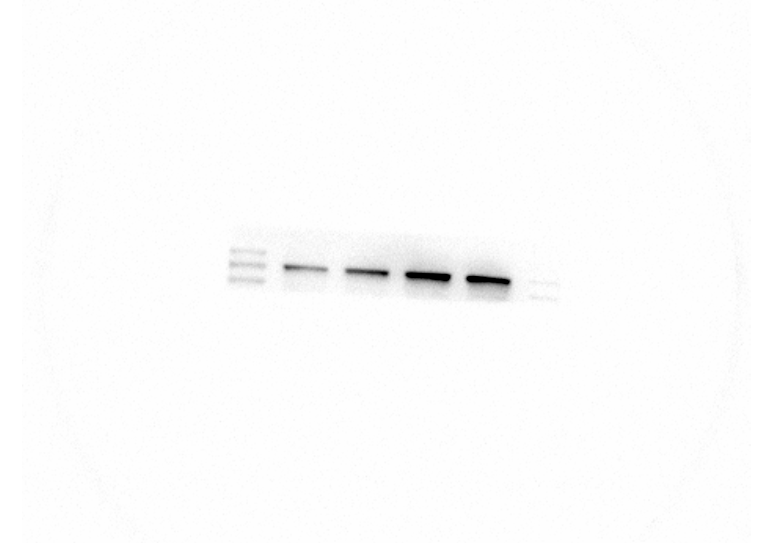

Supplement: Supplementary file 2 — Supporting Information 2 File S1: original data.zip This file contains the original Western Blot raw data images related to the study. [file MI-2026-7332100-s003.zip › 20240807/1/230703-HUVEC/mo1/1/cd31 2-1.tif]

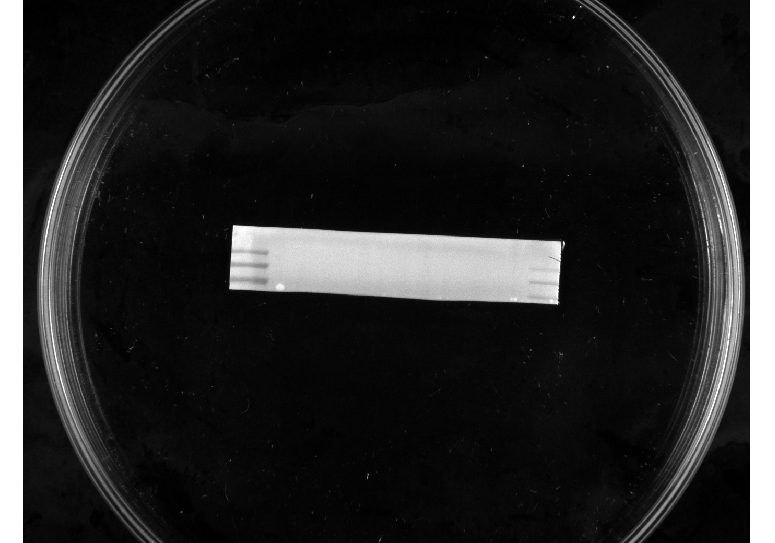

Supplement: Supplementary file 2 — Supporting Information 2 File S1: original data.zip This file contains the original Western Blot raw data images related to the study. [file MI-2026-7332100-s003.zip › 20240807/1/230703-HUVEC/mo1/1/cd31 2-3.tif]

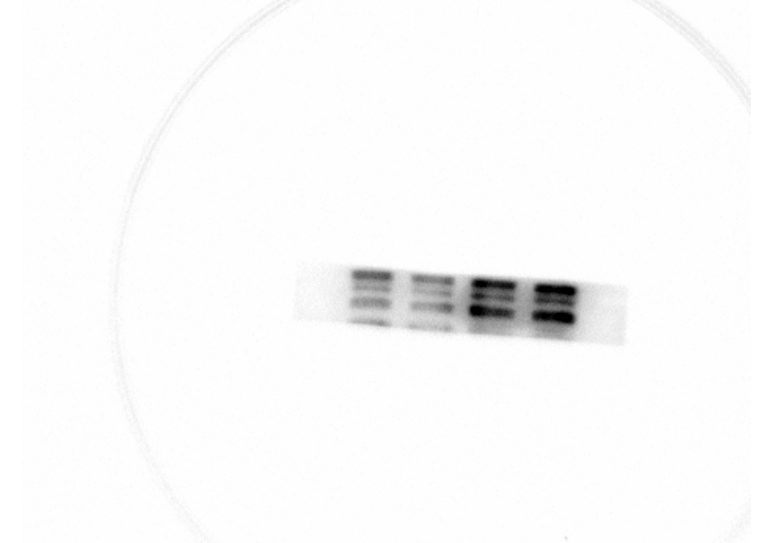

Supplement: Supplementary file 2 — Supporting Information 2 File S1: original data.zip This file contains the original Western Blot raw data images related to the study. [file MI-2026-7332100-s003.zip › 20240807/1/230703-HUVEC/mo1/1/cylin d1 1-1.tif]

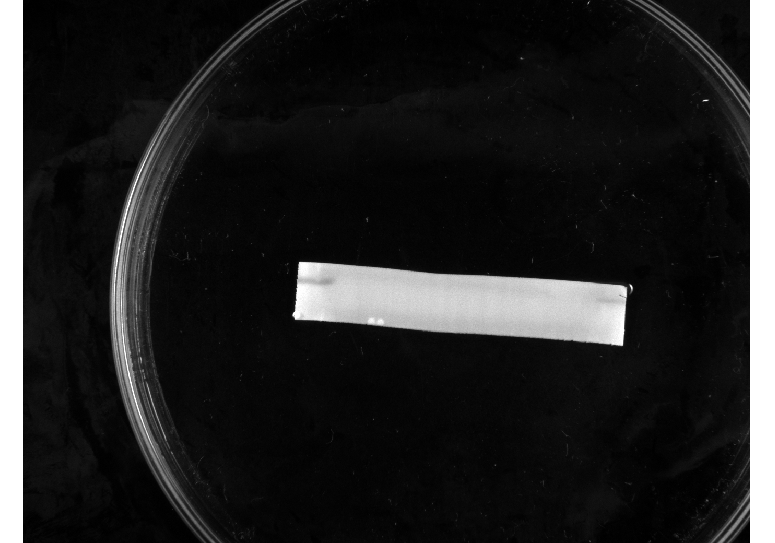

Supplement: Supplementary file 2 — Supporting Information 2 File S1: original data.zip This file contains the original Western Blot raw data images related to the study. [file MI-2026-7332100-s003.zip › 20240807/1/230703-HUVEC/mo1/1/cylin d1 1-3.tif]

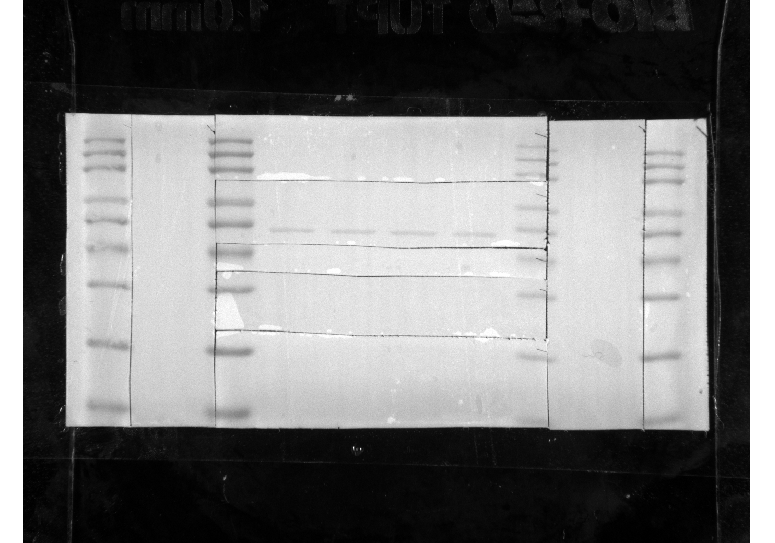

Supplement: Supplementary file 2 — Supporting Information 2 File S1: original data.zip This file contains the original Western Blot raw data images related to the study. [file MI-2026-7332100-s003.zip › 20240807/1/230703-HUVEC/mo1/1/H-n1.tif]

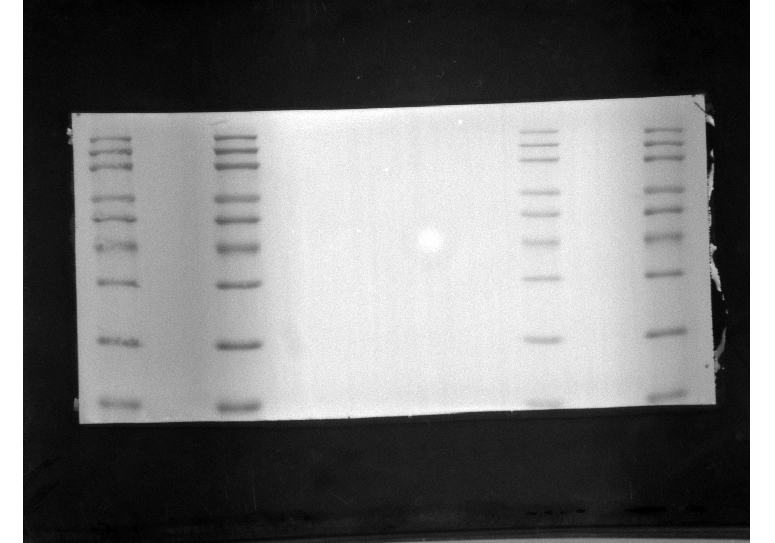

Supplement: Supplementary file 2 — Supporting Information 2 File S1: original data.zip This file contains the original Western Blot raw data images related to the study. [file MI-2026-7332100-s003.zip › 20240807/1/230703-HUVEC/mo1/1/paimo1.tif]

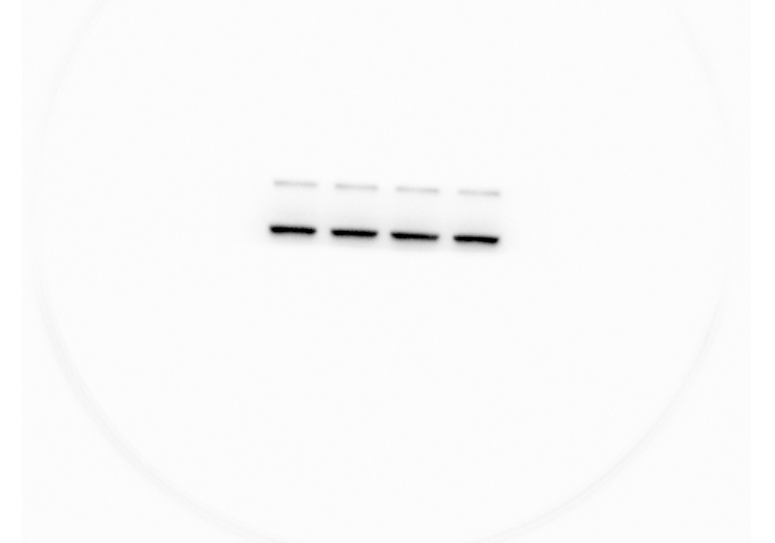

Supplement: Supplementary file 2 — Supporting Information 2 File S1: original data.zip This file contains the original Western Blot raw data images related to the study. [file MI-2026-7332100-s003.zip › 20240807/1/230703-HUVEC/mo1/1/tublin 1-1.tif]

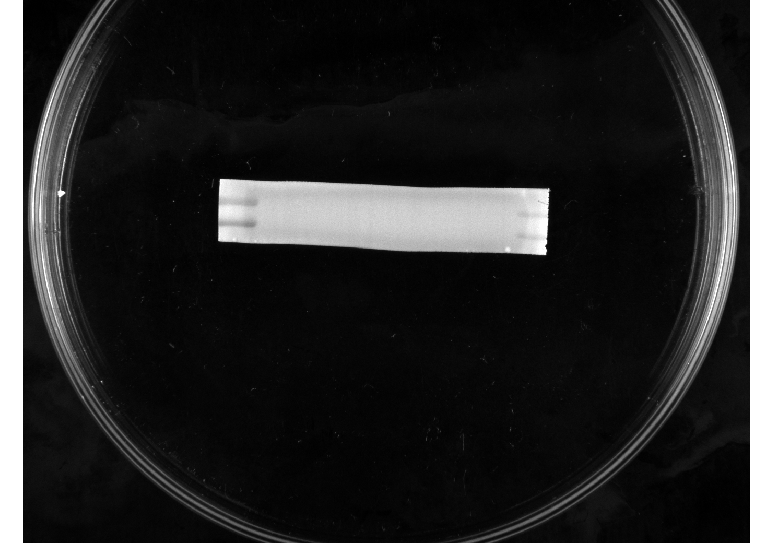

Supplement: Supplementary file 2 — Supporting Information 2 File S1: original data.zip This file contains the original Western Blot raw data images related to the study. [file MI-2026-7332100-s003.zip › 20240807/1/230703-HUVEC/mo1/1/tublin 1-3.tif]

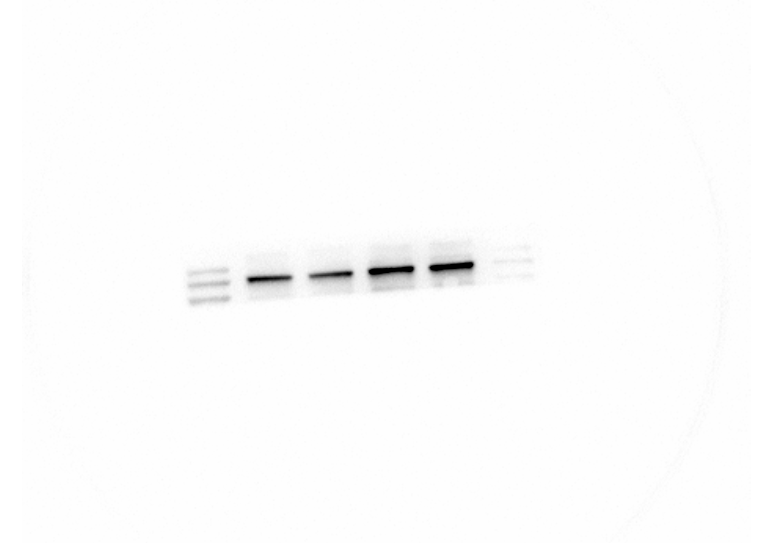

Supplement: Supplementary file 2 — Supporting Information 2 File S1: original data.zip This file contains the original Western Blot raw data images related to the study. [file MI-2026-7332100-s003.zip › 20240807/1/230703-HUVEC/mo2/1/cd31 1-1.tif]

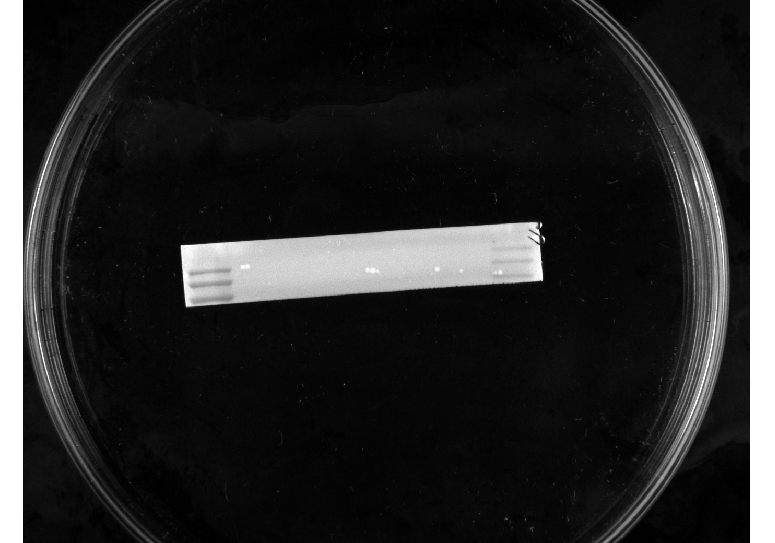

Supplement: Supplementary file 2 — Supporting Information 2 File S1: original data.zip This file contains the original Western Blot raw data images related to the study. [file MI-2026-7332100-s003.zip › 20240807/1/230703-HUVEC/mo2/1/cd31 1-3.tif]

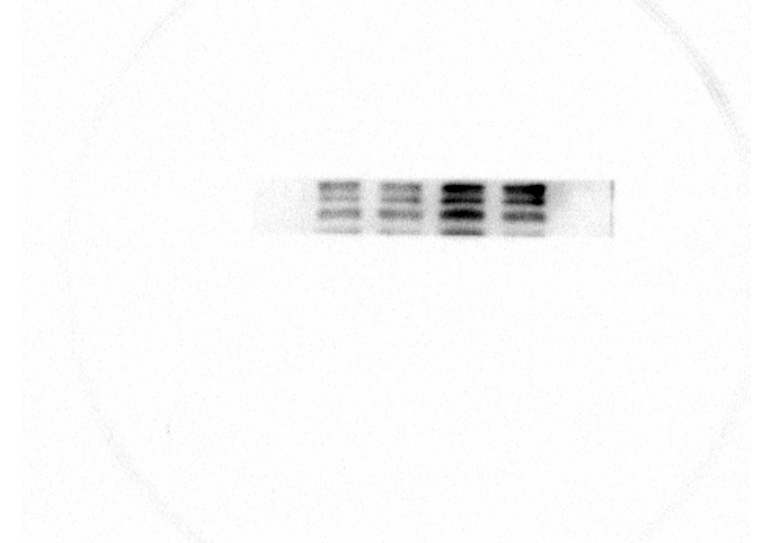

Supplement: Supplementary file 2 — Supporting Information 2 File S1: original data.zip This file contains the original Western Blot raw data images related to the study. [file MI-2026-7332100-s003.zip › 20240807/1/230703-HUVEC/mo2/1/cylin d1 1-1.tif]

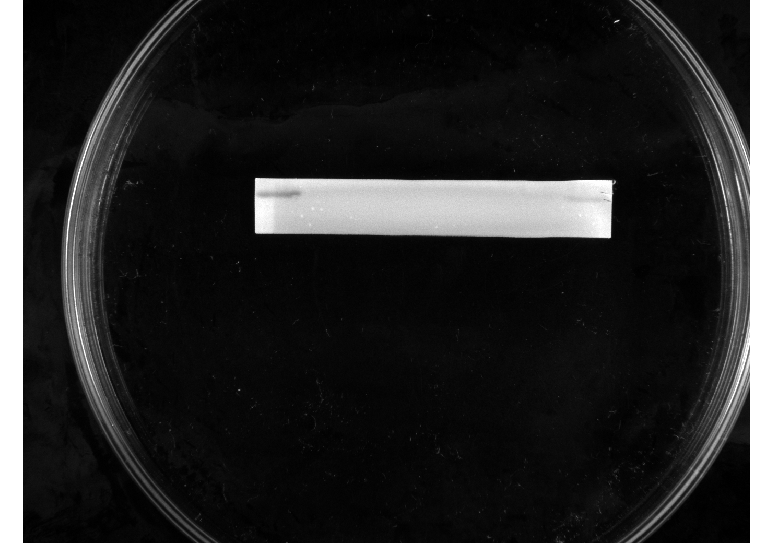

Supplement: Supplementary file 2 — Supporting Information 2 File S1: original data.zip This file contains the original Western Blot raw data images related to the study. [file MI-2026-7332100-s003.zip › 20240807/1/230703-HUVEC/mo2/1/cylin d1 1-3.tif]

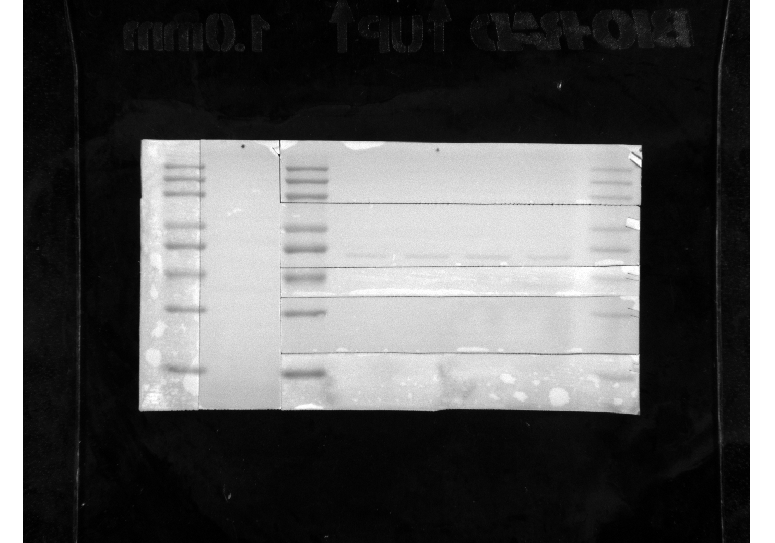

Supplement: Supplementary file 2 — Supporting Information 2 File S1: original data.zip This file contains the original Western Blot raw data images related to the study. [file MI-2026-7332100-s003.zip › 20240807/1/230703-HUVEC/mo2/1/H-n2.tif]

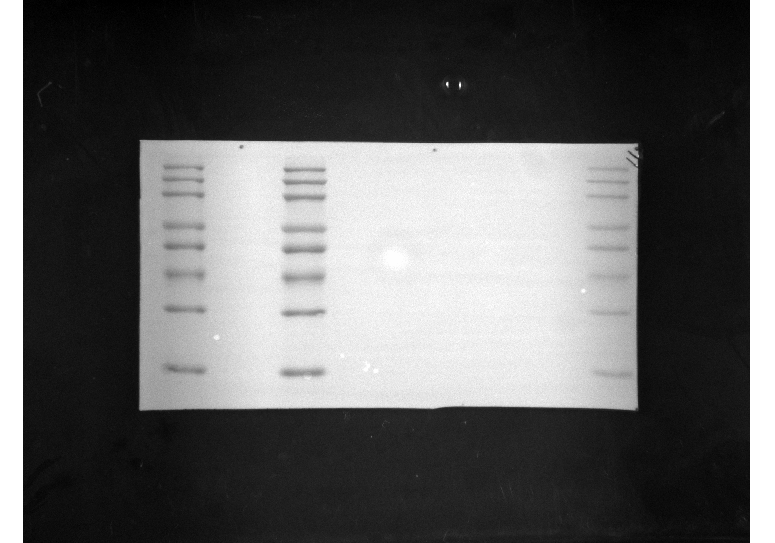

Supplement: Supplementary file 2 — Supporting Information 2 File S1: original data.zip This file contains the original Western Blot raw data images related to the study. [file MI-2026-7332100-s003.zip › 20240807/1/230703-HUVEC/mo2/1/paimo2.tif]

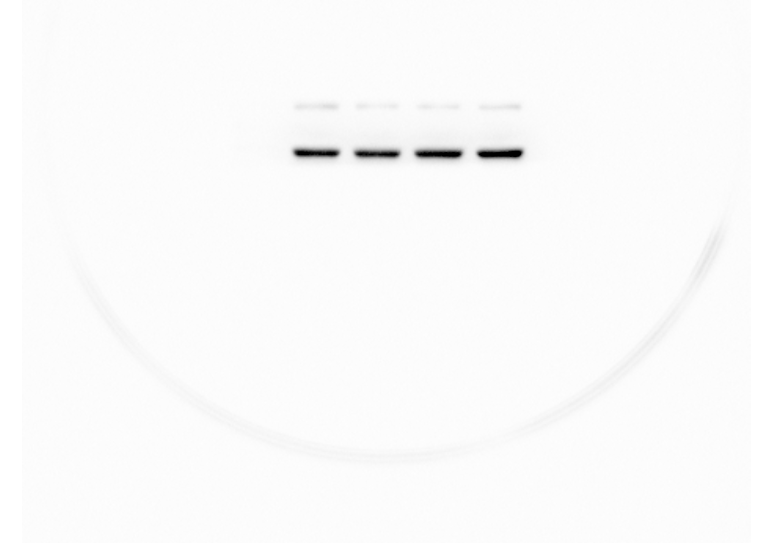

Supplement: Supplementary file 2 — Supporting Information 2 File S1: original data.zip This file contains the original Western Blot raw data images related to the study. [file MI-2026-7332100-s003.zip › 20240807/1/230703-HUVEC/mo2/1/tublin 1-1.tif]

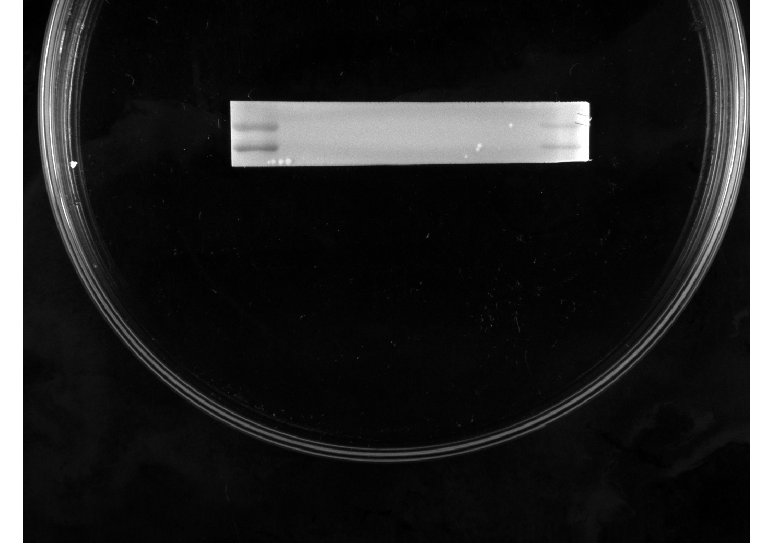

Supplement: Supplementary file 2 — Supporting Information 2 File S1: original data.zip This file contains the original Western Blot raw data images related to the study. [file MI-2026-7332100-s003.zip › 20240807/1/230703-HUVEC/mo2/1/tublin 1-3.tif]

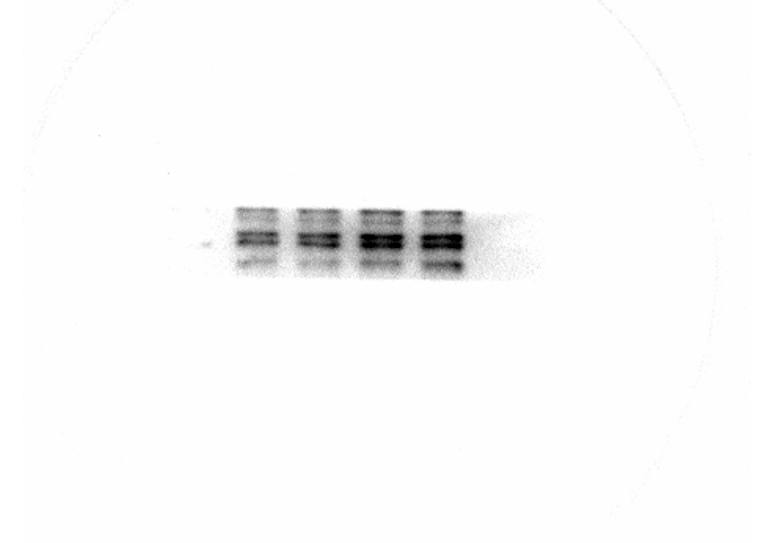

Supplement: Supplementary file 2 — Supporting Information 2 File S1: original data.zip This file contains the original Western Blot raw data images related to the study. [file MI-2026-7332100-s003.zip › 20240807/1/230703-HUVEC/mo3/1/cylin d3 1-1.tif]

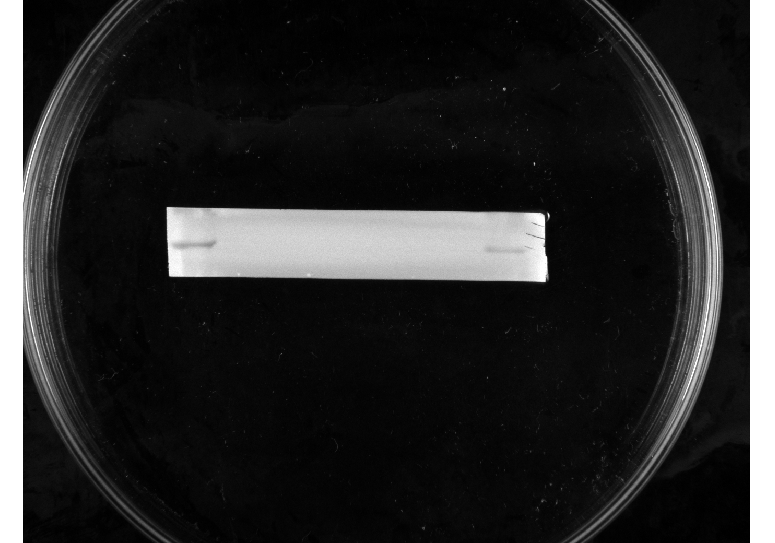

Supplement: Supplementary file 2 — Supporting Information 2 File S1: original data.zip This file contains the original Western Blot raw data images related to the study. [file MI-2026-7332100-s003.zip › 20240807/1/230703-HUVEC/mo3/1/cylin d3 1-3.tif]

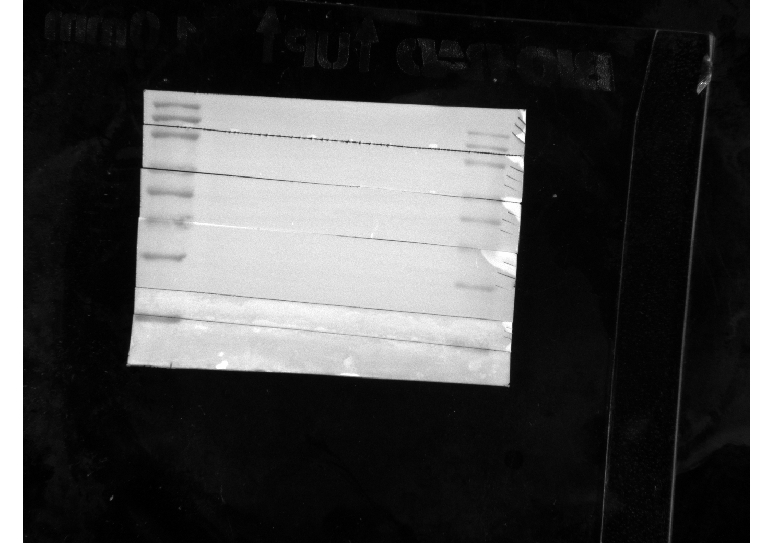

Supplement: Supplementary file 2 — Supporting Information 2 File S1: original data.zip This file contains the original Western Blot raw data images related to the study. [file MI-2026-7332100-s003.zip › 20240807/1/230703-HUVEC/mo3/1/H-n3.tif]

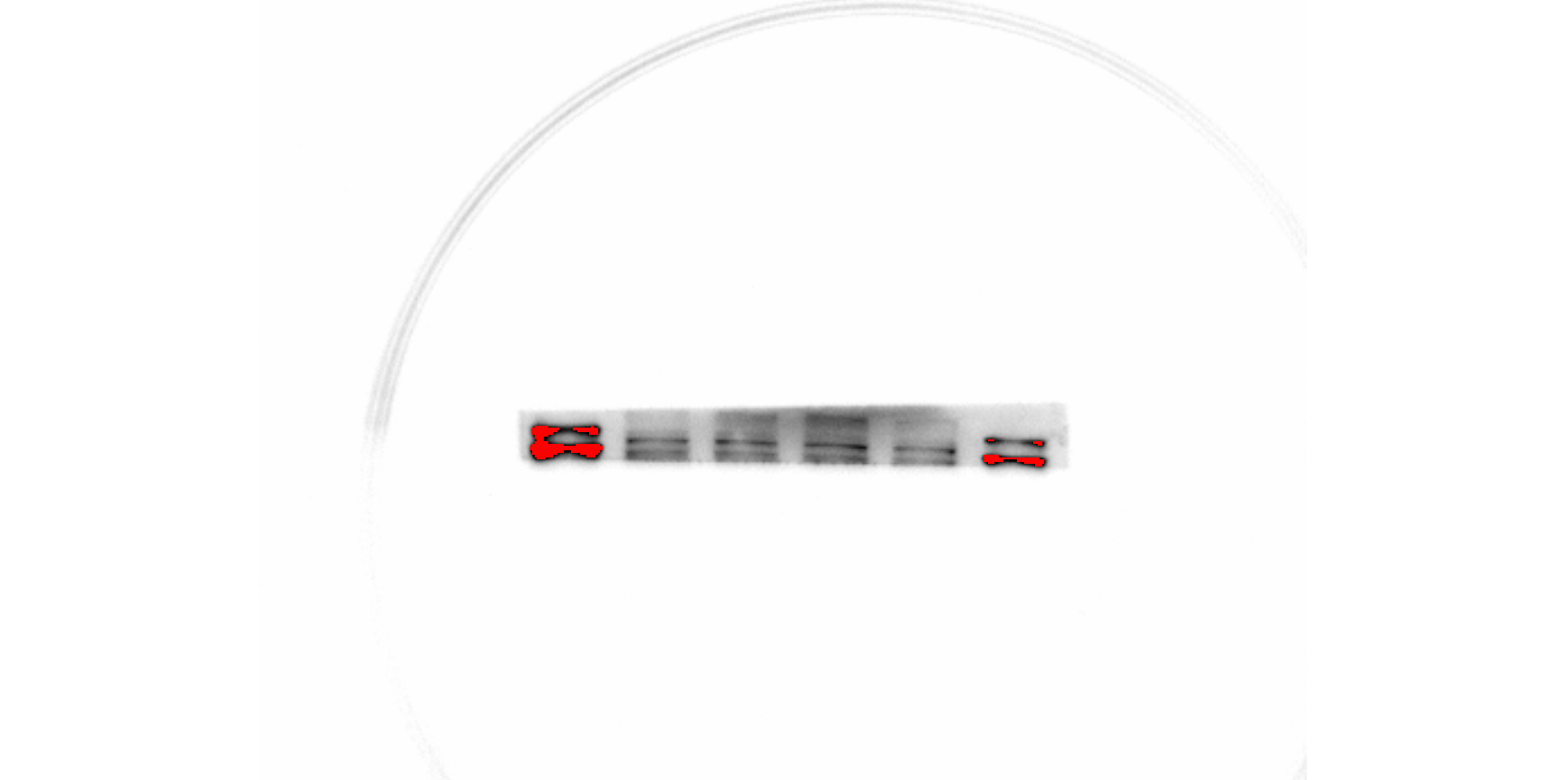

Supplement: Supplementary file 2 — Supporting Information 2 File S1: original data.zip This file contains the original Western Blot raw data images related to the study. [file MI-2026-7332100-s003.zip › 20240807/1/230703-HUVEC/mo3/1/jak1 1-1.tif]

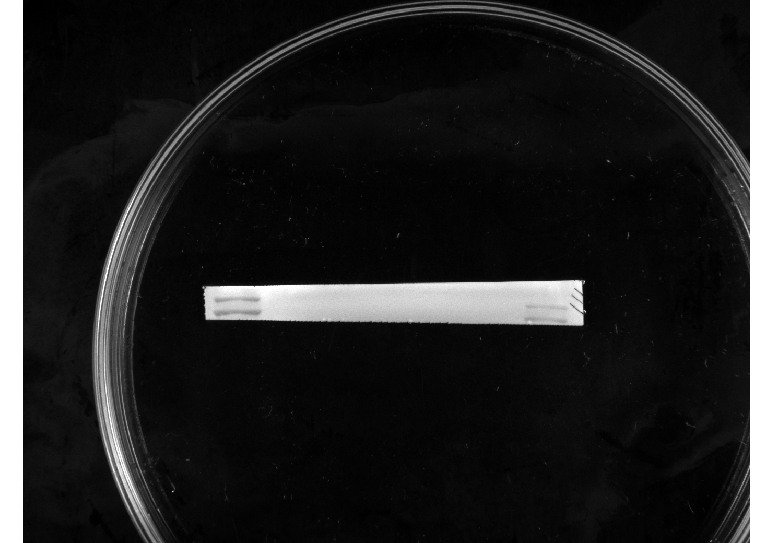

Supplement: Supplementary file 2 — Supporting Information 2 File S1: original data.zip This file contains the original Western Blot raw data images related to the study. [file MI-2026-7332100-s003.zip › 20240807/1/230703-HUVEC/mo3/1/jak1 1-3.tif]

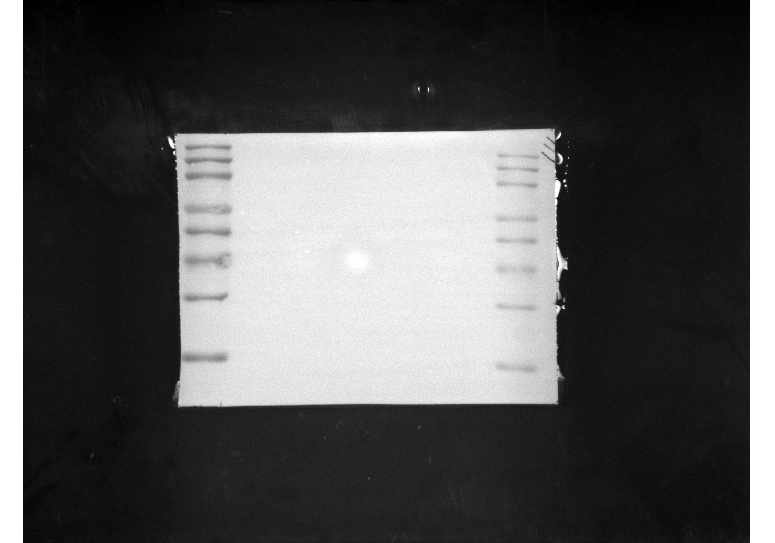

Supplement: Supplementary file 2 — Supporting Information 2 File S1: original data.zip This file contains the original Western Blot raw data images related to the study. [file MI-2026-7332100-s003.zip › 20240807/1/230703-HUVEC/mo3/1/paimo3.tif]

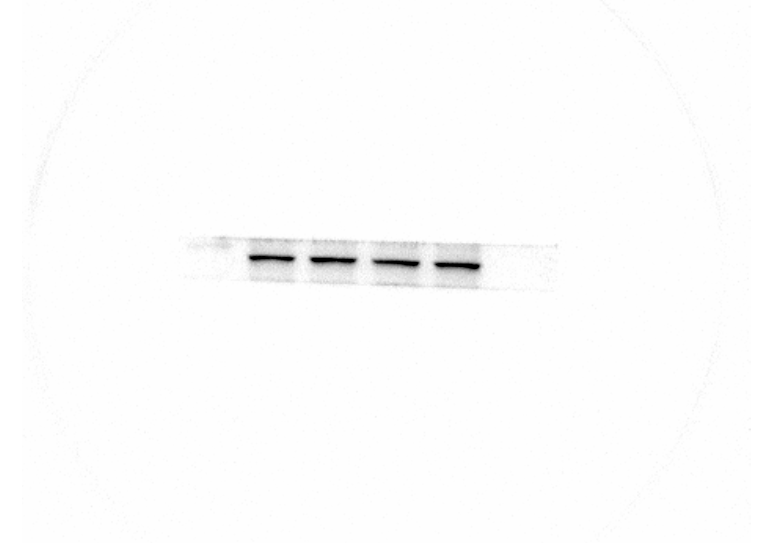

Supplement: Supplementary file 2 — Supporting Information 2 File S1: original data.zip This file contains the original Western Blot raw data images related to the study. [file MI-2026-7332100-s003.zip › 20240807/1/230703-HUVEC/mo3/1/stat3 1-1.tif]

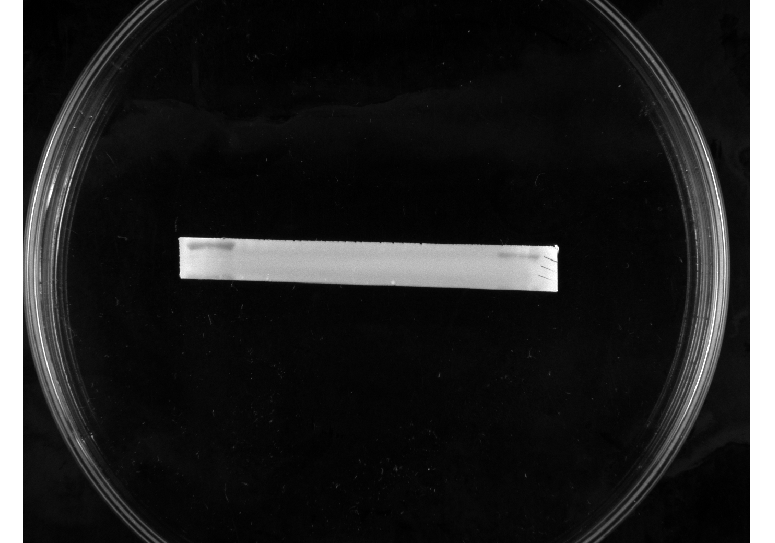

Supplement: Supplementary file 2 — Supporting Information 2 File S1: original data.zip This file contains the original Western Blot raw data images related to the study. [file MI-2026-7332100-s003.zip › 20240807/1/230703-HUVEC/mo3/1/stat3 1-3.tif]

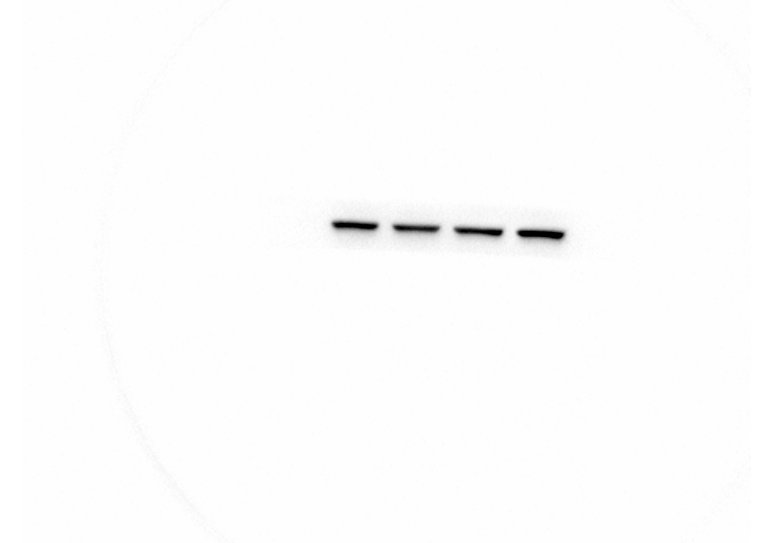

Supplement: Supplementary file 2 — Supporting Information 2 File S1: original data.zip This file contains the original Western Blot raw data images related to the study. [file MI-2026-7332100-s003.zip › 20240807/1/230703-HUVEC/mo3/1/tublin 1-1.tif]

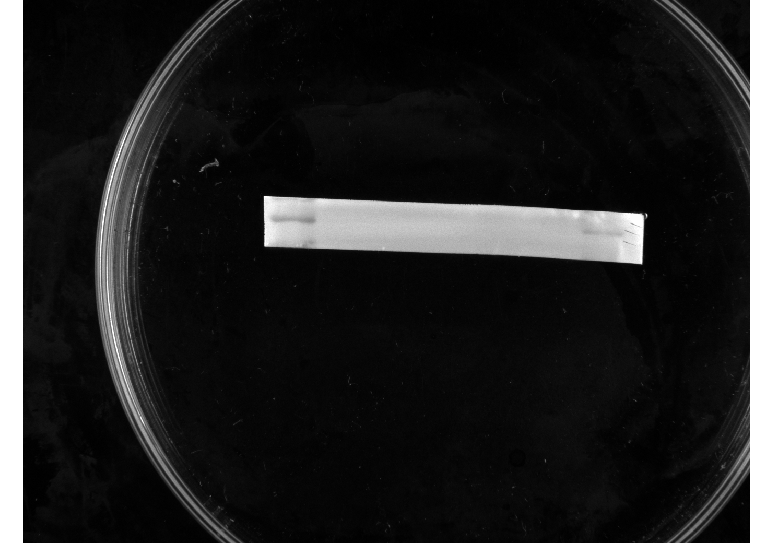

Supplement: Supplementary file 2 — Supporting Information 2 File S1: original data.zip This file contains the original Western Blot raw data images related to the study. [file MI-2026-7332100-s003.zip › 20240807/1/230703-HUVEC/mo3/1/tublin 1-3.tif]

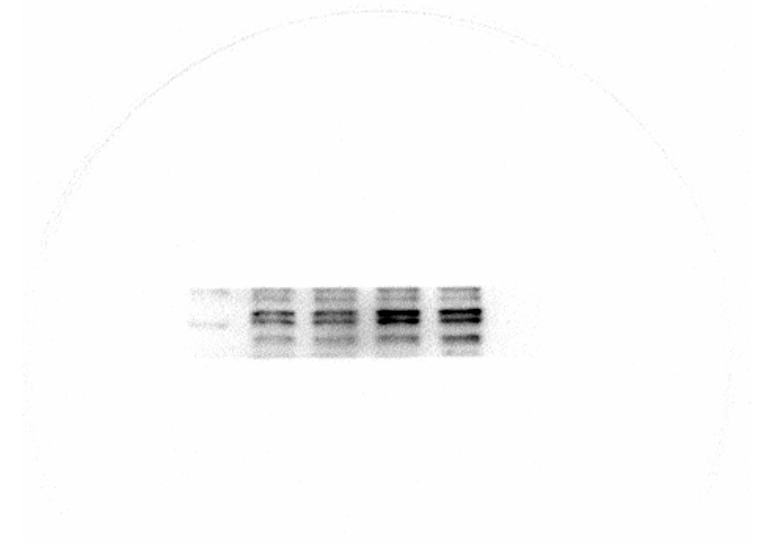

Supplement: Supplementary file 2 — Supporting Information 2 File S1: original data.zip This file contains the original Western Blot raw data images related to the study. [file MI-2026-7332100-s003.zip › 20240807/1/230703-HUVEC/mo4/1/cylin d3 1-1.tif]

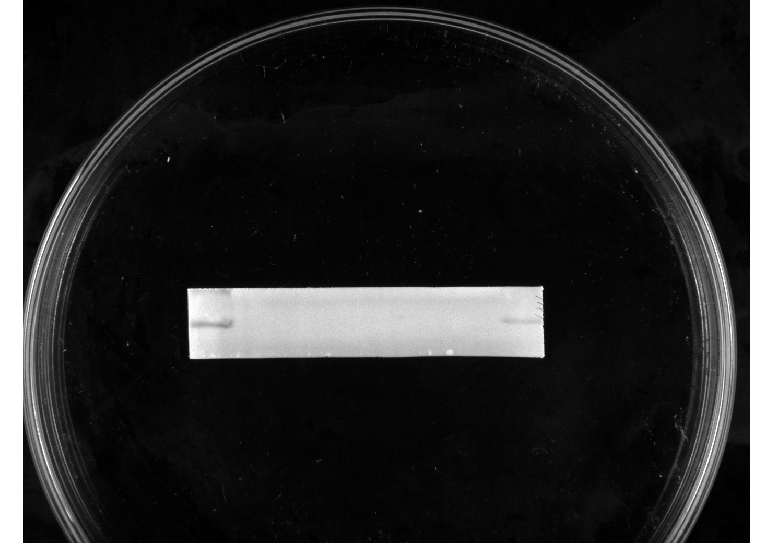

Supplement: Supplementary file 2 — Supporting Information 2 File S1: original data.zip This file contains the original Western Blot raw data images related to the study. [file MI-2026-7332100-s003.zip › 20240807/1/230703-HUVEC/mo4/1/cylin d3 1-3.tif]

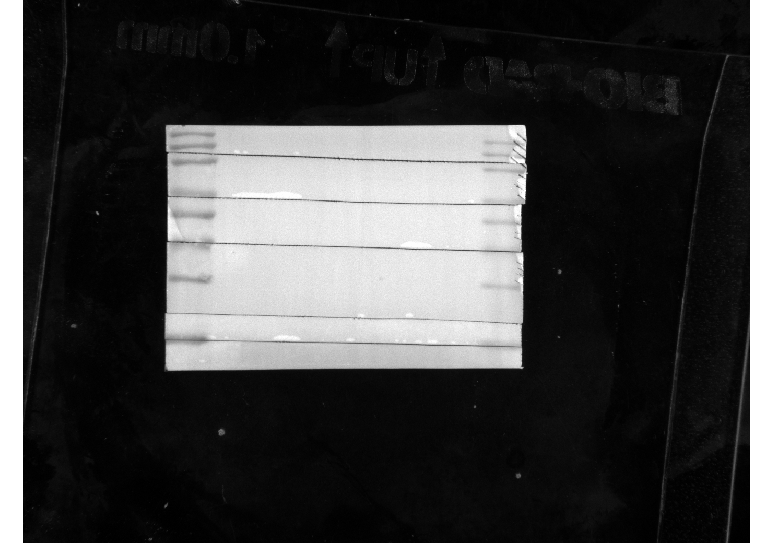

Supplement: Supplementary file 2 — Supporting Information 2 File S1: original data.zip This file contains the original Western Blot raw data images related to the study. [file MI-2026-7332100-s003.zip › 20240807/1/230703-HUVEC/mo4/1/H-n4.tif]

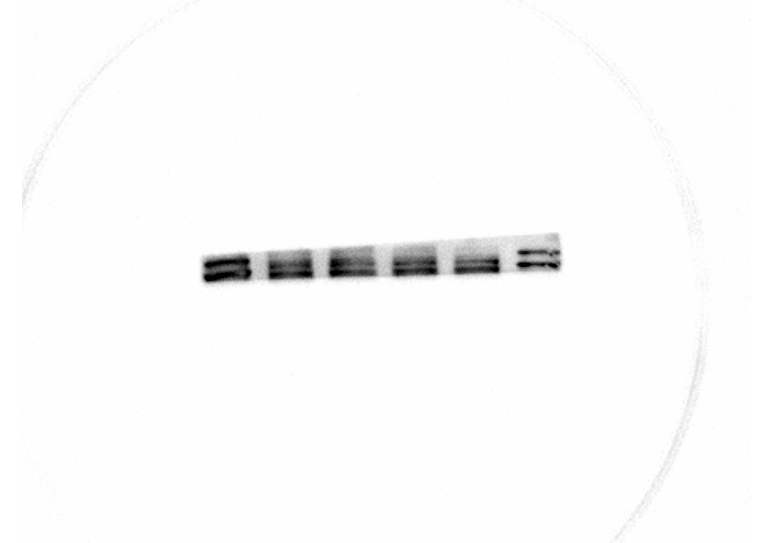

Supplement: Supplementary file 2 — Supporting Information 2 File S1: original data.zip This file contains the original Western Blot raw data images related to the study. [file MI-2026-7332100-s003.zip › 20240807/1/230703-HUVEC/mo4/1/jak1 1-1.tif]

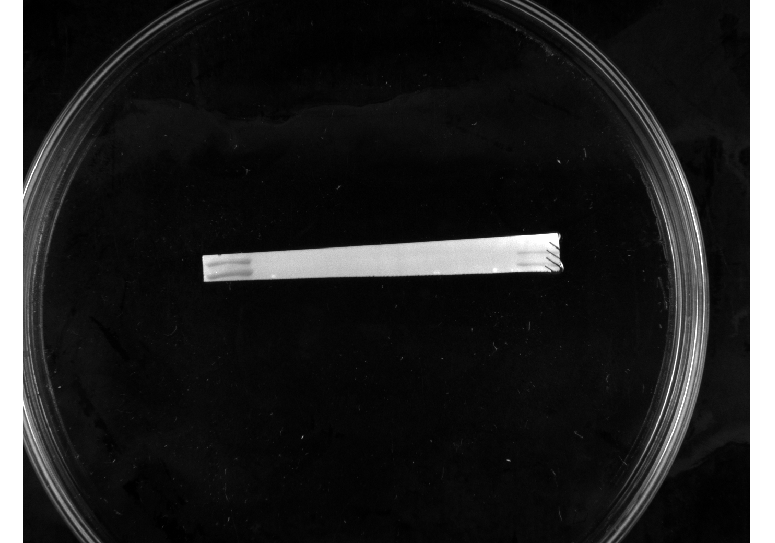

Supplement: Supplementary file 2 — Supporting Information 2 File S1: original data.zip This file contains the original Western Blot raw data images related to the study. [file MI-2026-7332100-s003.zip › 20240807/1/230703-HUVEC/mo4/1/jak1 1-3.tif]

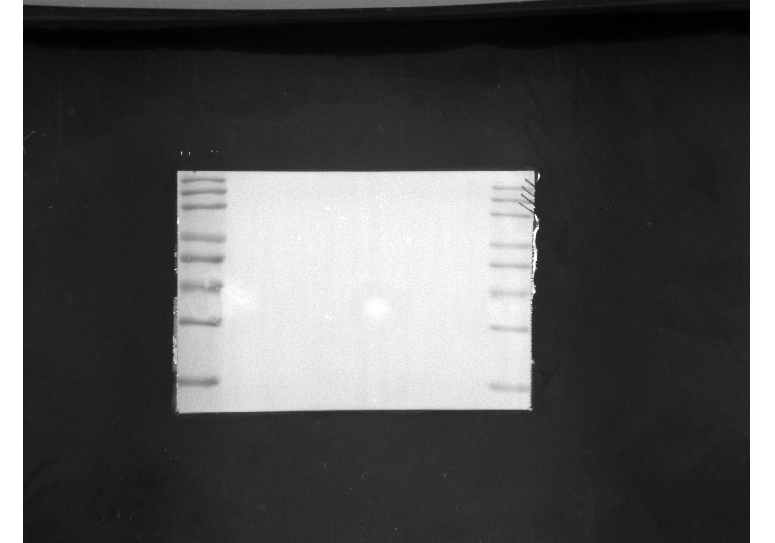

Supplement: Supplementary file 2 — Supporting Information 2 File S1: original data.zip This file contains the original Western Blot raw data images related to the study. [file MI-2026-7332100-s003.zip › 20240807/1/230703-HUVEC/mo4/1/paimo4.tif]

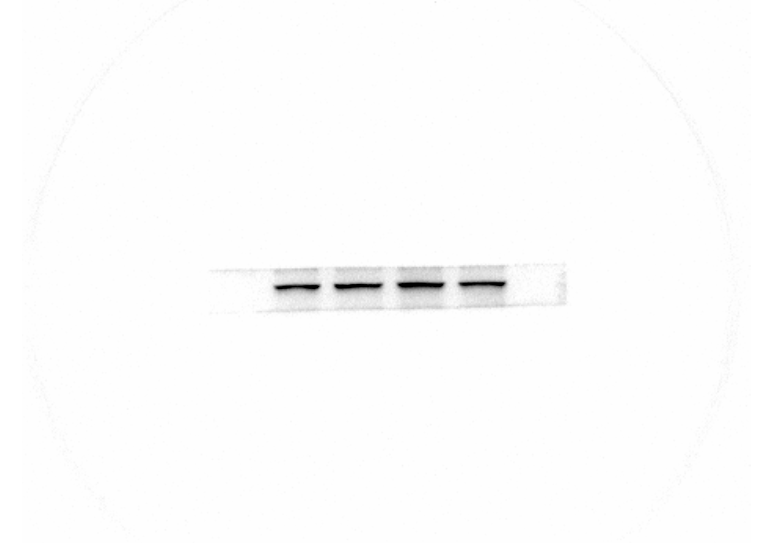

Supplement: Supplementary file 2 — Supporting Information 2 File S1: original data.zip This file contains the original Western Blot raw data images related to the study. [file MI-2026-7332100-s003.zip › 20240807/1/230703-HUVEC/mo4/1/stat3 1-1.tif]

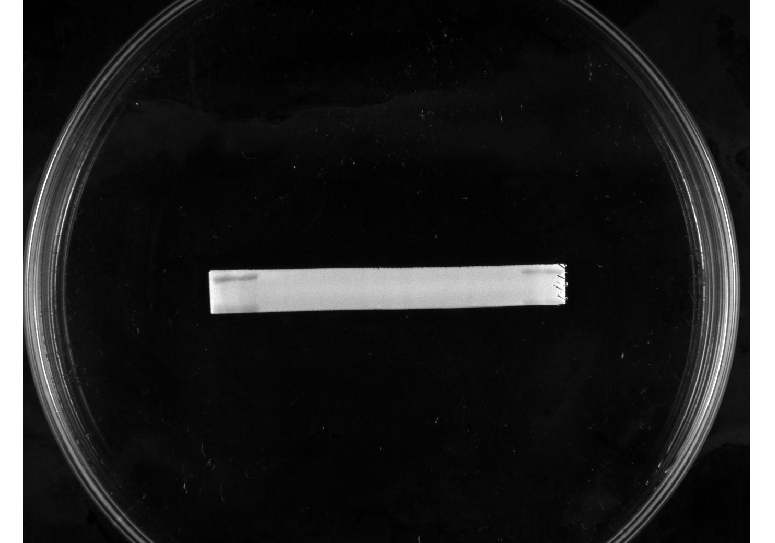

Supplement: Supplementary file 2 — Supporting Information 2 File S1: original data.zip This file contains the original Western Blot raw data images related to the study. [file MI-2026-7332100-s003.zip › 20240807/1/230703-HUVEC/mo4/1/stat3 1-3.tif]

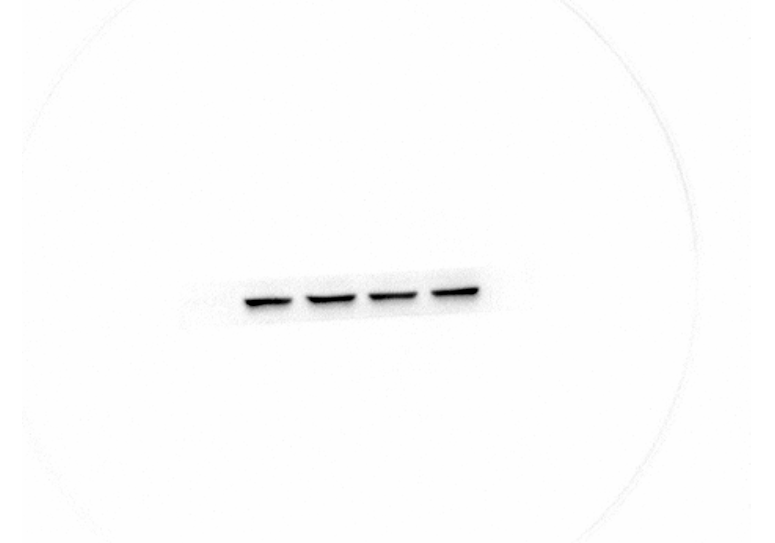

Supplement: Supplementary file 2 — Supporting Information 2 File S1: original data.zip This file contains the original Western Blot raw data images related to the study. [file MI-2026-7332100-s003.zip › 20240807/1/230703-HUVEC/mo4/1/tublin 1-1.tif]

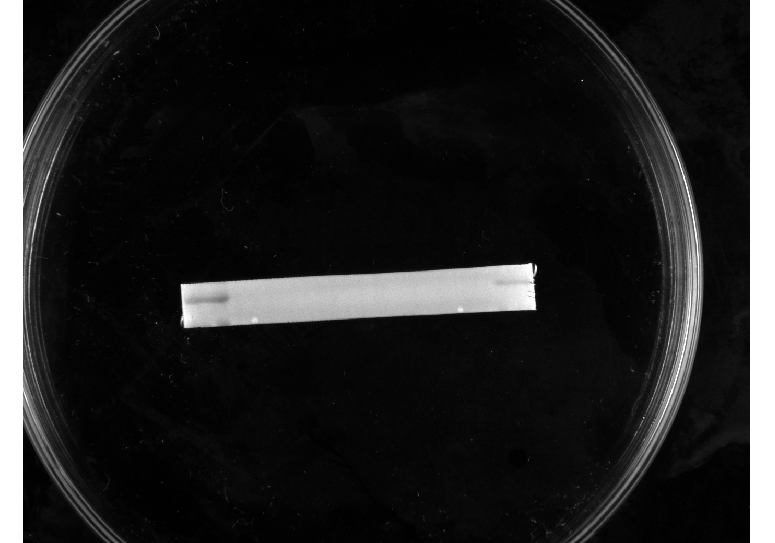

Supplement: Supplementary file 2 — Supporting Information 2 File S1: original data.zip This file contains the original Western Blot raw data images related to the study. [file MI-2026-7332100-s003.zip › 20240807/1/230703-HUVEC/mo4/1/tublin 1-3.tif]

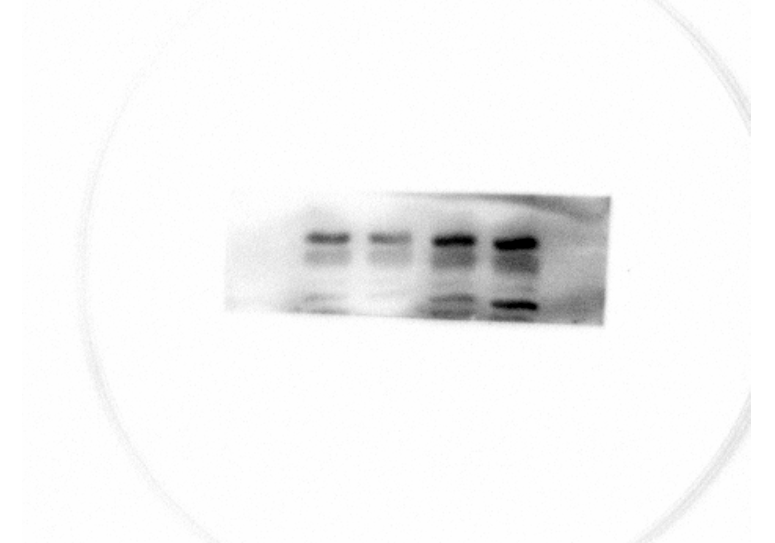

Supplement: Supplementary file 2 — Supporting Information 2 File S1: original data.zip This file contains the original Western Blot raw data images related to the study. [file MI-2026-7332100-s003.zip › 20240807/1/230706-HUVEC/mo1/1/cyclin D1 1-1.tif]

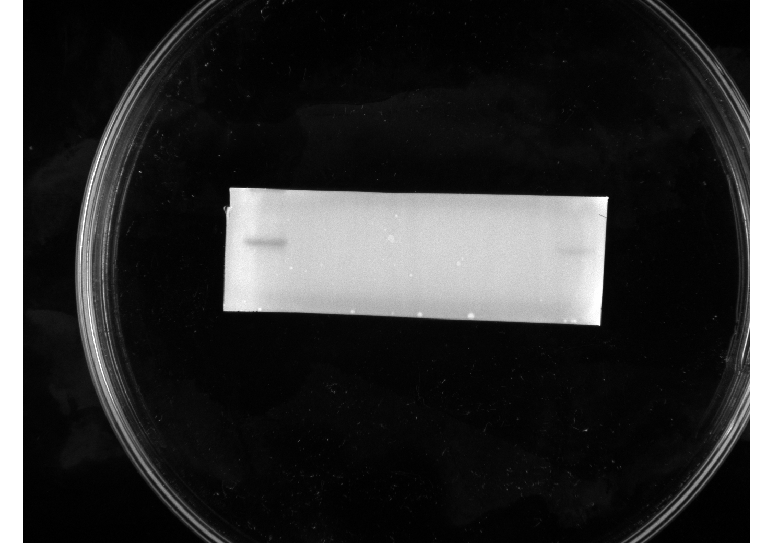

Supplement: Supplementary file 2 — Supporting Information 2 File S1: original data.zip This file contains the original Western Blot raw data images related to the study. [file MI-2026-7332100-s003.zip › 20240807/1/230706-HUVEC/mo1/1/cyclin D1 1-3.tif]

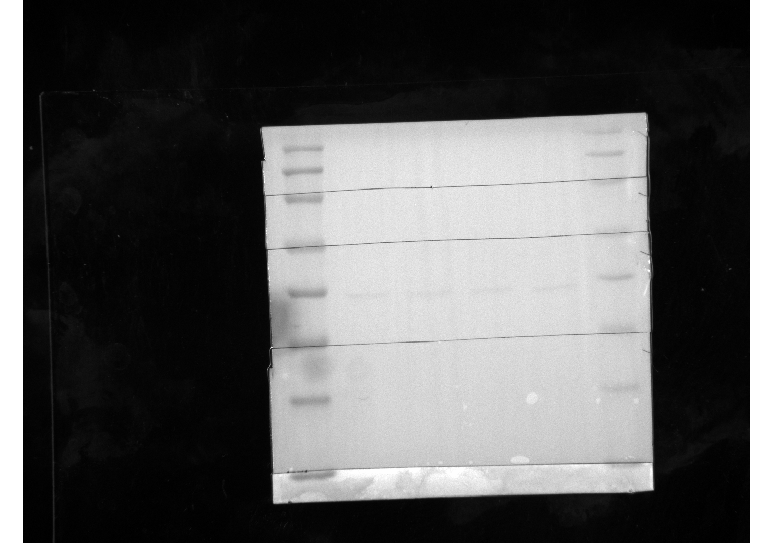

Supplement: Supplementary file 2 — Supporting Information 2 File S1: original data.zip This file contains the original Western Blot raw data images related to the study. [file MI-2026-7332100-s003.zip › 20240807/1/230706-HUVEC/mo1/1/H-n1.tif]

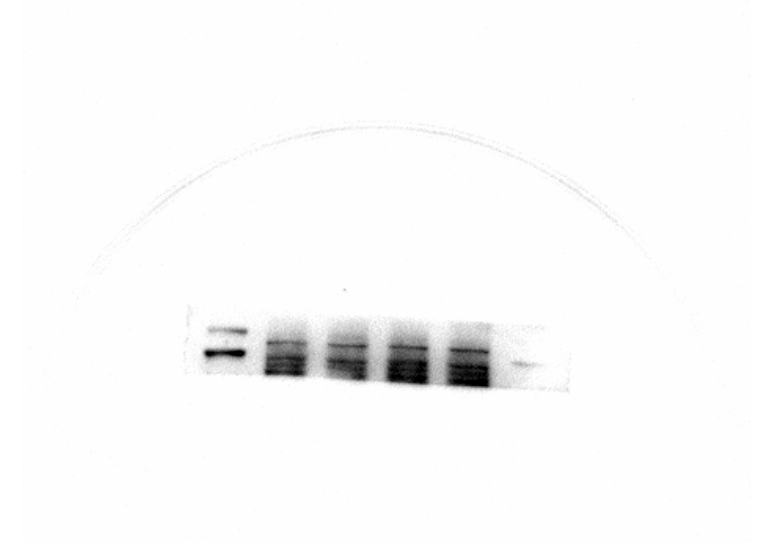

Supplement: Supplementary file 2 — Supporting Information 2 File S1: original data.zip This file contains the original Western Blot raw data images related to the study. [file MI-2026-7332100-s003.zip › 20240807/1/230706-HUVEC/mo1/1/JAK1 1-1.tif]

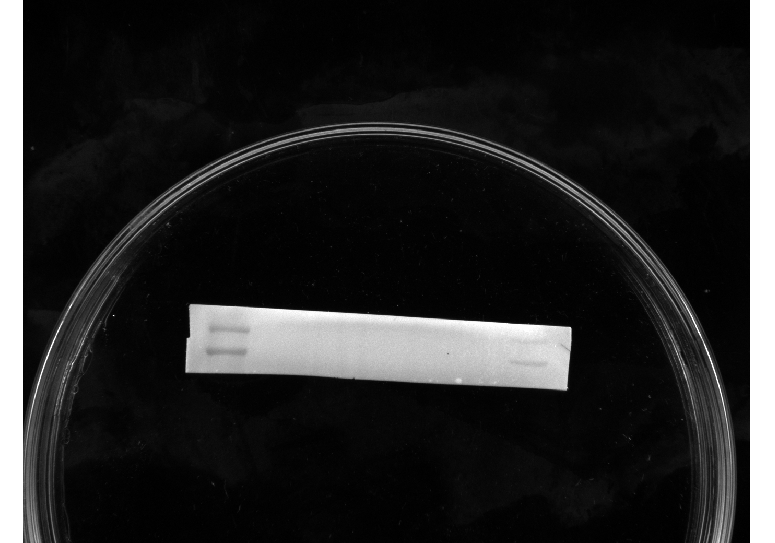

Supplement: Supplementary file 2 — Supporting Information 2 File S1: original data.zip This file contains the original Western Blot raw data images related to the study. [file MI-2026-7332100-s003.zip › 20240807/1/230706-HUVEC/mo1/1/JAK1 1-3.tif]

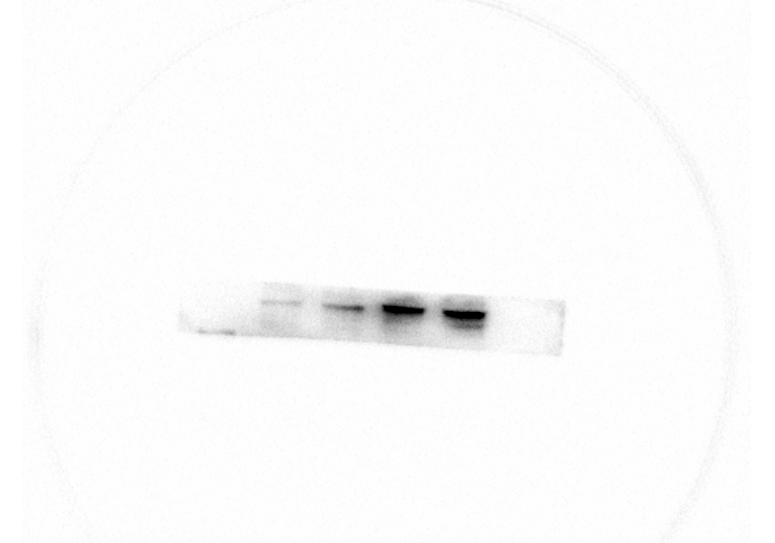

Supplement: Supplementary file 2 — Supporting Information 2 File S1: original data.zip This file contains the original Western Blot raw data images related to the study. [file MI-2026-7332100-s003.zip › 20240807/1/230706-HUVEC/mo1/1/P-STAT3 1-1.tif]

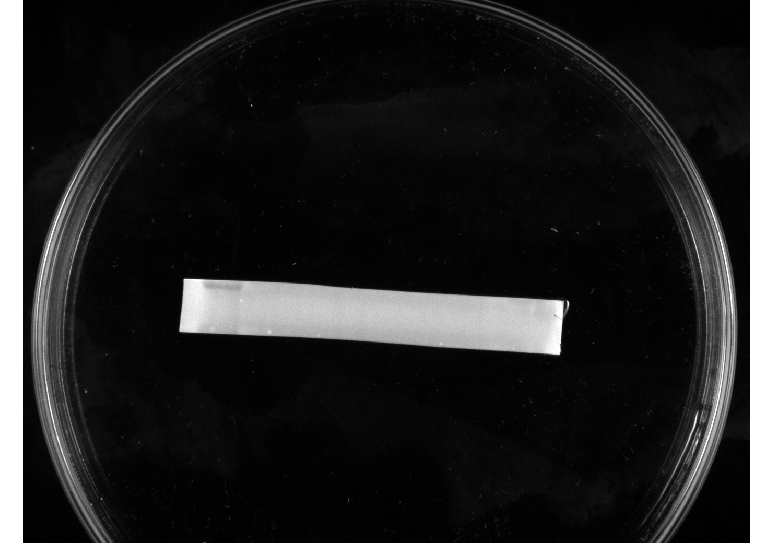

Supplement: Supplementary file 2 — Supporting Information 2 File S1: original data.zip This file contains the original Western Blot raw data images related to the study. [file MI-2026-7332100-s003.zip › 20240807/1/230706-HUVEC/mo1/1/P-STAT3 1-3.tif]

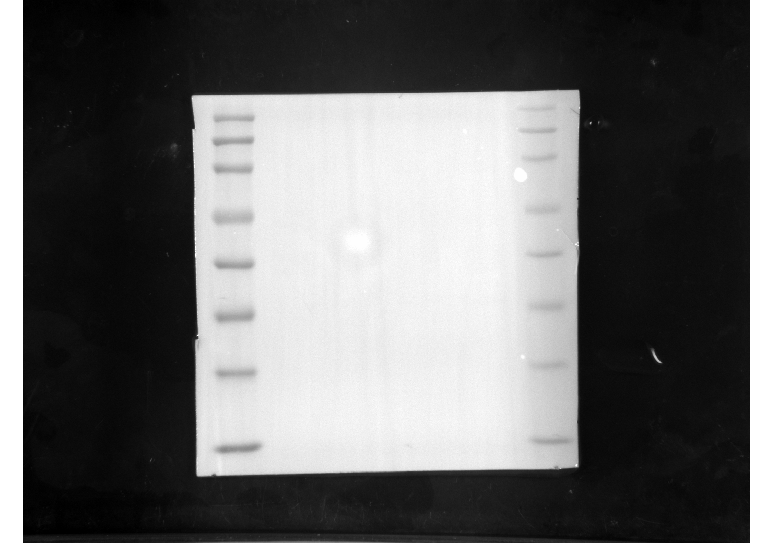

Supplement: Supplementary file 2 — Supporting Information 2 File S1: original data.zip This file contains the original Western Blot raw data images related to the study. [file MI-2026-7332100-s003.zip › 20240807/1/230706-HUVEC/mo1/1/paizhao1.tif]

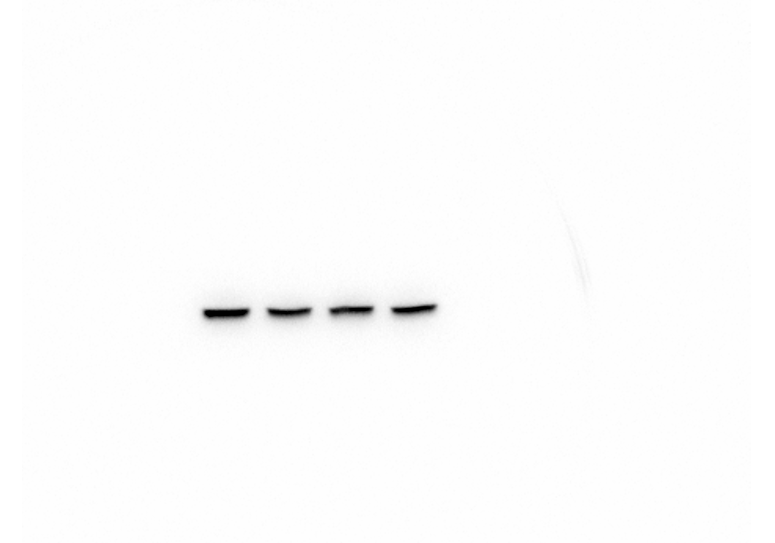

Supplement: Supplementary file 2 — Supporting Information 2 File S1: original data.zip This file contains the original Western Blot raw data images related to the study. [file MI-2026-7332100-s003.zip › 20240807/1/230706-HUVEC/mo1/1/tublin 1-1.tif]

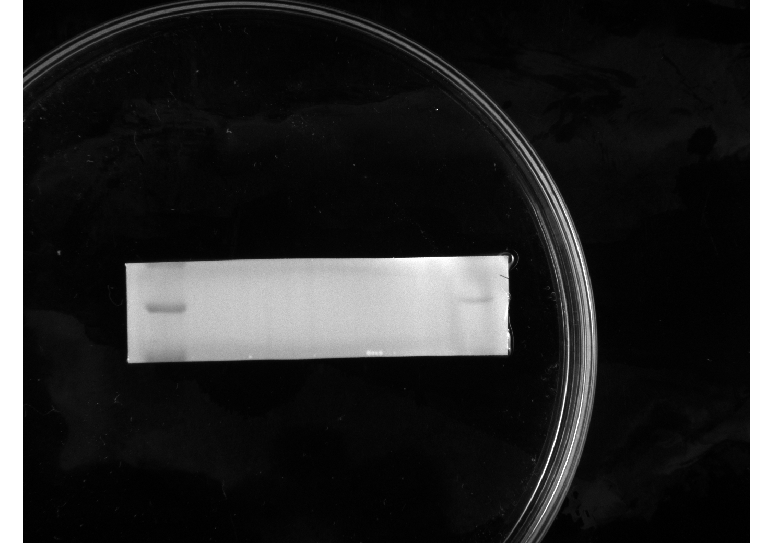

Supplement: Supplementary file 2 — Supporting Information 2 File S1: original data.zip This file contains the original Western Blot raw data images related to the study. [file MI-2026-7332100-s003.zip › 20240807/1/230706-HUVEC/mo1/1/tublin 1-3.tif]

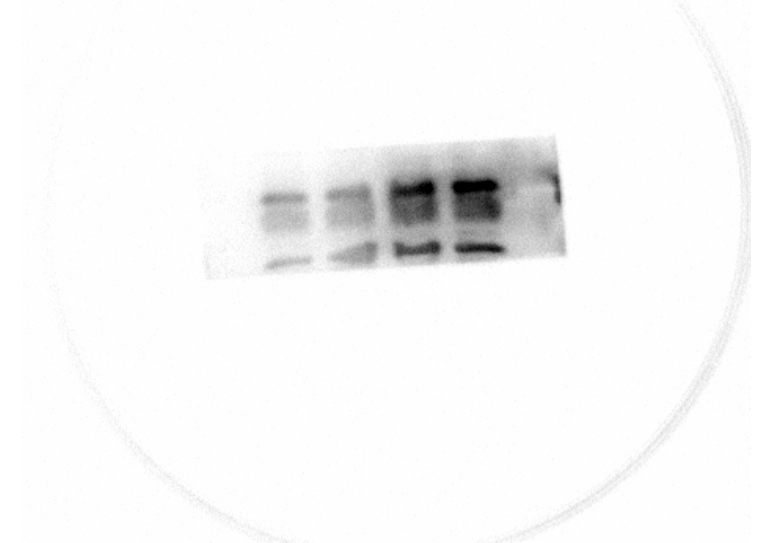

Supplement: Supplementary file 2 — Supporting Information 2 File S1: original data.zip This file contains the original Western Blot raw data images related to the study. [file MI-2026-7332100-s003.zip › 20240807/1/230706-HUVEC/mo2/1/cyclin D1 1-1.tif]

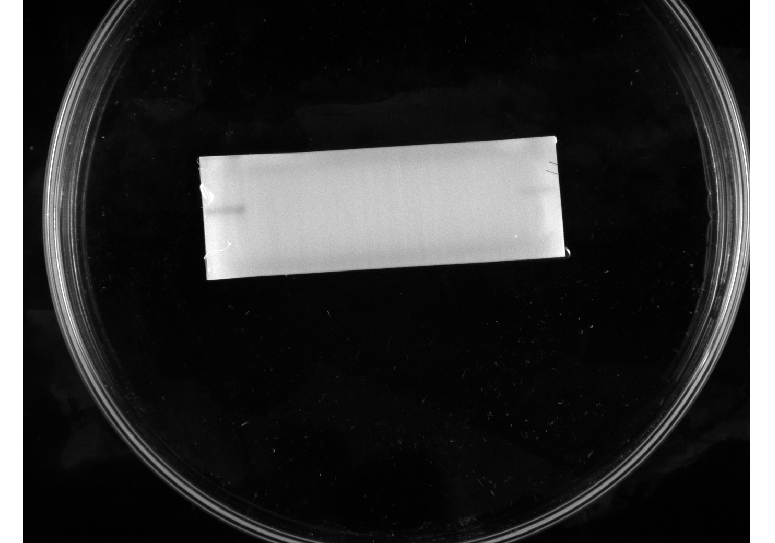

Supplement: Supplementary file 2 — Supporting Information 2 File S1: original data.zip This file contains the original Western Blot raw data images related to the study. [file MI-2026-7332100-s003.zip › 20240807/1/230706-HUVEC/mo2/1/cyclin D1 1-3.tif]

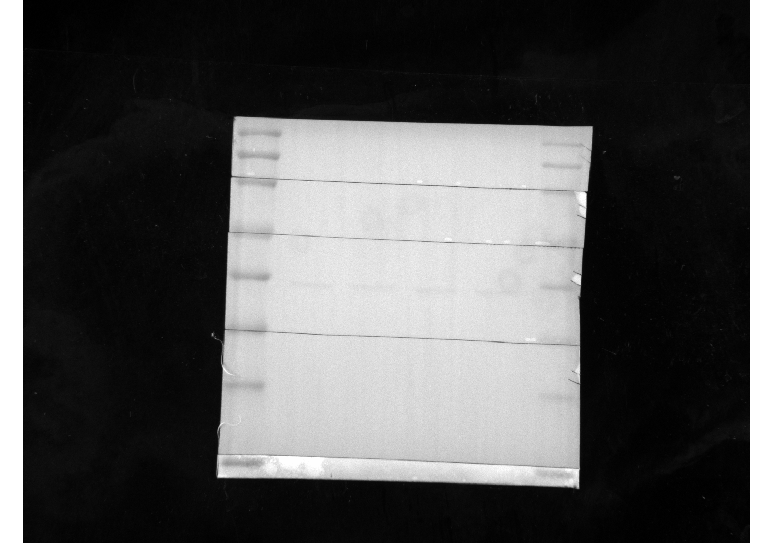

Supplement: Supplementary file 2 — Supporting Information 2 File S1: original data.zip This file contains the original Western Blot raw data images related to the study. [file MI-2026-7332100-s003.zip › 20240807/1/230706-HUVEC/mo2/1/H-n2.tif]

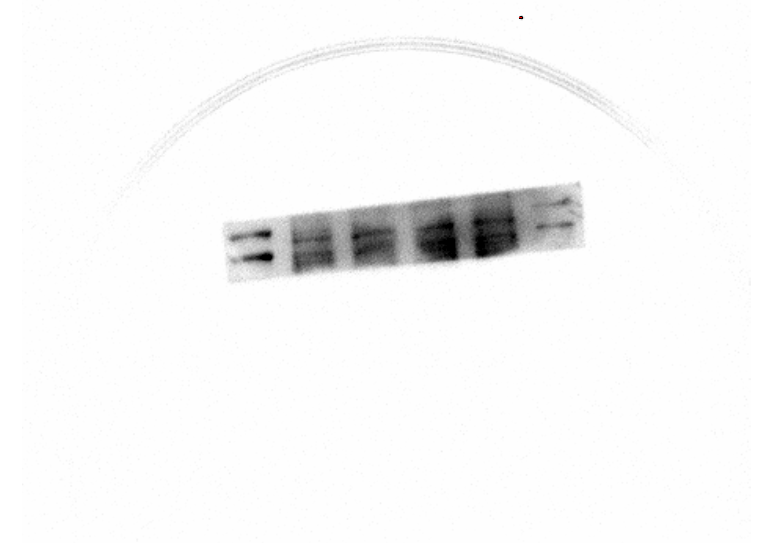

Supplement: Supplementary file 2 — Supporting Information 2 File S1: original data.zip This file contains the original Western Blot raw data images related to the study. [file MI-2026-7332100-s003.zip › 20240807/1/230706-HUVEC/mo2/1/JAK1 1-1.tif]

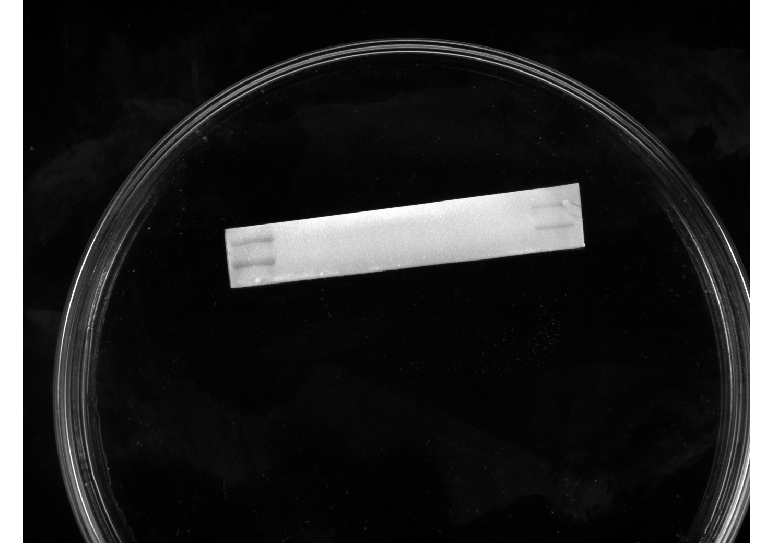

Supplement: Supplementary file 2 — Supporting Information 2 File S1: original data.zip This file contains the original Western Blot raw data images related to the study. [file MI-2026-7332100-s003.zip › 20240807/1/230706-HUVEC/mo2/1/JAK1 1-3.tif]

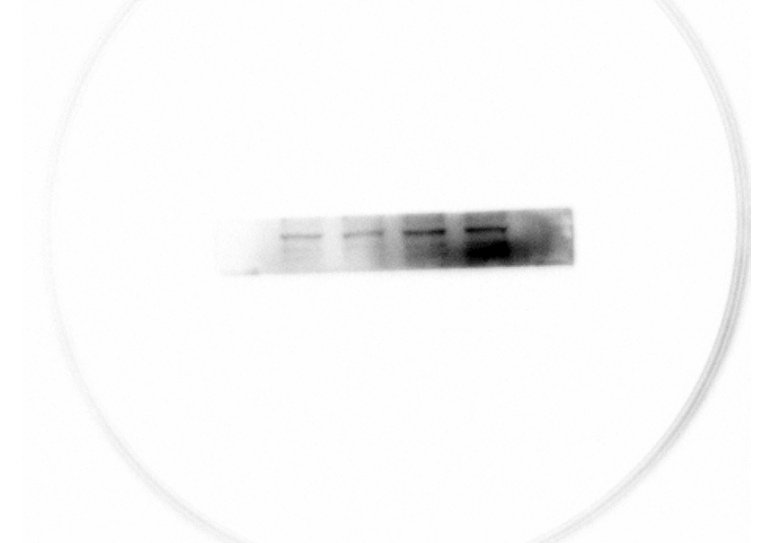

Supplement: Supplementary file 2 — Supporting Information 2 File S1: original data.zip This file contains the original Western Blot raw data images related to the study. [file MI-2026-7332100-s003.zip › 20240807/1/230706-HUVEC/mo2/1/P-STAT3 1-1.tif]

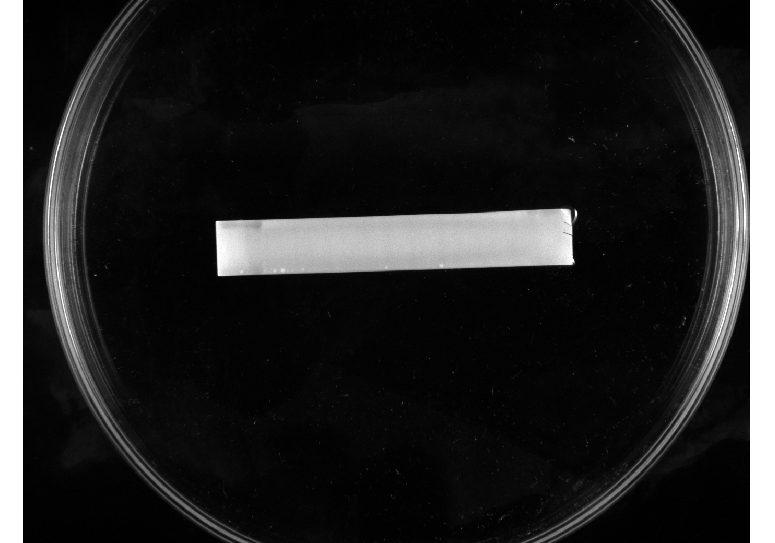

Supplement: Supplementary file 2 — Supporting Information 2 File S1: original data.zip This file contains the original Western Blot raw data images related to the study. [file MI-2026-7332100-s003.zip › 20240807/1/230706-HUVEC/mo2/1/P-STAT3 1-3.tif]

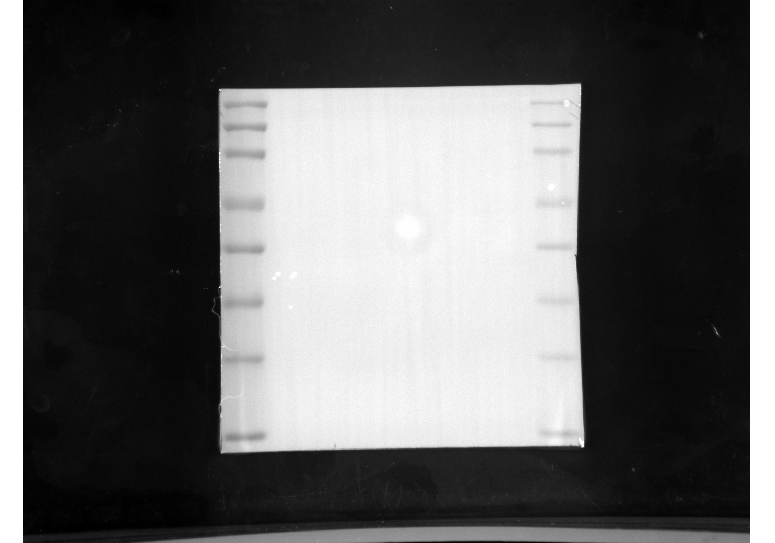

Supplement: Supplementary file 2 — Supporting Information 2 File S1: original data.zip This file contains the original Western Blot raw data images related to the study. [file MI-2026-7332100-s003.zip › 20240807/1/230706-HUVEC/mo2/1/paizhao2.tif]

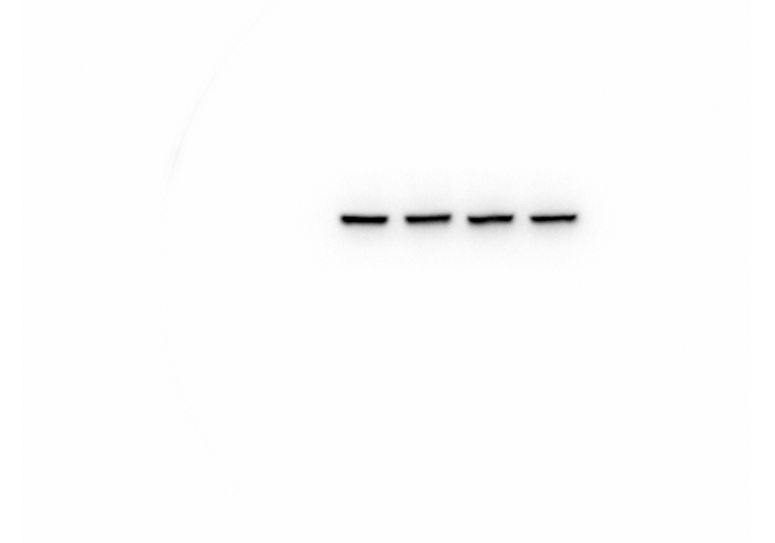

Supplement: Supplementary file 2 — Supporting Information 2 File S1: original data.zip This file contains the original Western Blot raw data images related to the study. [file MI-2026-7332100-s003.zip › 20240807/1/230706-HUVEC/mo2/1/tublin 1-1.tif]

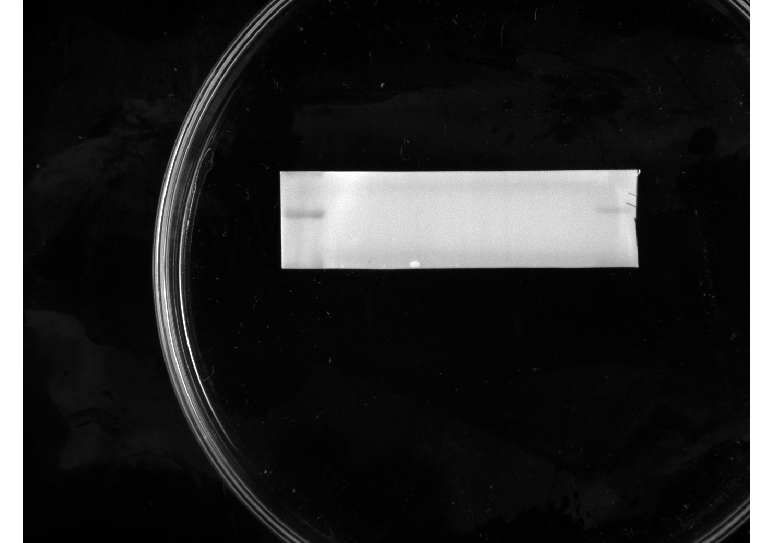

Supplement: Supplementary file 2 — Supporting Information 2 File S1: original data.zip This file contains the original Western Blot raw data images related to the study. [file MI-2026-7332100-s003.zip › 20240807/1/230706-HUVEC/mo2/1/tublin 1-3.tif]

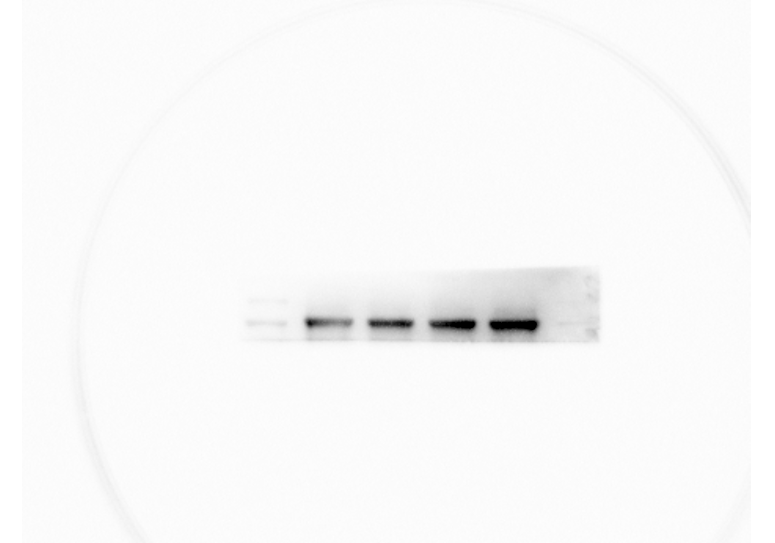

Supplement: Supplementary file 2 — Supporting Information 2 File S1: original data.zip This file contains the original Western Blot raw data images related to the study. [file MI-2026-7332100-s003.zip › 20240807/1/230706-HUVEC/mo3/1/CD31 1-1.tif]

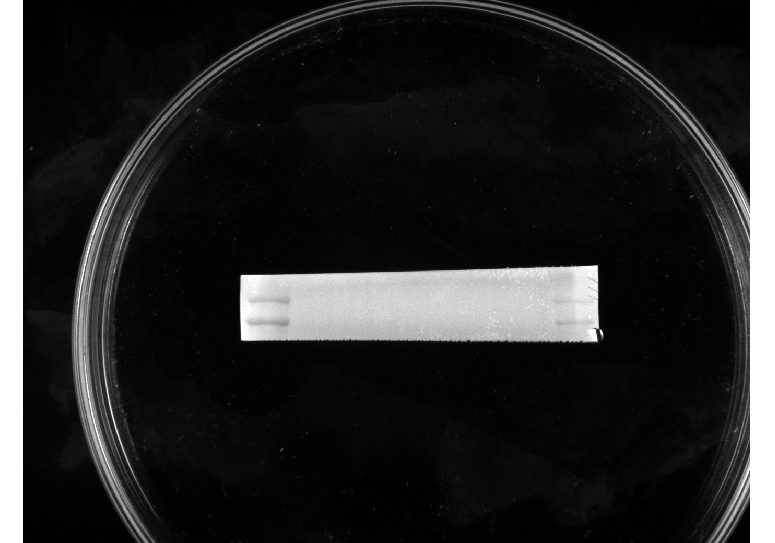

Supplement: Supplementary file 2 — Supporting Information 2 File S1: original data.zip This file contains the original Western Blot raw data images related to the study. [file MI-2026-7332100-s003.zip › 20240807/1/230706-HUVEC/mo3/1/CD31 1-3.tif]

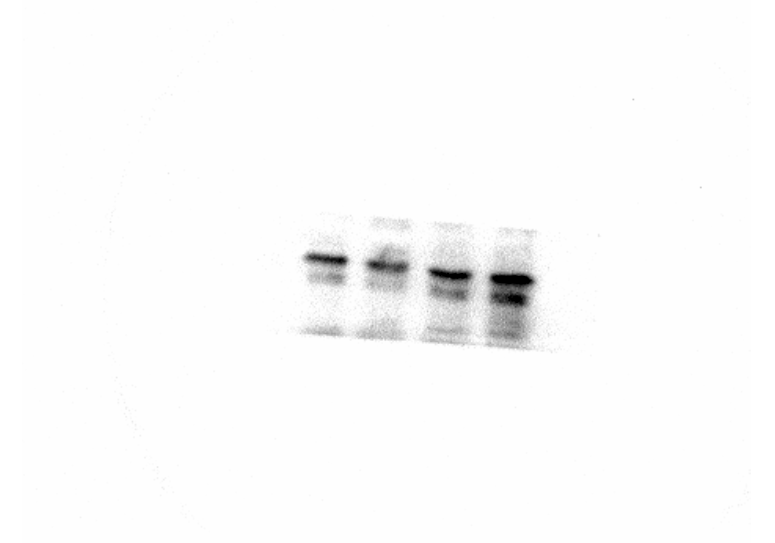

Supplement: Supplementary file 2 — Supporting Information 2 File S1: original data.zip This file contains the original Western Blot raw data images related to the study. [file MI-2026-7332100-s003.zip › 20240807/1/230706-HUVEC/mo3/1/cyclin D3 1-1.tif]

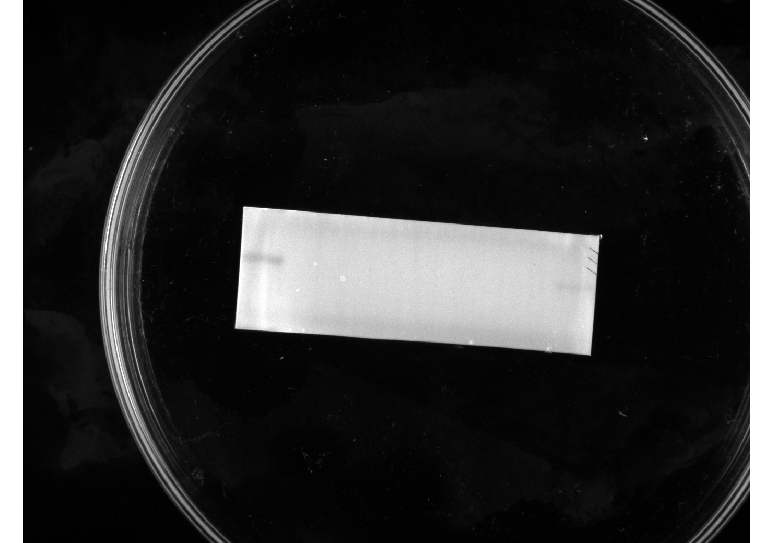

Supplement: Supplementary file 2 — Supporting Information 2 File S1: original data.zip This file contains the original Western Blot raw data images related to the study. [file MI-2026-7332100-s003.zip › 20240807/1/230706-HUVEC/mo3/1/cyclin D3 1-3.tif]

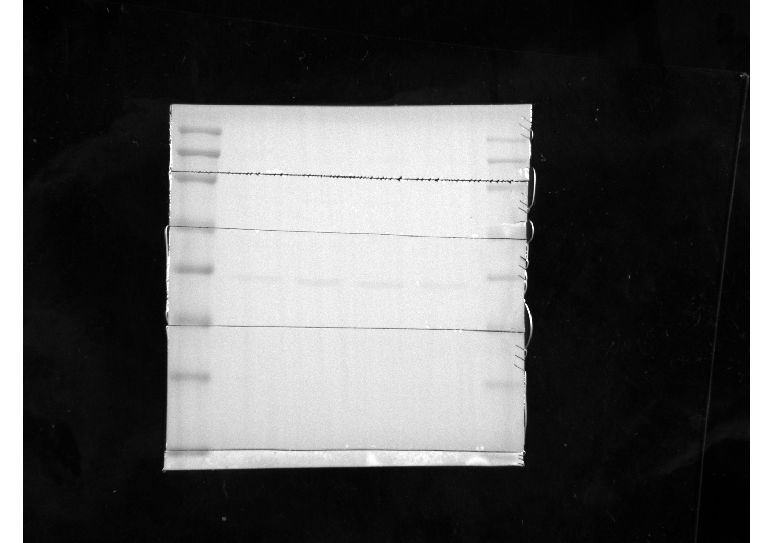

Supplement: Supplementary file 2 — Supporting Information 2 File S1: original data.zip This file contains the original Western Blot raw data images related to the study. [file MI-2026-7332100-s003.zip › 20240807/1/230706-HUVEC/mo3/1/H-n3.tif]
